# Assessment of the antioxidant and antibacterial activities of different olive processing wastewaters

**DOI:** 10.1371/journal.pone.0182622

**Published:** 2017-09-05

**Authors:** Majdouline Belaqziz, Shiau Pin Tan, Abdelilah El-Abbassi, Hajar Kiai, Abdellatif Hafidi, Orla O’Donovan, Peter McLoughlin

**Affiliations:** 1 Center of Analysis and Characterization, Cadi Ayyad University, Marrakech, Morocco; 2 The Pharmaceutical & Molecular Biotechnology Research Centre (PMBRC), Department of Science, Waterford Institute of Technology, Waterford, Ireland; 3 Food Sciences laboratory, Department of Biology, Faculty of Sciences–Semlalia, Cadi Ayyad University, Marrakech, Morocco; University of Palermo, ITALY

## Abstract

Olive processing wastewaters (OPW), namely olive mill wastewater (OMW) and table-olive wastewaters (TOW) were evaluated for their antibacterial activity against five Gram-positive and two Gram-negative bacteria using the standard disc diffusion and thin layer chromatography (TLC)-bioautography assays. Disc diffusion screening and bioautography of OMW were compared to the phenolic extracts of table-olive brines. Positive activity against *S*. *aureus* was demonstrated. The optimization of chromatographic separation revealed that hexane/acetone in the ratio of 4:6 was the most effective for phenolic compounds separation. A HPLC-MS analysis was performed showing that only two compounds, hydroxytyrosol and tyrosol, were the predominant phenolic compounds in all OPW. The phenolic extract of OMW generated by a semi-modern process showed the highest free radical-scavenging activity (DPPH assay) compared to the other phenolic extracts. It is apparent from the present study that OPW are a rich source of antioxidants suitable for use in food, cosmetic or pharmaceutical applications.

## Introduction

Olive fruits are used in the agro-industry mainly for the preparation of table olives and oil extraction. The olive oil extraction generates an effluent formed by the combination of the water content of the olive fruit with the water generated from the washing and processing of the olives. This effluent is commonly known as olive mill wastewater (OMW). This effluent is one of the most environmentally concerning food processing effluents in the Mediterranean countries due to its phytotoxicity [1]. Virgin olive oil is extracted from olive fruit using mechanical processes including the crushing of the olive fruits followed by malaxation step which prepares the resulting paste for subsequent separation of the oil. The oily phase is separated through pressure or centrifugation. The main olive oil extraction method used in many Mediterranean countries is the continuous centrifugation system known as three-phase system [2]. The centrifugal decanter allows for the separation of three phases; the olive oil, wastewater (OMW) and pomace (solid waste). Beside the traditional press extraction system and the two-phase centrifugal system, the olive oil extraction processes produce between 10 million [3] and 30 million m^3^ per year of OMW [4].

Moreover, there are three main trade preparations of table olives: a) the green Spanish style, b) Californian style (ripe olives by alkaline oxidation) and c) Greek style (naturally black olives) [5,6]. As mentioned, table olive preparation generates large volumes of wastewaters. Both effluents, OMW and table-olive wastewaters (TOW), could be grouped under a single name known as “olive processing wastewaters, OPW”. The disposal of these wastewaters without prior treatment has led to severe problems for the whole ecosystem. OMW and table-olive brines have showed toxicity to some plants and microorganisms since they exhibit a substantial concentration of polyphenols up to 10 g/L [7]. The phytotoxic and antibacterial properties of OMW polyphenols were demonstrated in a relatively recent study along with the negative effects they have on increasing the salinity and acidity of soils [8].

Olive oil contains only 2% of the total phenolic content from the olive fruit and the remaining 98% is lost in OMW and pomace [9]. Furthermore, during the processing of table olives, hydrolyzed polyphenols are liberated into the brines. Thus, OPW are potentially a rich source of a diverse range of polyphenols with a large spectrum of biological activities. Polyphenols are found widely in a variety of plants including olives and are involved in many vital functions including defense [10].

According to several studies, polyphenols from olive also represent natural anti-inflammatory agents [11] and exhibit a wide range of interesting bioactivities such as antimicrobial, antiatherogenic, antitumoral, cytoprotective and cardioprotective properties [12,13]. Thus, they may have significant health benefits. They could therefore be used to replace synthetic drugs which can cause side effects.

Studies have also demonstrated that phenolic compounds exhibited broad spectrum antibacterial activity [14]. Phenolic content of OMW has been demonstrated to have a molluscicidal activity [15] in addition to antimicrobial activity [3]. Most studies of antimicrobial activity have focused on ecological and environmental consequences [16] or on agricultural applications [17].

A large number of research papers have been published regarding the chemical composition of olives and olive oil; but, only few studies have focused on isolating and identifying compounds from the OPW [18–20]. The recovery of these bioactive metabolites, especially hydroxytyrosol, aromatic acids, and conjugated aromatic acids from OPW, is of particular interest since they possess very promising bioactivities and health promoting properties [12].

As part of a comprehensive study of the nature and functionality of OPW phenolic extracts, we investigated the antioxidant and antibacterial activity of OMW samples generated by two different olive oil processing techniques, and TOW samples issued from brines of table olives with different stages of maturity. The antioxidant and antibacterial activity was studied using the DPPH and TLC-bioautography assay, respectively.

## Materials and methods

### Olive processing wastewater samples

Tensift Hydraulic Bassin Agency is the authority who is responsible for the management of water resources in Tensift region in a sustainable manner. The sampling of the Olive mill wastewater and the Table olive brines was conducted with the agreement of the olive mill and the olive manufacturing company owners. No specific permission was required from the authority to proceed with the sampling of the olive processing wastewater. On the other hand The Tensift Hydraulic Bassin Agency promotes the collaboration with Cadi Ayyad University to conduct research studies to optimize the treatment and the recovery of olive processing wastewater. Olive mill wastewater samples were collected from two different olive mills located in the Oudaya region, in the West of Marrakech city (Morocco) during the season of 2012/2013. The two mills use different milling techniques, semi-modern (OMW1) and modern (OMW2) three-phase processes. The processed olive fruits are from the Moroccan Picholine variety. Table-olive brines were kindly provided by Agro-Hind table olive manufacturing company (Marrakech, Morocco). Three different samples of table-olive wastewater were studied, green-olive brine (GTOW), black-olives brine (BTOW) and purple olive brine (PTOW). Sampling was carried out in a manner to insure a representative sample. All analyses were done at least twice.

### Physicochemical characterization of samples

The physicochemical characterization of OPW samples was carried out as follow: Chemical oxygen demand (COD) was determined by the dichromate method as used by previous study [19]. Briefly, wastewater sample was diluted up to 100 fold and introduced into a lab-prepared digestion solution containing potassium dichromate, mercuric sulfate and sulfuric acid. The mixture was incubated for 120 min at 150°C in a COD reactor (Model WTW CR3000, Germany). COD concentration was then measured colorimetrically at 600 nm using a MultiLab P5 (WTW, Germany). Standard solutions of 1, 2, 3, and 4 g of O_2_ per liter were prepared using potassium biphthalate. Total suspended solids were determined after filtration of a given volume of olive mill wastewater samples through a Whatman filter (934-AH). The dry residue (g/L) was then determined indirectly by drying the permeate at 105°C overnight. The other physicochemical parameters (i.e. pH, sugar, acidity, sodium and potassium) were analyzed as described previously by Kiai and Hafidi [21]. Water-soluble phenolic compounds of OPW samples were extracted using solvent-solvent extraction three times first with equal volume of ethyl acetate followed by half volume of hexane. After evaporation of the organic phase using a vacuum rotary evaporator, the residue was dissolved in pure methanol and kept at 4°C until use [22].

### Total phenolic content, flavonoids, flavanols, and proanthocyanidins determination

The total phenolic concentration (TPC) in methanolic extracts recovered after extraction with ethyl acetate was determined by the Folin–Ciocalteau colorimetric method [23]. For total flavonoids, a modified method of Kim *et al*. [24] was used. Briefly, 0.2 mL aliquot of extract was mixed with 0.8 mL of distilled water in a 5 mL assay tube followed by 60 μL of 5% NaNO_2._ The mixture was allowed to react for 5 min. Following this, 40 μL of 10% AlCl_3_ was added and the mixture was left for a further 5 min before adding 0.4 mL of 1M Na_2_CO_3_ and 0.5 mL of distilled water to the reaction mixture. The absorbance was measured at 510 nm against a blank prepared similarly except that distilled water was used instead of extract. Total flavonoids content was calculated from a calibration curve using catechin as a standard, and expressed as mg catechin equivalents per liter of the extract (CTE/L).

Flavanols were determined after derivatization with *p*-(dimethylamino)-cinnamaldehyde (DMACA), using the optimized protocol established by Nigel and Glories [25]. Extract (0.2 mL), suitably diluted with methanol, was introduced into a 5 mL assay tube and 0.5 mL HCl (0.24 M in methanol) and 0.5 mL DMACA solution (0.2% in methanol) were added. The mixture was allowed to react at room temperature for 5 min, and the absorbance was measured at 640 nm. Control was prepared by replacing sample with methanol. The concentration of total flavanols was calculated from the calibration curve obtained using catechin as a standard. The results were expressed as mg of catechin equivalents per liter of the extract (CTE/L). Proanthocyanidins were analyzed by the method described by Waterman and Mole [26]. Butanol reagent was prepared by mixing 70 mg ferrous sulfate (FeSO_4_) with 5 mL concentrated HCl and made up to 100 mL with *n-*butanol. An aliquot of 0.1 mL sample was mixed vigorously with butanol reagent (1.4 mL) and heated for 45 min at 95°C water bath. The sample was than cooled and 0.5 mL *n-*butanol was added to it, before the absorbance was read at 550 nm was measured. Results were expressed as cyaniding equivalents per liter of the extract (CYE/L) using a molar extinction coefficient ε = 26,900 with a molecular weight of 449.2.

### Antibacterial activity

#### Disc diffusion assay

Antibacterial activity was tested against a panel of non pathogenic microorganisms listed below: *Lactococcus lactis* (HP), *Lactobacillus bulgaricus* (ATCC11842), *Staphylococcus aureus* (DPC5246), *Bacillus subtilis*, *Listeria innocua* (WIT 361), *Escherichia coli* (DSMZ 10720) and *Salmonella tryphimurium* (LT2). All cultures were obtained from the Pharmaceutical and Molecular Biotechnology Research Centre (PMBRC) at Waterford Institute of Technology (Ireland). The disc diffusion method, known as the Kirby-Bauer method, was used to determine the antibacterial activities of OPW phenolic extracts. A volume of 5 mL of BHI (Brain Heart Infusion) broth with 50 μL of *S*. *aureus*, *B*. *subtilis*, *L*. *innocua*, *S*. *typhimurium* and *E*. *coli* bacteria was incubated at 37°C overnight. *B*. *subtilis* was incubated at 34°C while shaking at 200 rpm. M17 medium with 0.5% lactose was used for *L*. *innocua* incubated at 37°C and MRS broth (agar of Man, Rogosa and Sharpe) was used for *L*. *bulgaricus* and incubation at 37°C under anaerobic conditions. 1 mL of each culture was centrifuged at 13,000 rpm for 2 min. The supernatant was removed and the pellet was re-suspended in 1 mL of sterile maximum recovery diluent (MRD) vortexed for ~ 30 s and then re-centrifuged at 13,000 rpm for 2 min. The supernatant was removed for a second time and the cell pellet re-suspended in 1 mL of sterile MRD and vortexed for ~ 30 s. The OPW phenolic extracts were dried by nitrogen flux, weighed and reconstituted in pure methanol to give a final concentration of 100 mg/mL. Blank discs, 6 mm were allowed to warm to room temperature after removal from a– 20°C freezer for 1h and then impregnated with 10 μL of the phenolic extract solution to have a 1 mg extract/disc of each sample. The discs were then left to dry for at least 20 min under sterile conditions to allow evaporation of the solvent. The negative control (10 μL of methanol) was prepared in a similar manner to the phenolic extract discs. The positive control was chloramphenicol (10 μg) antibiotic discs.

#### TLC plates

The phenolic compounds in the tested extracts were separated on aluminum-backed thin layer chromatography (TLC) plates with different solvent systems widely used in chromatography. The TLC plates were developed under saturated conditions with each of the eluent systems. The developed plates were then dried to remove traces of solvent on the plates.

#### TLC-bioautography

The bioautography method allows both separation and microbiological detection on the same plate. In the current study, two bioautographic assays were used. The TLC separation was developed, optimized and fully validated using *S*. *aureus* as an indicator bacteria to detect antibacterial activity. The first assay was based on the overlay method in which a bacterial seeded agar medium was applied on the TLC plate. The second assay was based on spraying the chromatograms with bacterial suspension until a layer was formed homogenously on the surface. This process was carried out in the laminar flow cabinet under sterile conditions. Thereafter, the plates were incubated overnight at 37°C in the dark. The produced inhibition zone was visualized by spraying the plate with a 2.5 mg/mL solution of MTT dye (3-(4,5-dimethylthiazol-2-yl)-2,5-diphenyltetrazolium bromide) and further incubated overnight. Clear bands indicated bacterial inhibition as the MTT is not reduced to the red color formazan. Live bacterial cells will convert the MTT into red color formazan.

### Antioxidant activity

#### Free radical scavenging activity

The antioxidant activity of the extracts was evaluated based on hydrogen-donating or radical-scavenging ability using the stable free radical 2,2-diphenyl-1-picrylhydrazyl (DPPH). An amount (0.1 mL) of phenolic extracts was added to 3 mL of 0.04% methanolic solution of DPPH [18]. The mixture was mixed thoroughly and incubated in dark at room temperature for 60 min. The decrease in absorbance was then measured at 517 nm, against methanol as a blank. The capacity of the tested samples to scavenge the DPPH radical was calculated as a percentage of DPPH discoloration using the following equation:

$$\%\;\mathrm{Inhibition}=\{1-{(A}_{\mathrm{sample}}{/A}_{\mathrm{control}})\}\times 100$$ Eq (1)

Where A_control_ was measured as the absorbance of DPPH without sample. The extract concentration providing 50% inhibition (IC_50_) was determined from the graph of percentage of inhibition against phenolic extract concentration [19].

#### TLC bioautography assay with DPPH reagent

In order to screen the antioxidant activity of the tested phenolic extracts, a TLC bioautography method was performed [27]. After separation on TLC plates, the compounds with free radical scavenging activity were determined *in situ* with DPPH reagent [27]. The TLC plate was observed under visible light. Areas producing yellowish bands against the purple background were considered as antioxidants.

### HPLC-MS analysis of phenolic extracts

HPLC analysis was performed on an Agilent 1200 series equipped with an 210 Agilent 1200 series binary pump SL, an Agilent 1200 series G1316B SL 211 temperature-controlled column oven, a micro vacuum degasser and a photodiode 212 array (PDA) detector, controlled by EZChrom software. Separation was achieved using Waters symmetry C18 5 μm, 3.9 x 150 mm column with a gradient run using A = 0.1% aqueous formic acid, B = 89.5:9.5:1 Methanol/nitric acid/formic acid. The gradient run starts with 10% B for 10 min, 30% B until 20 min, maintaining 30% B until 25 min, 40% B until 45 min, then until 50 min at 50% B, until 60 min at 100% B, until 65 min at 10% B and finally until 75 min at 10% B, making the total run time of 75 min. The used flow rate was 0.2 mL/min and 10 μL injection volume. The extracts were analyzed at 240, 280 and 365 nm. Quantitative determinations were carried out using external standards. HPLC analysis was performed first on the standards, followed by the OPW extracts, and finally spiking the samples with the standards. The identification of phenolic compounds was confirmed using LC-MS analysis. LC-MS conditions were same as HPLC conditions using negative mode, scan between m/z 15–500 and target ion at m/z 153 (hydroxytyrosol), m/z 137 (tyrosol), at m/z 540 (oleuropein), m/z 164 (*p*-coumaric acid), and m/z 193 (ferulic acid).

## Results and discussion

### Physicochemical characterization of olive processing wastewaters

Table 1 shows the results from the analysis of common physicochemical parameters and chemical analysis of the five wastewaters from the olive processing industry.

**Table 1:** Physicochemical characterization of olive mill wastewater and table-olive brine samples.

Parameters	Unit	Olive mill wastewater		Table-olives wastewater		
		OMW1	OMW2	GTOW	PTOW	BTOW
pH	-	5 ± 0.10	5.10 ± 0.10	4.5 ± 0.1	4.5 ± 0.1	5.1 ± 0.1
EC	mS/cm	56.30 ± 0.50	11.01 ± 0.60	76.2 ± 0.4	83.8 ± 0.6	106.4 ± 0.5
Acidity	g/L	-	-	6.6 ± 0.63	5.81 ± 0.63	-
Color	-	-	-	0.75 ± 0.01	0.8 ± 0.05	20.6 ± 1.1
TPC	g TYE/L	8.5 ± 0.4	6.46 ± 0.8	3.67 ± 0.4	4.5 ± 0.1	2.6 ± 0.1
Sugar	g/L	-	-	1.6 ± 0.12	6.9 ± 1.53	8.5 ± 1.8
COD	g of O_2_/L	110 ± 4.9	50 ± 5.4	3.26 ± 0.1	3.12 ± 0.15	12.6 ± 3.33
Dry residue	g/L	194.2 ± 11	132.7 ± 7	84.7 ± 2.05	101.5 ± 1	275.4 ± 4.8
TSS	g/L	86 ± 5	50 ± 3.5	1.68 ± 0.11	1.69 ± 0.02	2.6 ± 0.26
Sodium	g/L	2.10 ± 0.10	1.45 ± 0.09	27.5 ± 1.5	26.2 ± 1	9.63 ± 0.5
Potassium	g/L	1.24 ± 0.12	0.55 ± 0.02	1.3 ± 0.3	2.9 ± 0.5	22 ± 1.2

EC: electrical conductivity, TPC: total phenolic content, COD: chemical oxygen demand, TSS: total suspended solids, OMW1: semi-modern OMW phenolic extract, OMW2: modern OMW phenolic extract, GTOW: green table-olive wastewater, POTW: purple table-olive wastewater, BTOW: black table-olive wastewater.

OMW are relatively dense and acidic with a high organic load that reaches values as high as 110 g/L COD (Table 1). It contains large amounts of dry residue up to about 194 g/L. OMW1, which is from a semi-modern three-phase process, shows high salinity and therefore higher electrical conductivity (EC) compared to OMW2. This is due to the fact that some producers use salt for conservation of olives until milling. OMW2 was found to have lower physicochemical values than OMW1 (Table 1) which could be attributed to the relatively larger volumes of water used in modern olive milling processes [19], resulting in relatively diluted OMW. The BTOW exhibits a dark color and had the highest organic content but the lowest phenolic content (Table 1). Amongst other organic constituents, OPW (OMW and TOW) contain high concentration of phenolic compounds ranging from 2.6 to 8.5 g/L, and high EC which exceeds the environmental legislation limit, set at 0.5 mg/L for phenolic compounds [28] and 3 mS/cm for EC [29].

### Antibacterial activity

#### Disc diffusion assay

No antibacterial activity was observed for negative controls whilst the zone of inhibition for positive controls was between 15.0 ± 0.5 mm and 23.3 ± 2.5 mm for *S*. *aureus* and *L*. *bulgaricus*, respectively. All phenolic extracts tested were active against *S*. *aureus*. None of the tested samples were active against *L*. *innocua*. OMW1 extract (semi-modern process) was active against all tested bacteria except *L*. *innocua* while OMW2 was only active against *S*. *aureus*, *B*. *subtilis* and *L*. *bulgaricus*. OMW1 demonstrated the highest antibacterial activities compared to other phenolic extracts. This result can be attributed partially to the fact that OMW1 exhibits the highest concentration of phenolic compounds (Table 1).

In order to study the effect of concentration of phenolic extracts on their antibacterial activity (against *S*. *aureus*), different concentrations of OMW phenolic extracts were tested for their antibacterial activity and compared to TOW phenolic extracts (Table 2). The antibacterial activity increased with the increase in the phenolic content of the extract. The green brine phenolic extract (GTOW) demonstrated the highest antibacterial activity compared to the other TOW phenolic extracts (BTOW and PTOW).

**Table 2:** Effect of concentration of OMW and TOW phenolic extracts on their antibacterial activity against *S*. *aureus* using the disc diffusion assay.

Test substance (amount/disc)	Zone of Inhibition (mm)
**OMW1 (0.125 mg)**	6 ± 0.5
**OMW1 (0.5 mg)**	7 ± 0.5
**OMW1 (1.25 mg)**	8 ± 0.5
**OMW1 (5 mg)**	11 ± 0.5
**PC**	15.6 ± 0.6
**NC**	0
**OMW2 (0.125 mg)**	6 ± 0.5
**OMW2 (0.5 mg)**	7 ± 0.5
**OMW2 (1.25 mg)**	8 ± 0.5
**OMW2 (5 mg)**	9.3 ± 0.6
**PC**	16 ± 0.5
**NC**	0
**PTOW (5 mg)**	10 ± 1
**BTOW (5 mg)**	9 ± 0.5
**GTOW (5 mg)**	12 ± 1
**PC**	15 ± 1
**NC**	0
**GTOW (0.25 mg)**	6 ± 0.5
**GTOW (0.5 mg)**	8 ± 0.5
**GTOW (1.25 mg)**	9 ± 0.5
**GTOW (2.5 mg)**	11 ± 0.5
**PC**	14.6 ± 0.6
**NC**	0

PC: positive control; NC: negative control; OMW1: semi modern OMW phenolic extract; OMW2: modern OMW phenolic extract; GTOW: green brine phenolic extract; BTOW: black brine phenolic extract; PTOW: purple phenolic extract.

At concentrations of 0.5, 1.25 and 5 mg the GTOW extract had higher antibacterial activity against *S*. *aureus* compared to the OMW extracts. These results demonstrated that the antibacterial activity of the phenolic extracts is not only correlated to their concentration but the key factor governing their antibacterial activity is their phenolic profile. During the fermentation of table olives, the diffusion of phenolic compounds into the brine depends on several parameters such as cultivar characteristics, fruit skin permeability, type of phenolic compounds present in olive flesh and their ability to diffuse out of the fruit [21].

In a similar study [30], hydroxytyrosol and oleuropein were found to be cytotoxic to many clinical bacterial strains although to a lesser extent than the ATCC strains. The authors reported that the minimum inhibitory concentration of hydroxytyrosol and oleuropein against *S*. *aureus* was 3.9–31.25 mg/L and 62.5–125 mg/L, respectively. In a relatively recent study, hydroxytyrosol, tyrosol and oleuropein did not show any bactericidal activity at a concentration as high as (20 mM) 0.3 g/L, 0.27 g/L and 1 g/L, respectively [31]. These results contribute to the debate about the antimicrobial activity of these olive compounds, although the differences in experimental conditions and microorganisms used to test the efficacy of these antimicrobials make it difficult to compare their effectiveness.

#### Thin layer chromatography (TLC) separation

Prior to the bioautography assay and in order to separate the active components present in the crude phenolic extracts, TLC was undertaken and the most efficient solvent system was determined. Nine mobile phase systems containing solvents of different polarity (hexane: acetone (4/6); chloroform: methanol (varying ratios) and other solvent systems to include combinations of these solvents and ethyl acetate and/or dichlorobenzene were used to determine the appropriate solvent system for separation of compounds in a particular extract by TLC.

The Rf (retardation factor) value which is the ratio of movement of the solute from its origin to the movement of the solvent from origin determines the separation of compounds on the solid phase of TLC plate. A ratio of 4:6, *v/v* hexane/acetone phase was found to be the best chromatographic system for achieving separation of phenolic compounds from a mixture based on the observed spots number and the shape of the spots (round and distinct from each other).

#### TLC-bioautography

Two TLC-bioautographic assay methods were developed, optimized and fully validated in our laboratory using *S*. *aureus* as an indicator bacterium to detect antibacterial activity. The first method was agar-overlay or immersion bioautography, where the developed plate was overlaid with 15 mL of molten agar seeded with 150 μL of bacterial culture. After overnight incubation, the inhibition zone was visualized by spraying with MTT dye. The bioactive compounds were transferred by diffusion from the stationary phase to the agar layer containing the bacteria. However, the use of agar gel as a support medium has some disadvantages including slow diffusion and bad contrast. In order to optimize the visualization of the inhibition bands, a second method was used using TLC plates sprayed with an overnight culture of *S*. *aureus* (50 μL in 5 mL of BHI). Hence, a thinner layer of agar was covering the TLC plate, allowing minimum diffusion.

The bioautography screening of OMW and brine extracts showed positive activity against *S*. *aureus*. Bacterial inhibition was denoted by clear spot against a red purple background on the TLC plate after spraying with MTT. Most of the compounds separated on the TLC plates were active against *S*. *aureus*.

The bioautography test confirmed the results of the disc diffusion assay demonstrating concentration dependent antibacterial activity. The highest activity was registered by GTOW phenolic sample compared to the other OPW phenolic samples (Table 2).

When all of the compounds that showed an activity based on bioautography were isolated and characterized they often had a much lower activity than expected (data not shown) indicating synergism could be playing a significant role [32]. Compounds found in OMW that were reported to exhibit antibacterial activity are tyrosol, oleuropein, hydroxytyrosol, 4-hydroxybenzoic acid, vanillic acid, and *p-*coumaric acid [33]. It has been reported that olive polyphenols such as hydroxytyrosol have *in vitro* antibacterial activity against both Gram-negative and Gram-positive bacteria responsible for intestinal tract and respiratory tract infections [34].

### Antioxidant activity

#### DPPH scavenging activity

DPPH is a commonly used substrate (free radical) for fast and easy evaluation of the antioxidant activity due to its stability, reliability and the simplicity of the assay [35]. Compounds with antioxidant properties would change the purple color of DPPH to yellow as the radical is quenched by the antioxidant [35]. The color change can be measured quantitatively by spectrophotometric absorbance at 517 nm. Caffeic acid was used as the reference antioxidant compound. Caffeic acid (3,4-dihydroxycinnamic acid) is one of the major hydroxycinnamic acids present in OMW and has been identified as one of the main antioxidants in this study. The IC_50_ of caffeic acid was found to be 121 ± 6 μg/mL. OMW1 and OMW2 exhibited highly interesting DPPH radical scavenging ability compared to the phenolic extracts of different brines (Table 3). DPPH radical was less sensitive to the concentration variation of brine extracts compared to the case of OMW extracts (Table 3). This difference in activity is mainly due to the phenolic composition of each wastewater type. OMW1 showed the highest antioxidant activity (the lowest IC_50,_ Table 3) followed by OMW2. Both OMW extracts showed considerably higher antioxidant activity compared to caffeic acid. PTOW phenolic extract showed an IC_50_ comparable to caffeic acid, followed by GTOW and finally the BTOW which showed the lowest radical scavenging activity (Table 3).

**Table 3:** Total phenolic content, phenolic constituents and antioxidant activity (IC_50_) of different olive processing wastewaters samples. The values in the brackets are the proportions to the total phenolic content, TPC.

Sample	TPC	Flavonoids	Flavanols	Proanthocyanidins	IC_50_
	TYE g/L	CAE g/L	CAE mg/L	CYE mg/L	mg/L
OMW1	8.5 ± 0.4	5.74 ± 0.36	2.63 ± 0.12	19.32 ± 2.03	15.83 ± 1.9
	(100%)	(67.53%)	(0.031%)	(0.227%)	
OMW2	6.46 ± 0.8	2.85 ± 0.24	2.1 ± 0.09	14.10 ± 1.53	32.32 ± 4.7
	(100%)	(44.11%)	(0.032%))	(0.218%)	
GTOW	3.67 ± 0.04	0.87 ± 0.05	0.23 ± 0.02	42.57 ± 0.71	173 ± 2.8
	(100%)	(23.71%)	(0.006%))	(1.159%)	
PTOW	4.5 ± 0.01	1.22 ± 0.09	0.17 ± 0.03	533.39 ± 20.51	126.3 ± 3.9
	(100%)	(27.11%)	(0.004%)	(11.85%)	
BTOW	2.6 ± 0.01	0.67 ± 0.05	4.17 ± 0.14	399.83 ± 40.31	261.3 ± 4.8
	(100%)	(25.78%)	(0.160%)	(15.38%)	

The different reactions of the phenolic extracts against DPPH radical depend on the nature of the antioxidants involved in the reaction. Such behavior must be the result of the different individual contributions of the phenolic compounds present in the samples. Furthermore, in a previous work, we have found that the antioxidant capacity of different OMW extracts was directly correlated to the percentage of free hydroxytyrosol and their antioxidant activity was found to be the result of their phenolic profile (composition) rather than their phenolic concentration [19]. Hydroxytyrosol is found in nature (in olive fruit, olive oil and olive leaf) in the form of its elenolic acid ester: oleuropein or in the form of hydroxytyrosyl acetate. Owing to the chemical or enzymatic hydrolysis which occurs during storage, free hydroxytyrosol is liberated progressively against time [15].

The antioxidant activity of OPW and especially OMW varies widely from study to study. The variation of the OMW phenolic content and its antioxidant properties are affected by many factors such as olive cultivar, the olive oil extraction process, the physicochemical characteristics of OMW samples, the fungal and bacterial flora existing in OMW, and finally the storage conditions.

Previous studies on the degradation of polyphenols in its original OMW matrix during the extraction process and upon storage revealed their poor stability due to the complex and reactive nature of OMW, where oxidation, condensation, polymerization, and enzymatic hydrolysis can all potentially take place [36,37]. It has been reported previously that OMW from semi-modern three-phase process has higher phenolic content than OMW from modern three-phase process [19]. This study confirms these findings and highlights the effect of milling process on the phenolic content and composition of the generated OMW.

Antioxidant compounds are reducing agents which exhibits its antioxidant properties through scavenging free radicals. Therefore, they are able to extend shelf life of food and pharmaceutical products by decreasing the oxidation rate of the products, hence preventing deterioration of the products during processing and storage. He *et al*. [38] investigated the antioxidant capacity and stability of bioactive compounds in purified olive extract (POE) prepared from OMW by adsorption onto a polymer resin. Their results showed that air/oxygen was the main factor that affects the stability of POE during storage at low temperature, whereas an increase in temperature significantly decreased the total phenolic content of the extract (20–24% reduction) [38].

Many studies have investigated the phenolic content of OMW; however, the phenolic composition of brines is also a very promising low-cost source for high-added value bioactive compounds, especially hydroxytyrosol. To the best of our knowledge, this is the first study to compare the phenolic profile and antioxidant property of olive mill effluents and table-olive wastewaters.

#### Bioautography assay

**Fig 1:**
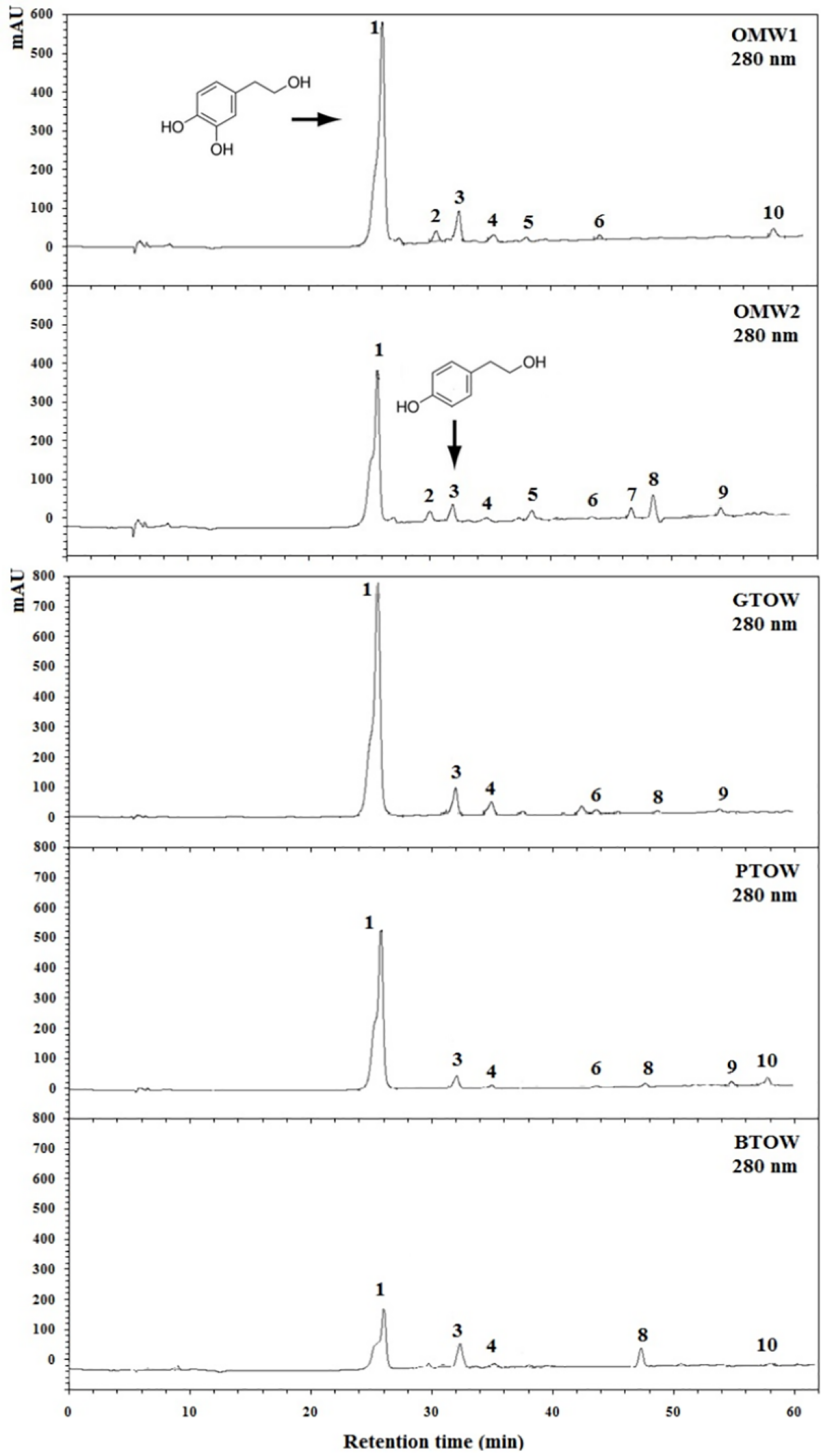
HPLC chromatograms of OMW phenolic extracts. 1. Hydroxytyrosol, 2. 3,4-dihydroxyphenylacetic acid, 3. Tyrosol. 4. Protocatechuic acid derivative, 5. Caffeic acid. 6. *p*-coumaric acid. 7. Ferulic acid derivative, 8. Ferulic acid, 9. Luteolin derivative, 10. Oleuropein. Peaks 1, 3, 6, 8 and 10 were identified by use of standards. The remaining peaks were tentatively identified by comparison with literature data. (OMW1: olive mill wastewater from semi-modern process, OMW2: olive mill wastewater from modern process, GTOW: green table-olive brine, PTOW: purple table-olive brine and BTOW: black table-olive brine).

Bioautography is also a useful method for separation and detection of the active antioxidants in a mixture of compounds [39]. The TLC bioautography assay is the method of choice in the screening of antioxidants due to advantages such as its simplicity, flexibility and high throughput. On the TLC, antioxidant compounds would be seen as a white/yellow spots on a purple background [27]. Fig 1 shows a profile of brine and OMW phenolic extract at different concentration presenting antioxidant activity compared to standard phenolic compounds. Hydroxytyrosol is the most efficient antioxidant agent present in OPW extracts as shown by Fig 1, even at a low concentration of the extract (1 mg/L for GTOW). The absence of antioxidant activity in some bands could be due to the evaporation of the active compounds (highly volatile compounds), photo-oxidation or due to the low quantity of the active compound on the TLC plate [40].

The different detection sensitivities observed in Fig 1 are attributed to the diverse nature of the oxidative compounds. The phenolic extract separated with 4:6, v/v hexane:acetone showed several TLC bands with strong antioxidant activity which increases with the increasing concentration of the phenolic extract. Results reported in Fig 1 suggested that hydroxytyrosol (HT), *p*-coumaric acid (PC) and caffeic acid (CA) may be present in OMW and brine phenolic extracts as visualized by the DPPH-TLC bands. The presence of these compounds in confirmed with LC-MS.

### Phenolic constituents of the olive processing wastewaters extracts

Flavonoids, the most diverse and the largest group of natural phenolic compounds, are known to have antioxidant, antiallergic, antimicrobial, anti-inflammatory, and anticarcinogenic properties [41]. This phenolic group constitutes the largest part of OMW1 phenolic content (67.53%). However, flavonoids correspond only to 44.11% of OMW2 phenolic content (Table 3). The three studied table-olive brines contained between 23% and 27% flavonoids of their total phenolic content. Besides their antioxidant activities, flavonoids were found to inhibit lipid peroxidation, platelet aggregation and possess chemopreventive effects on carcinogenesis [42]. The main flavonoid subclasses in OMW are proanthocyanidins and flavanols [19]. These phenolics were found to decrease the risk of ischemia-reperfusion damage of heart in rat by increasing plasma antioxidant activity [43]. In this study, for the OPW samples, flavanols and proanthocyanidins were found to have a lower proportion, of approximately 2% of their total phenolic content, except for BTOW which exhibits the highest proportion of proanthocyanidins (15.38%) followed by PTOW (11.85%) (Table 3). Furthermore, the flavanols content varied from 0.17 to 4.17 CAE mg/L (0.004 to 0.16%) of the total phenolic content of different OPW samples.

### HPLC-MS analysis of olive processing wastewaters

**Fig 2:**
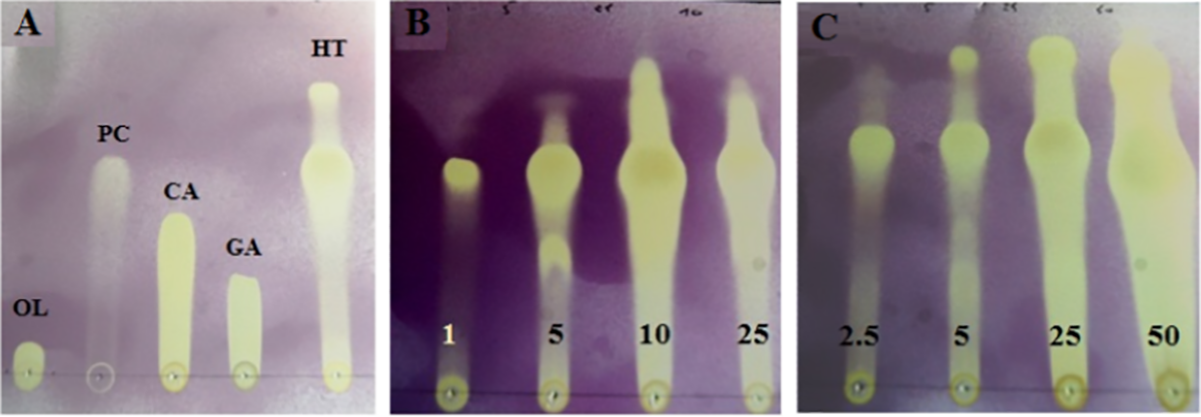
Developed TLC bioautography plates (4:6, v/v Hexane: Acetone) stained with 2.54 mM DPPH solution visualized under visible light. **A**. standard phenolic compounds at 1 mg/mL–OL: oleuropein; PC: *p*-coumaric acid; CA: caffeic acid; GA: gallic acid; HT: hydroxytyrosol. **B**. GTOW phenolic extract at 1, 5, 10 and 25 mg/mL. **C**. OMW1 phenolic extract at 2.5, 5, 25 and 50 mg/mL.

HPLC-MS analysis was performed to determine, compare, identify and quantify the phenolic compounds in OPW samples (Fig 2 and Table 4). Compounds from three main phenolic groups were identified, simple phenols (hydroxytyrosol and tyrosol), phenolic acids (*p*-coumaric acid, ferulic acid and its derivative) and secoiridoids (oleuropein).

**Table 4:** Summary results of the possible phenolic compounds from different olive processing wastewaters as identified by HPLC analysis and their respective concentrations (in mg/L).

Suspected compound	Olive processing wastewaters samples				
	OMW1	OMW2	GTOW	PTOW	BTOW
Hydroxytyrosol	52.8	34.32	83.6	48.6	10.9
Tyrosol	6.88	3.48	8.7	3.24	6.46
*p*-coumaric acid	0.16	2.72	n.d	0.32	0.24
Ferulic acid derivative	n.d	5.72	n.d	n.d	0.02
Ferulic acid	n.d	0.48	n.d	n.d	0.06
Oleuropein	31.36	7.4	0.66	48.8	10.82

n.d: not detected.

The total phenolic concentration as revealed by the HPLC-MS quantification is not corresponding to the depicted spectrophotometric estimation in Table 3. Previous studies highlighted that the disadvantage of the Folin-Ciocalteu colorimetric method is that come reducing agents may be present in the extract can interfere in the analysis and consequently overestimate the total phenolic content of the extract [44]. From the current study, hydroxytyrosol and tyrosol were the two predominant phenolic compounds in the OMW samples, as shown by HPLC chromatograms. Hydroxytyrosol has been an important focus of research since its discovery due to its bioactivity on inhibition of human LDL oxidation [45], prevention of platelet aggregation and its anti-inflammatory [46] and anticancer properties [47].

OMW1 and OMW2 showed different phenolic profiles in terms of concentration and also in composition since oleuropein was detected only in OMW1 and ferulic acid and luteolin derivatives were detected only in OMW2 (Fig 2). The difference in antimicrobial and antioxidant activities–as shown previously–can be related in part to the difference of phenolic profile of each sample. Hydroxytyrosol was found to be the major phenolic compound present in both effluent types. This compound has very high commercial value and can turn the olive processing wastewaters from wastewater to resource for high-added value compounds. Recently, hydroxytyrosol has become commercially available for research but it is expensive and not available in large quantities for use at an industrial scale. Some other polyphenols reported to be present in found in OPW possess bactericidal activity in their original concentration, and can be used as pesticides in agriculture for the protection of olive trees or other crops [48,49].

## Conclusion

In this study, TLC and TLC-DPPH bioautography was used for the first time to assess the antioxidant and antibacterial activities of OMW and olive brine. Considerable differences between samples were observed in terms of antibacterial and antioxidant activity. The observed differences in antioxidant activities can be attributed in part to the difference of phenolic profile of each sample. Since these compounds act at very low concentrations, a small difference in the phenolic profile between samples can affect considerably its antioxidant and antibacterial activities. This is evident from the poor correlation between antibacterial and antioxidant properties observed in this study. Several studies have reported previously that antibacterial activity is mainly related to non-polar compounds and antioxidant activity to polar compounds [50,51]. OMW and TOW are a promising source for natural high-added-value compounds. Further studies are needed for the isolation and characterization of the OPW phenolic fractions to elucidate their different antioxidant and antibacterial mechanisms and the existence of possible synergism.

## Supporting information

S1 Fig Photographs of the developed TLC bioautography plates (4:6, v/v Hexane: Acetone). (a) overlaid with seeded molten agar (10 μL of OMW1 at 100 mg/mL) and (b) sprayed with an overnight culture of S. aureus (10 μL of OMW1 at 10 mg/l and 10 μL of GTOW at 50 mg/mL). OMW1: olive mill wastewater from a semi-modern process, GTOW: green table-olive wastewater phenolic extract.
(PDF)

S1 File HPLC conditions. (PDF)

S2 File LC-MS conditions. (PDF)

S3 File HPLC and MS chromatogram of OMW samples. (PDF)

S1 Table Antibacterial activity of OMW phenolic extracts (1mg/disc). Diameter of zone of inhibition (mm) including diameter of 6 mm disc; Results quoted as the average of a minimum of six measurements ± standard deviation; “-”indicates no visible zone of inhibition. PC: positive control; NC: negative control; OMW1: phenolic extract of OMW from semi-modern three-phase process; OMW2: phenolic extract of OMW from modern three-phase process.
(PDF)

S2 Table TLC data of OMW1 phenolic extract using different solvent ratios. (PDF)

S3 Table Summary results of the possible polyphenols from different olive processing wastewaters extracts as identified by HPLC analysis. (PDF)

## References

[1] Hanafi F, Belaoufi A, Mountadar M, Assobhei O. Augmentation of biodegradability of olive mill wastewater by electrochemical pre-treatment: Effect on phytotoxicity and operating cost. J. Hazard. Mater. 2011;190: 94–99. doi: 10.1016/j.jhazmat.2011.02.087 21435785 10.1016/j.jhazmat.2011.02.087

[2] Souilem S, El-Abbassi A, Kiai H, Hafidi A, Sayadi S, Galanakis CM. Olive oil production sector: environmental effects and sustainability challenges. Olive Mill Waste. Elsevier. 2017: 1–28. doi: 10.1016/B978-0-12-805314-0.00001–7

[3] Niaounakis M, Halvadakis CP. Olive processing waste management. literature review and patent survey, Elsevier 2006.

[4] Sabbah I, Marsook T, Basheer S. The effect of pretreatment on anaerobic activity of olive mill wastewater using batch and continuous systems. Process Biochem. 2004;39: 1947–1951. doi: 10.1016/j.procbio.2003.09.026

[5] Arroyo-López FN, Querol A, Bautista-Gallego J, Garrido-Fernández A. Role of yeasts in table olive production. Int. J. Food Microbiol. 2008;128: 189–196. doi: 10.1016/j.ijfoodmicro.2008.08.018 18835502 10.1016/j.ijfoodmicro.2008.08.018

[6] Tuna S, Akpınar-Bayızıt A. The use of β-glucosidase enzyme in black table olives fermentation. Not. Bot. Horti Agrobot. Cluj-Napoca. 2009;37: 182–189.

[7] Mantzavinos D, Kalogerakis N. Treatment of olive mill effluents Part I. Organic matter degradation by chemical and biological processes: an overview, Environ. Int. 2005;31: 289–95. doi: 10.1016/j.envint.2004.10.005 15661297 10.1016/j.envint.2004.10.005

[8] Hanifi S, El Hadrami I. Olive Mill Wastewasters fractioned soil-application for safe agronomic reuse in date palm (Phoenix dactylifera L.) fertilization, J. Agron. 2008;7: 63–69.

[9] Rodis PS, Karathanos VT, Mantzavinou A. Partitioning of olive oil antioxidants between oil and water phases. J. Agric. Food Chem. 2002;50: 596–601. http://pubs.acs.org/doi/abs/10.1021/jf010864j. 11804535 10.1021/jf010864j

[10] Kusumoto D, Suzuki K. Spatial distribution and time-course of polyphenol accumulation as a defense response induced by wounding in the phloem of Chamaecyparis obtusa. New Phytol. 2003;159: 167–173. doi: 10.1046/j.1469-8137.2003.00775.x 10.1046/j.1469-8137.2003.00775.x 33873686

[11] Cicerale S, Conlan XA, Sinclair AJ, Keast RSJ. Chemistry and health of olive oil phenolics. Crit. Rev. Food Sci. Nutr. 2009;49: 218–236. doi: 10.1080/10408390701856223 19093267 10.1080/10408390701856223

[12] Zbakh H, El Abbassi A. Potential use of olive mill wastewater in the preparation of functional beverages: A review. J. Funct. Foods. 2012;4: 53–65. doi: 10.1016/j.jff.2012.01.002

[13] Lecci R, Logieco A, Leone A. The good side of pro-oxidative action of plant polyphenols: an action mechanism for pro-apoptotic activity. Proc. Bio-Olea Conf. (Extended Abstr., Corfu, Greece). 2014: 54–59.

[14] Tafesh A, Najami N, Jadoun J, Halahlih F, Riepl H, Azaizeh H. Synergistic antibacterial effects of polyphenolic compounds from olive mill wastewater. Evidence-Based Complement. Altern. Med. 2011 doi: 10.1155/2011/431021 21647315 10.1155/2011/431021 PMC3106970

[15] Obied HK, Bedgood DR, Prenzler PD, Robards K. Bioscreening of Australian olive mill waste extracts: biophenol content, antioxidant, antimicrobial and molluscicidal activities. Food Chem. Toxicol. an Int. J. Publ. Br. Ind. Biol. Res. Assoc. 2007;45: 1238–1248. doi: 10.1016/j.fct.2007.01.004 17329005 10.1016/j.fct.2007.01.004

[16] Pierantozzi P, Zampini C, Torres M, Isla MI, Verdenelli RA, Meriles JM, et al Physico-chemical and toxicological assessment of liquid wastes from olive processing-related industries. J. Sci. Food Agric. 2012;92: 216–223. doi: 10.1002/jsfa.4562 21796638 10.1002/jsfa.4562

[17] Aquilanti L, Taccari M, Bruglieri D, Osimani A, Clementi F, Comitini F, el al. Integrated biological approaches for olive mill wastewater treatment and agricultural exploitation. Int. Biodeterior. Biodegradation. 2014;88: 162–168. doi: 10.1016/j.ibiod.2013.12.010

[18] Paraskeva CA, Papadakis VG, Tsarouchi E, Kanellopoulou DG, Koutsoukos PG. Membrane processing for olive mill wastewater fractionation. Desalination. 2007;213: 218–229. doi: 10.1016/j.desal.2006.04.087

[19] El-Abbassi A, Kiai H, Hafidi A. Phenolic profile and antioxidant activities of olive mill wastewater. Food Chem. 2012;132: 406–412. doi: 10.1016/j.foodchem.2011.11.013 26434308 10.1016/j.foodchem.2011.11.013

[20] El-Abbassi A, Kiai H, Raiti J, Hafidi A. Cloud point extraction of phenolic compounds from pretreated olive mill wastewater. J. Environ. Chem. Eng. 2014;2: 1480–1486. doi: 10.1016/j.jece.2014.06.024

[21] Kiai H, Hafidi A. Chemical composition changes in four green olive cultivars during spontaneous fermentation. LWT—Food Sci. Technol. 2014;57: 663–670. doi: 10.1016/j.lwt.2014.02.011

[22] El Hadrami A, Belaqziz M, El Hassni M, Hanifi S, Abbad S, Capasso R, et al Physico-chemical Characterization and Effects of Olive Oil Mill Wastewaters Fertirrigation on the Growth of Some Mediterranean Crops. J. Agron. 2004;3: 247–254. doi: 10.3923/ja.2004.247.254

[23] Singleton VL, Orthofer R, Lamuela-Raventós RM. Analysis of total phenols and other oxidation substrates and antioxidants by means of folin-ciocalteu reagent. Methods Enzymol. 1999;299: 152–178. doi: 10.1016/S0076-6879(99)99017-1

[24] Kim DO, Chun OK, Kim YJ, Moon HY, Lee CY. Quantification of Polyphenolics and Their Antioxidant Capacity in Fresh Plums. J. Agric. Food Chem. 2003;51: 6509–6515. doi: 10.1021/jf0343074 14558771 10.1021/jf0343074

[25] Nagel CW, Glories Y. Use of a Modified Dimethylaminocinnamaldehyde Reagent for Analysis of Flavanols. Am. J. Enol. Vitic. 1991;42: 364–366.

[26] Waterman PG, Mole S. Analysis of phenolic plant metabolites Blackwell Scientific, Oxford 1994.

[27] Nickavar B, Adeli A, Nickavar A. TLC-Bioautography and GC-MS Analyses for Detection and Identification of Antioxidant Constituents of Trachyspermum copticum Essential Oil. Iran. J. Pharm. Res. IJPR. 2014;13: 127–33. 24734063 PMC3985236

[28] Pinto RTP, Lintomen L, Jr LFLL, Wolf-maciel MR. Strategies for recovering phenol from wastewater: thermodynamic evaluation and environmental concerns. Fluid Phase Equilib. 2005;228–229: 447–457. doi: 10.1016/j.fluid.2004.09.005

[29] Pescod MB. Wastewater treatment and use in agriculture Food and Agriculture Organization of the United Nations 1992.

[30] Tuck KL, Hayball PJ. Major phenolic compounds in olive oil: metabolism and health effects. J. Nutr. Biochem. 2002;13: 636–644. doi: 10.1016/S0955-2863(02)00229-2 12550060 10.1016/s0955-2863(02)00229-2

[31] Medina E, Brenes M, Garcia A, Romero C, De Castro A. Bactericidal Activity of Glutaraldehyde-like Compounds from Olive Products. J. Food Prot. 2009;72: 2611–2614. 20003748 10.4315/0362-028x-72.12.2611

[32] Eloff JN, Katerere DR, McGaw LJ. The biological activity and chemistry of the southern African Combretaceae. J. Ethnopharmacol. 2008;119: 686–699. doi: 10.1016/j.jep.2008.07.051 18805474 10.1016/j.jep.2008.07.051

[33] Soler-Rivas C, García-Rosado A, Polonia I, Junca-Blanch G, Marín FR, Wichers HJ. Microbiological effects of olive mill waste addition to substrates for Pleurotus pulmonarius cultivation. Int. Biodeterior. Biodegradation. 2006;57: 37–44. doi: 10.1016/j.ibiod.2005.10.007

[34] Granados-Principal S, Quiles JL, Ramirez-Tortosa CL, Sanchez-Rovira P, Ramirez-Tortosa MC. Hydroxytyrosol: from laboratory investigations to future clinical trials. Nutr. Rev. 2010;68: 191–206. doi: 10.1111/j.1753-4887.2010.00278.x 20416016 10.1111/j.1753-4887.2010.00278.x

[35] Ali HM, Salem MZM, Al Sahli AA. Performance of Antioxidant Activity of Methanolic Extracts from Different Parts of Some Tree Species using DPPH Radical-scavenging Assay. J. Pure Appl. Microbiol. 2013;7: 131–137.

[36] Dammak I, Neves M, Souilem S, Isoda H, Sayadi S, Nakajima M. Material Balance of Olive Components in Virgin Olive Oil Extraction Processing. Food Sci. Technol. Res. 2015,21: 193–205. doi: 10.3136/fstr.21.193

[37] Jarboui R, Sellami F, Azri C, Gharsallah N, Ammar E. Olive mill wastewater evaporation management using PCA methodCase study of natural degradation in stabilization ponds (Sfax, Tunisia). J. Hazard. Mater. 2010;176: 992–1005. doi: 10.1016/j.jhazmat.2009.11.140 20036054 10.1016/j.jhazmat.2009.11.140

[38] He J, Alister-Briggs M, De Lyster T, Jones GP. Stability and antioxidant potential of purified olive mill wastewater extracts. Food Chem. 2012;131: 1312–1321. doi: 10.1016/j.foodchem.2011.09.124

[39] Jothy S, Zuraini Z, Sasidharan S. Phytochemicals screening, DPPH free radical scavenging and xanthine oxidase inhibitiory activities of Cassia fistula seeds extract. J. Med. Plants Res. 2011;5: 1941–1947.

[40] Masoko P, Eloff JN. The diversity of antifungal compounds of six South African Terminalia species (Combretaceae) determined by bioautography. African J. Biotechnol. 2005;4: 1425–1431.

[41] Brunetti C, Di Ferdinando M, Fini A, Pollastri S, Tattini M. Flavonoids as antioxidants and developmental regulators: relative significance in plants and humans. Int. J. Mol. Sci. 2013;14: 3540–3555. doi: 10.3390/ijms14023540 23434657 10.3390/ijms14023540 PMC3588057

[42] Cirico TL, Omaye ST. Additive or synergetic effects of phenolic compounds on human low density lipoprotein oxidation. Food Chem. Toxicol. 2006;44: 510–516. doi: 10.1016/j.fct.2005.08.025 16216401 10.1016/j.fct.2005.08.025

[43] Facino RM, Carini M, Aldini G, Berti F, Rossoni G, Bombardelli E, et al Diet enriched with procyanidins enhances antioxidant activity and reduces myocardial post-ischaemic damage in rats. Life Sci. 1999;64: 627–642. 10069526 10.1016/s0024-3205(98)00605-5

[44] Sánchez-Rangel JC, Benavides J, Heredia JB, Cisneros-Zevallos L, Jacobo-Velázquez DA, Blumberg JF, et al The Folin–Ciocalteu assay revisited: improvement of its specificity for total phenolic content determination. Anal. Methods. 2013;5: 5990 doi: 10.1039/c3ay41125g

[45] Visioli F, Romani A, Mulinacci N, Zarini S, Conte D, Vincieri FF, et al Antioxidant and other biological activities of olive mill waste waters. J. Agric. Food Chem. 1999;47: 3397–3401. 10552663 10.1021/jf9900534

[46] De la Puerta R, Ruiz Gutierrez V, Hoult JR. Inhibition of leukocyte 5-lipoxygenase by phenolics from virgin olive oil. Biochem. Pharmacol. 1999;57: 445–449. 9933033 10.1016/s0006-2952(98)00320-7

[47] Owen RW, Haubner R, Würtele G, Hull E, Spiegelhalder B, Bartsch H. Olives and olive oil in cancer prevention. Eur. J. Cancer Prev. 2004;13: 319–326. 15554560 10.1097/01.cej.0000130221.19480.7e

[48] Debo A, Yangui T, Dhouib A, Ksantini M, Sayadi S. Efficacy of a hydroxytyrosol-rich preparation from olive mill wastewater for control of olive psyllid, Euphyllura olivina, infestations. Crop Prot. 2011;30: 1529–1534. doi: 10.1016/j.cropro.2011.08.006

[49] El-Abbassi A, Saadaoui N, Kiai H, Raiti J, Hafidi A. Potential applications of olive mill wastewater as biopesticide for crops protection. Sci. Total Environ. 2017;576: 10–21. doi: 10.1016/j.scitotenv.2016.10.032 27780096 10.1016/j.scitotenv.2016.10.032

[50] Hemali P, Sumitra C. Evaluation of antioxidant efficacy of different fractions of Tagetes erecta L. Flowers. J. Pharm. Biol. Sci. 2014;9: 28–37.

[51] Medini F, Fellah H, Ksouri R, Abdelly C. Total phenolic, flavonoid and tannin contents and antioxidant and antimicrobial activities of organic extracts of shoots of the plant Limonium delicatulum. J. Taibah Univ. Sci. 2014;8: 216–224. doi: 10.1016/j.jtusci.2014.01.003

