# Supplementary material for: Assessment of the antioxidant and antibacterial activities of different olive processing wastewaters
Source: PLoS One. 2017 Sep 5;12(9):e0182622. doi: 10.1371/journal.pone.0182622 (PMC5584791; doi:10.1371/journal.pone.0182622)
Supplement: S1 Fig — (a) overlaid with seeded molten agar (10 μL of OMW1 at 100 mg/mL) and (b) sprayed with an overnight culture of S. aureus (10 μL of OMW1 at 10 mg/l and 10 μL of GTOW at 50 mg/mL). OMW1: olive mill wastewater from a semi-modern process, GTOW: green table-olive wastewater phenolic extract. (PDF) [file pone.0182622.s001.pdf]

S1 Fig. Photographs of the developed TLC bioautography plates (4:6, v/v Hexane: Acetone). (a) overlaid with seeded molten agar (10 µL of OMW1 at 100 mg/mL) and (b) sprayed with an overnight culture of S. aureus (10 µL of OMW1 at 10 mg/l and 10 µL of GTOW at 50 mg/mL). OMW1: olive mill wastewater from a semi-modern process, GTOW: green table-olive wastewater phenolic extract.

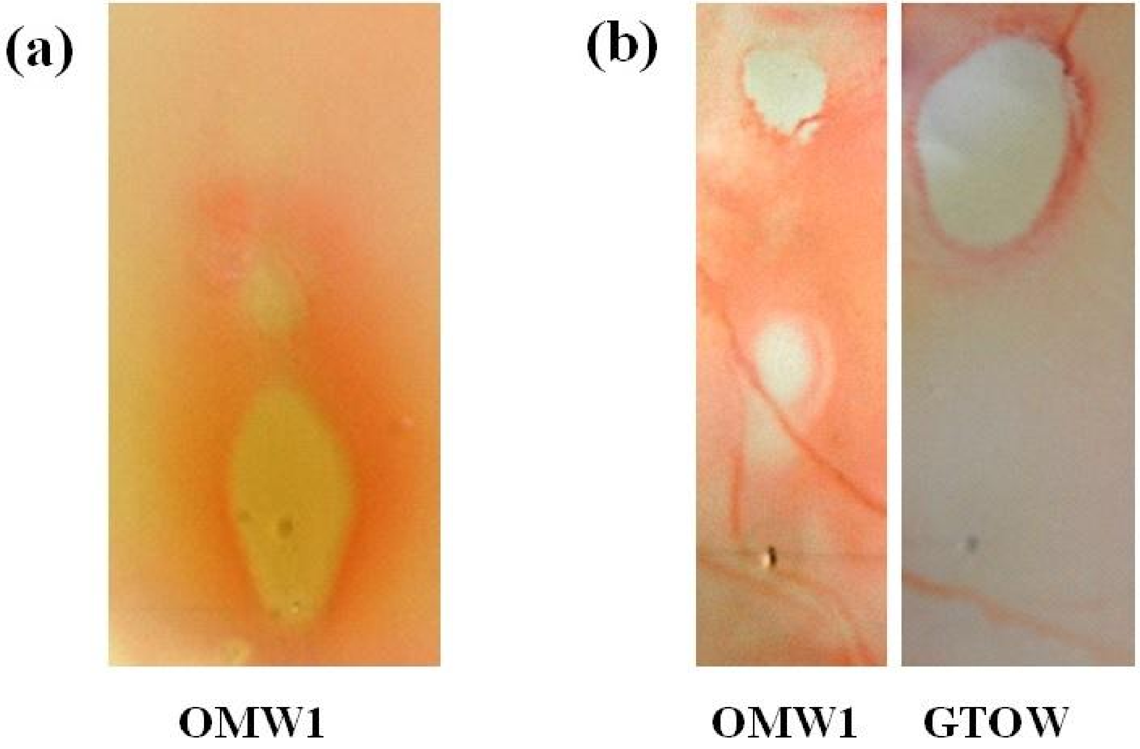

These results indicate clearly that phenolic compounds from OPW act synergistically and give higher antibacterial activity compared to individual component action.

It is also possible that synergism plays a major role in extracts that were active when the minimum inhibitory concentration of the mixture (or crude) was determined, but the separated compounds had less antibacterial activity based on bioautography.
