# Supplementary material for: Assessment of the antioxidant and antibacterial activities of different olive processing wastewaters
Source: PLoS One. 2017 Sep 5;12(9):e0182622. doi: 10.1371/journal.pone.0182622 (PMC5584791; doi:10.1371/journal.pone.0182622)
Supplement: S1 File — (PDF) [file pone.0182622.s002.pdf]

S1 File. HPLC conditions.

Solvent A = Water + 1% formic acid

Solvent B = MeOH:Acn:formic acid

Gradient run:

10 min = 90% A, 10% B ;20 min = 70% A, 30% B; 25 min = 70% A, 30% B; 35 min = 60% A, 40% B; 50 min = 50% A, 50% B; 60 min = 0% A, 100% B; 65 min = 90% A, 10% B; 75 min = 90% A, 10% B

Total run = 75 min, 2 min post-run, flow rate = 0.2 mL/min, injection volume = 10 µL.

Detection wavelength = 240, 280, 365 nm.
