# Supplementary material for: Assessment of the antioxidant and antibacterial activities of different olive processing wastewaters
Source: PLoS One. 2017 Sep 5;12(9):e0182622. doi: 10.1371/journal.pone.0182622 (PMC5584791; doi:10.1371/journal.pone.0182622)

S2 File. LC-MS conditions.

LC conditions same as HPLC condition.

MS conditions:

1. Hydroxytyrosol = negative mode, scan between m/z 15-500, target ion at m/z 153. MS/MS 153. Other peaks available = 122.8

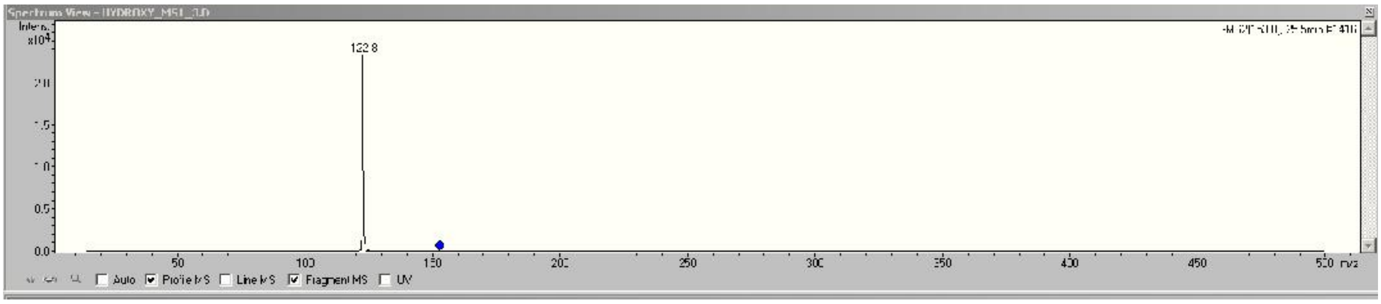

2. Tyrosol = negative mode, scan between m/z 15-500, target ion at m/z 137. MS/MS 137. Other peaks available = 118.8, 105.9, 92.3, 81

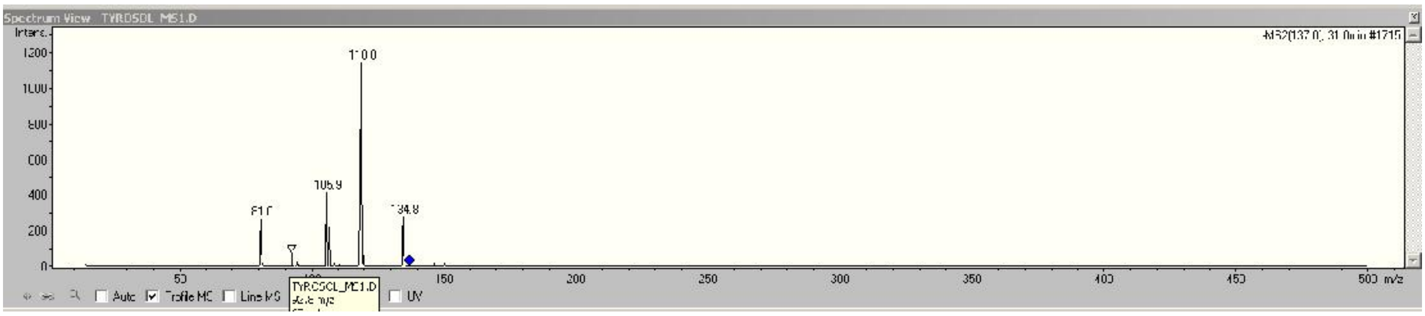

3. Oleuropein = negative mode, scan between m/z 15-800, target ion at m/z 540. MS/MS 540. Other peaks available = 377 (main peaks from journal article), 344.9, 327, 307, 275.

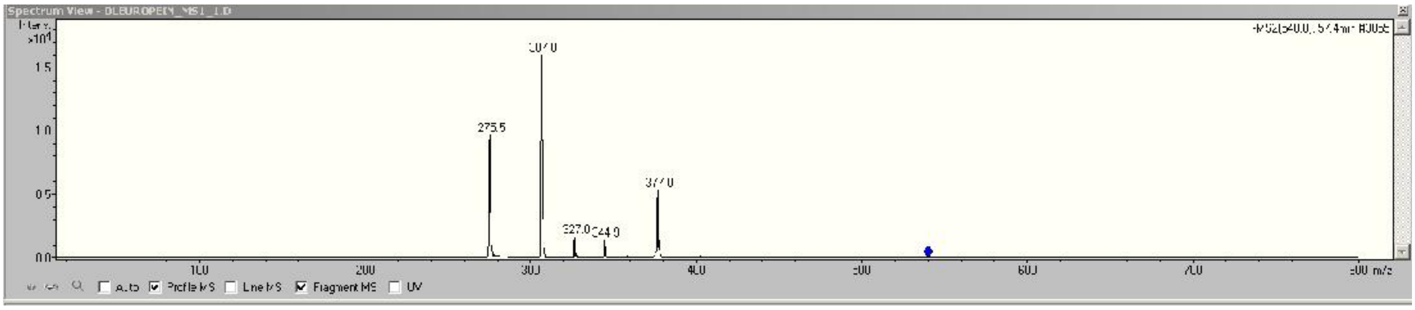

4. P-coumaric acid = negative mode, scan between m/z 15-500, target ion at m/z 164. MS/MS 164. Other peaks available = 118.7.

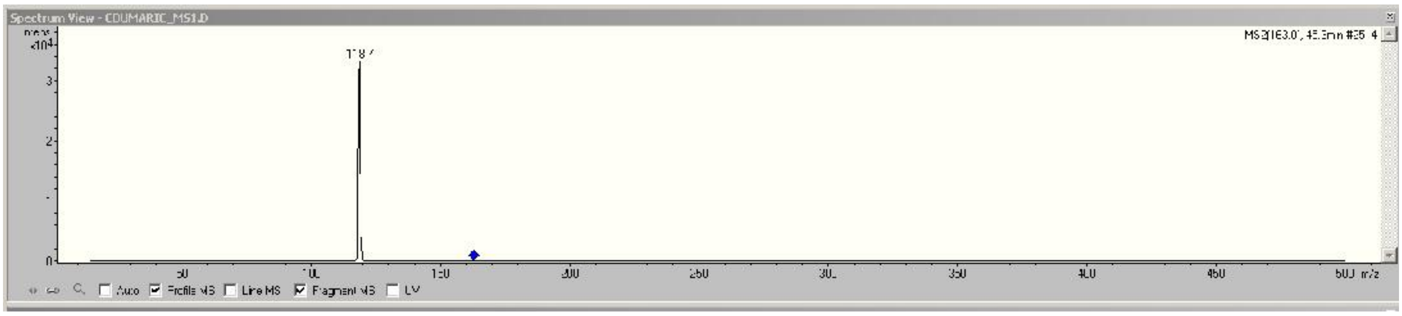

5. Ferrulic acid = negative mode, scan between m/z 15-500, target ion at m/z 193. MS/MS 193. Other peaks available = 177.7, 148.9 and 134.2.

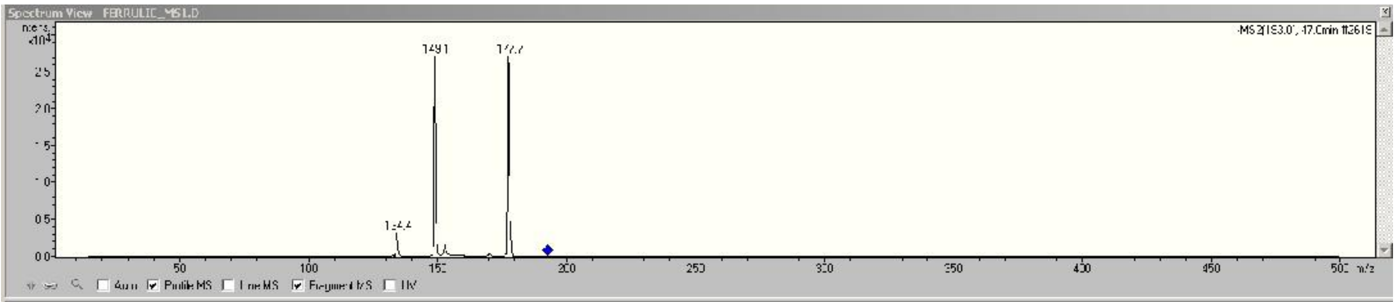

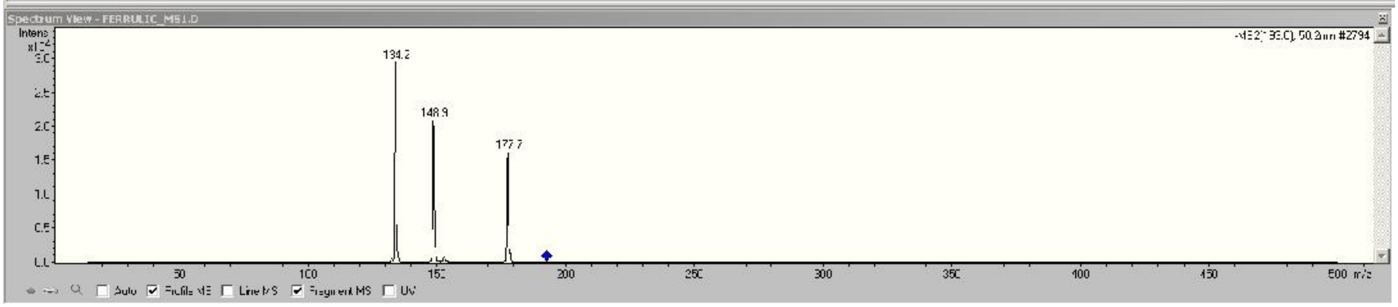

Supplement: S2 File — (PDF) [file pone.0182622.s003.pdf]
