# Supplementary material for: Assessment of the antioxidant and antibacterial activities of different olive processing wastewaters
Source: PLoS One. 2017 Sep 5;12(9):e0182622. doi: 10.1371/journal.pone.0182622 (PMC5584791; doi:10.1371/journal.pone.0182622)

S3 File. HPLC and MS chromatogram of OMW samples.

SI_hydroxytyrosol (area=23514.1)

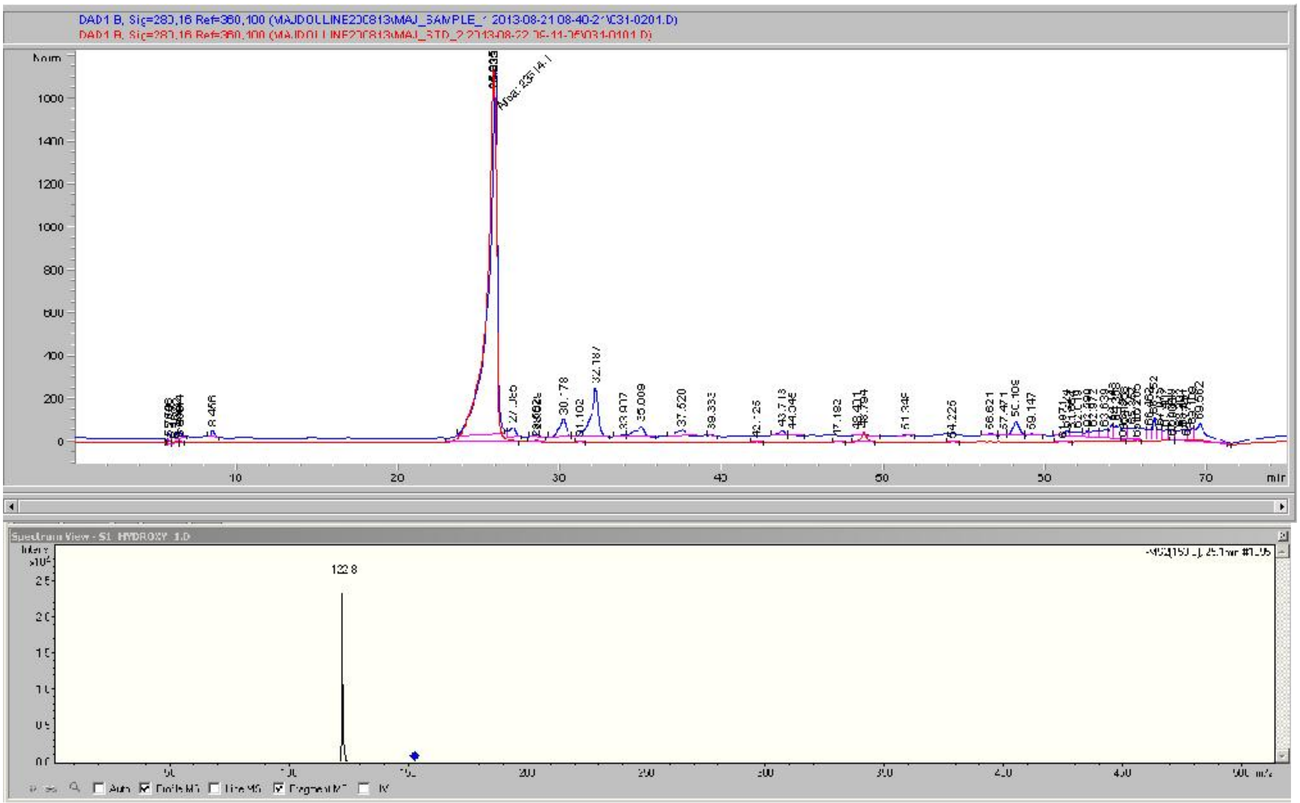

S1_Tyrosol (area=2351.81)

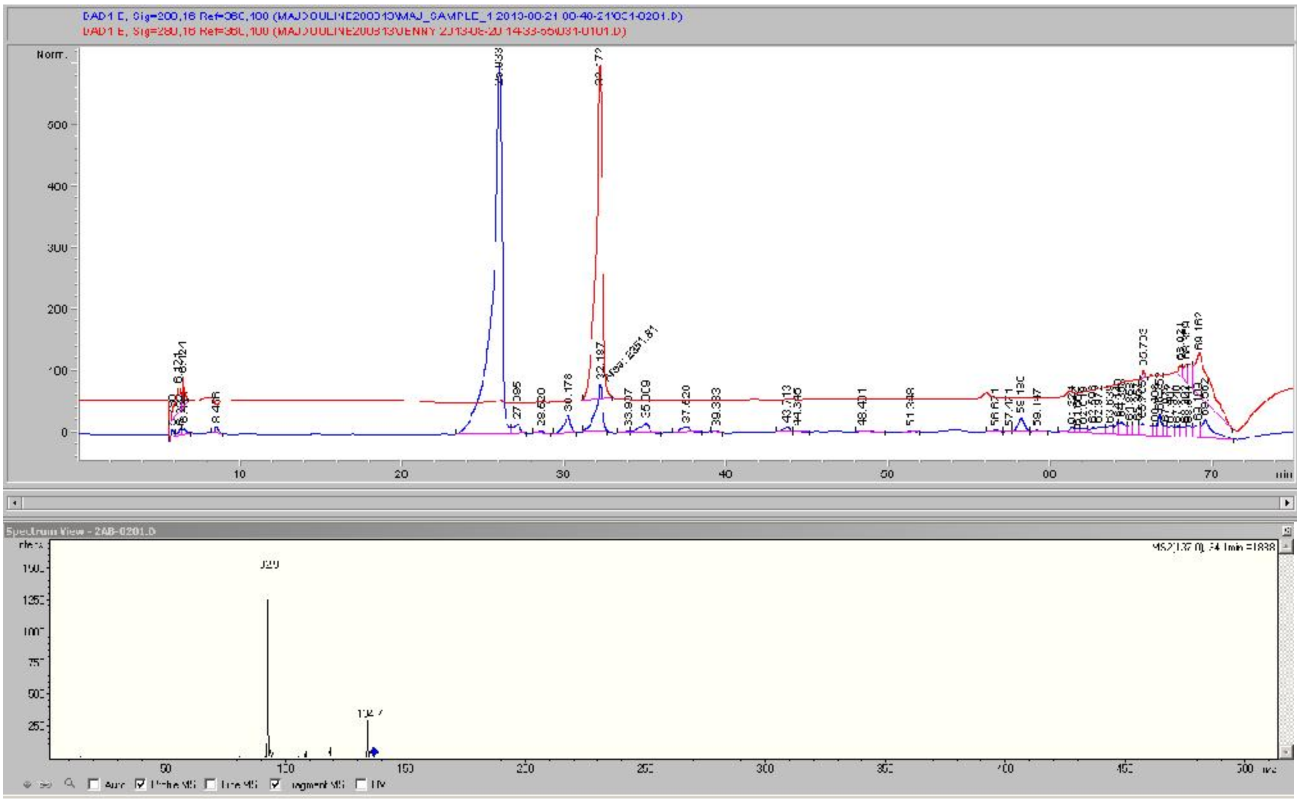

S1_Oleuropein (area=562.312)

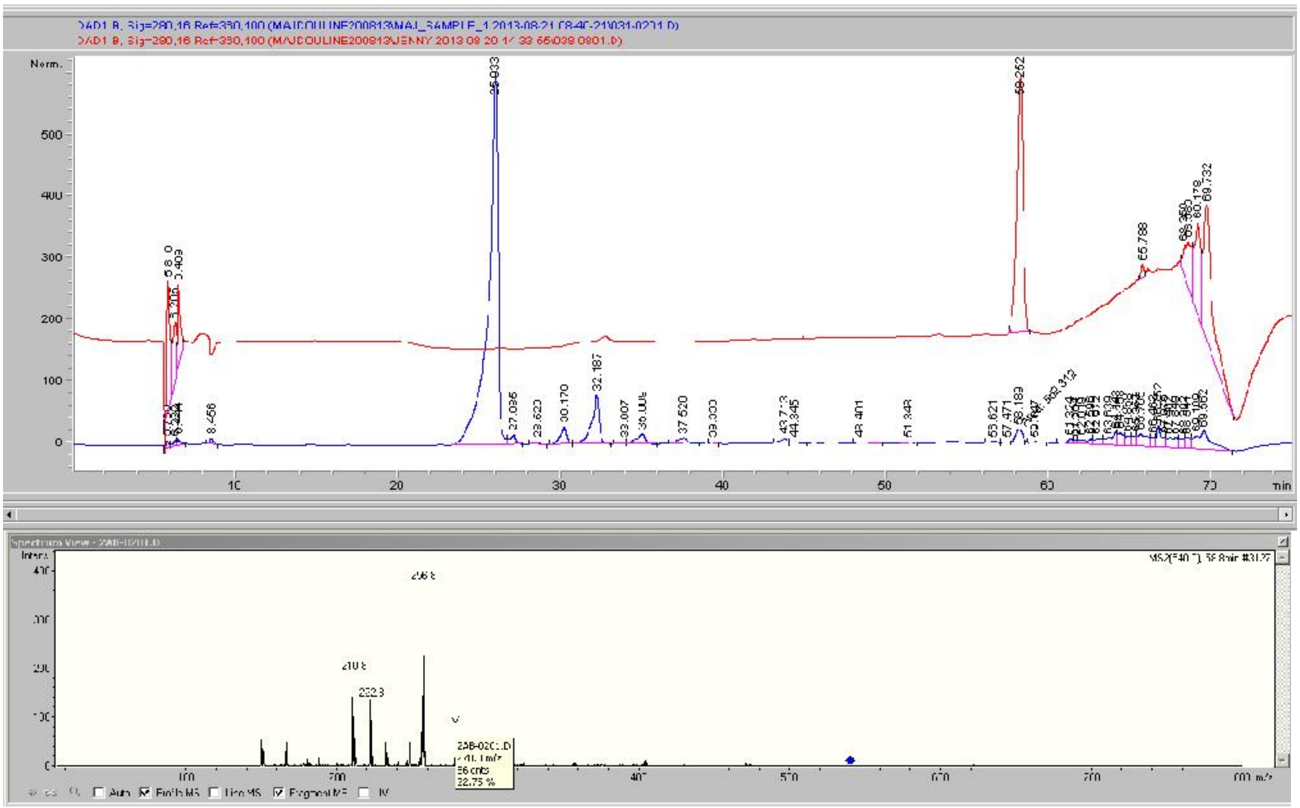

S1_P-coumaric (area=72.3991)

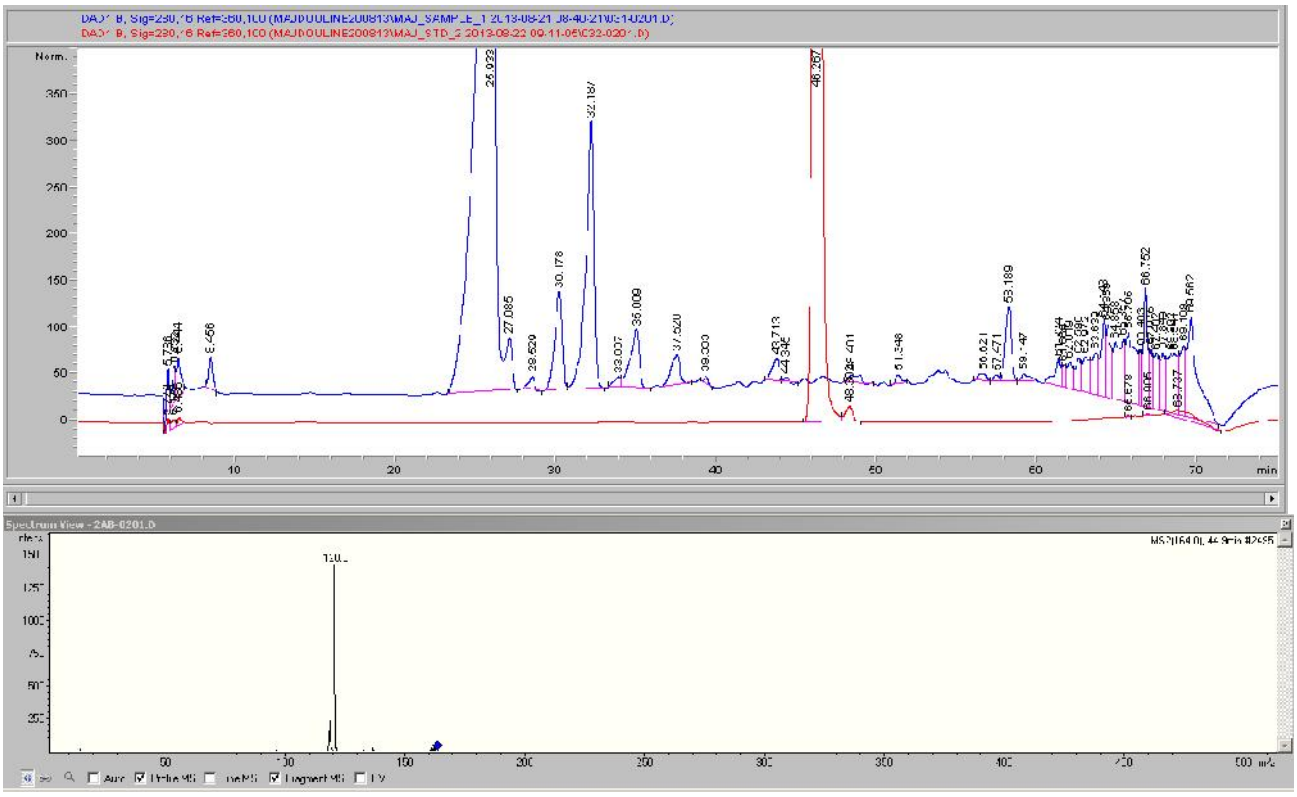

S2_Hydroxytyrosol (area=4024.34)

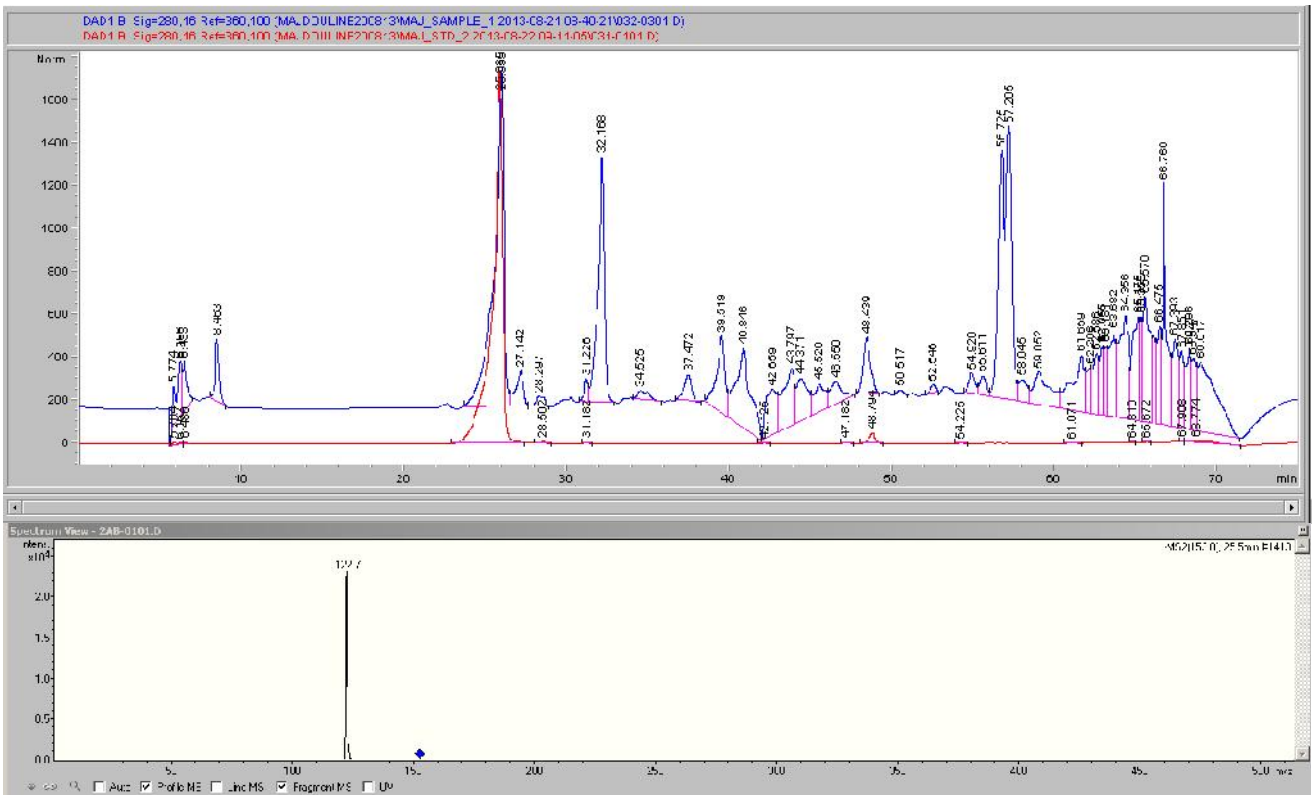

S2_Tyrosol (area=1999.67)

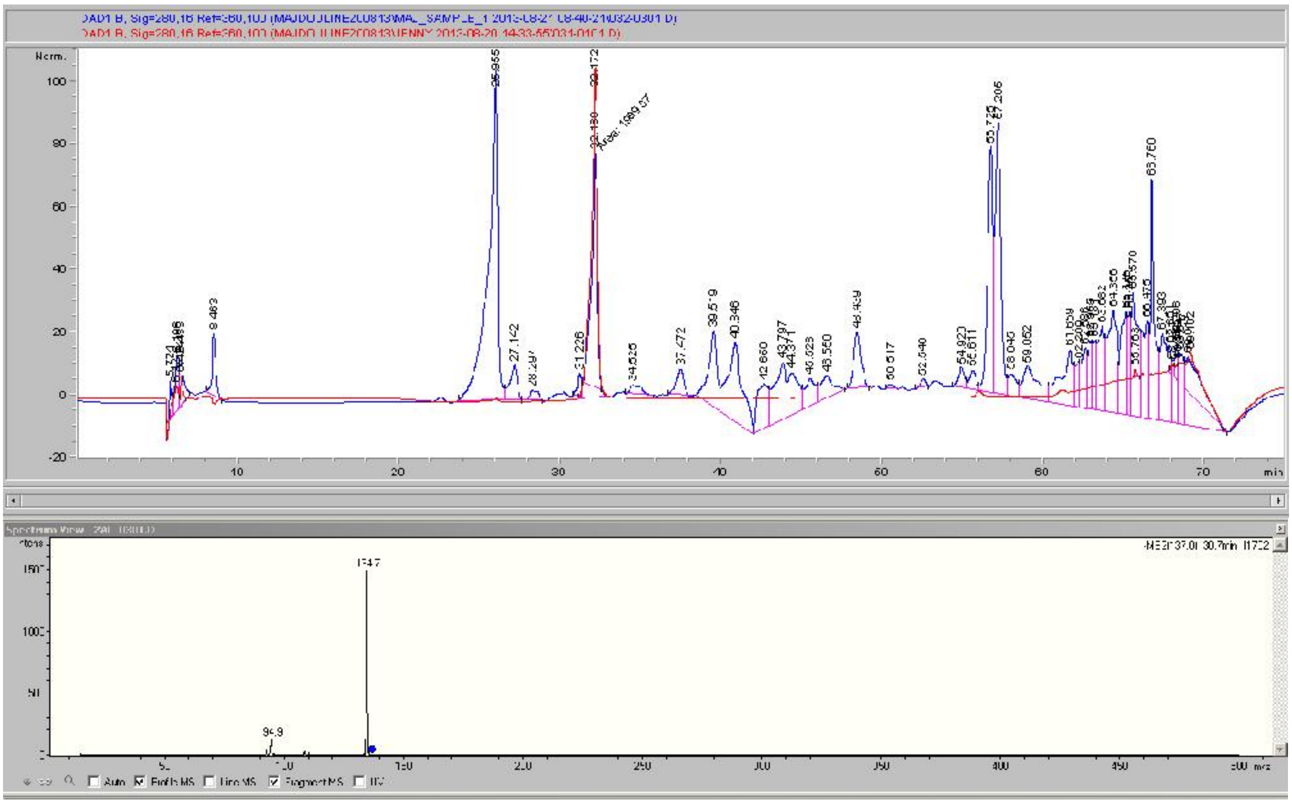

S2_Oleuropein (area=81.8678)

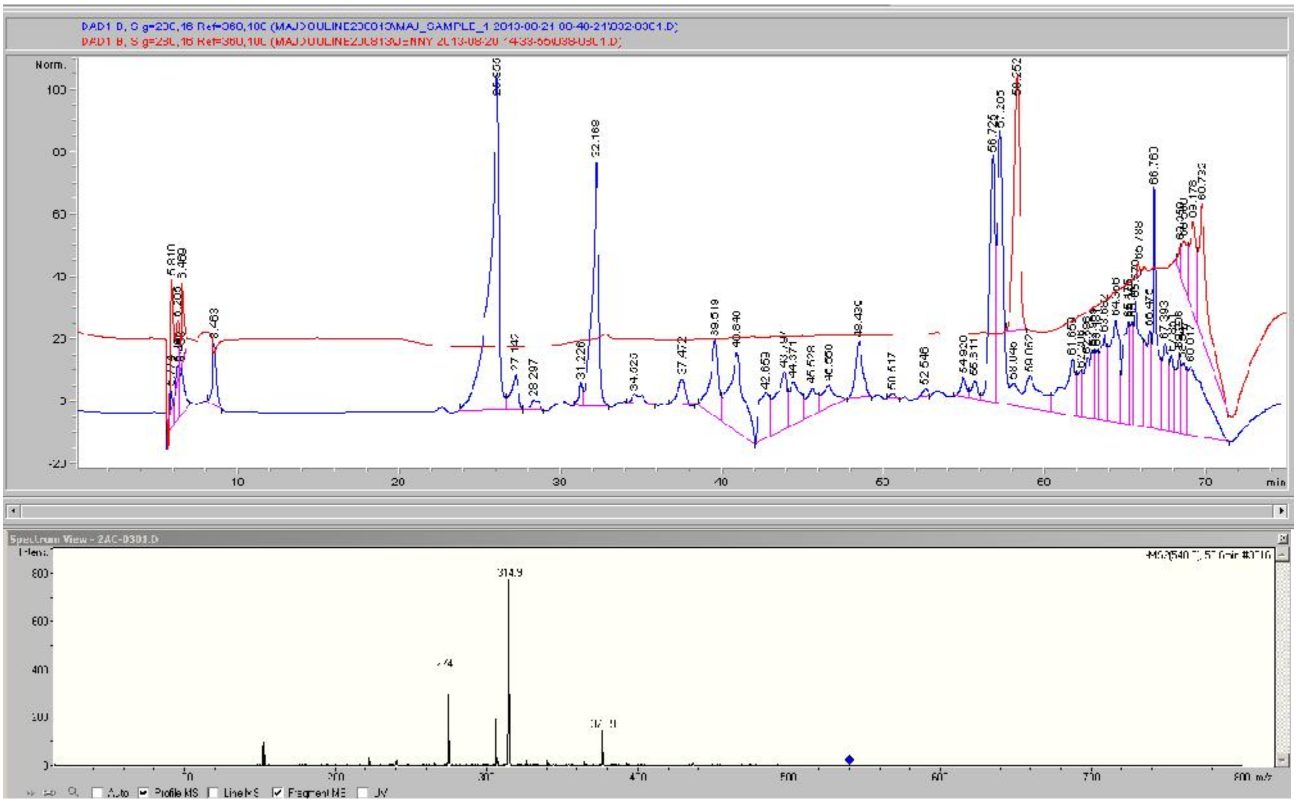

S2_P-coumaric (area= 500.266)

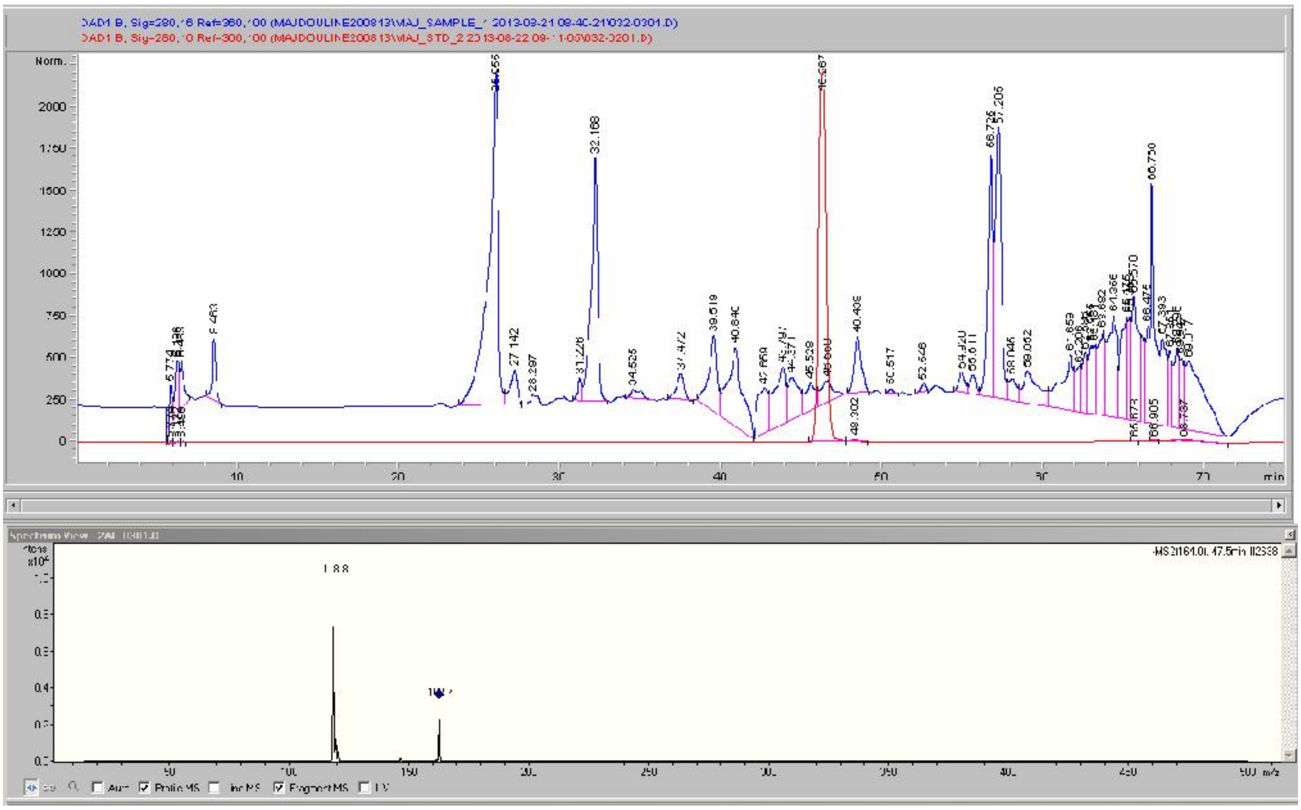

S3_hydroxytyrosol (area=15222.8)

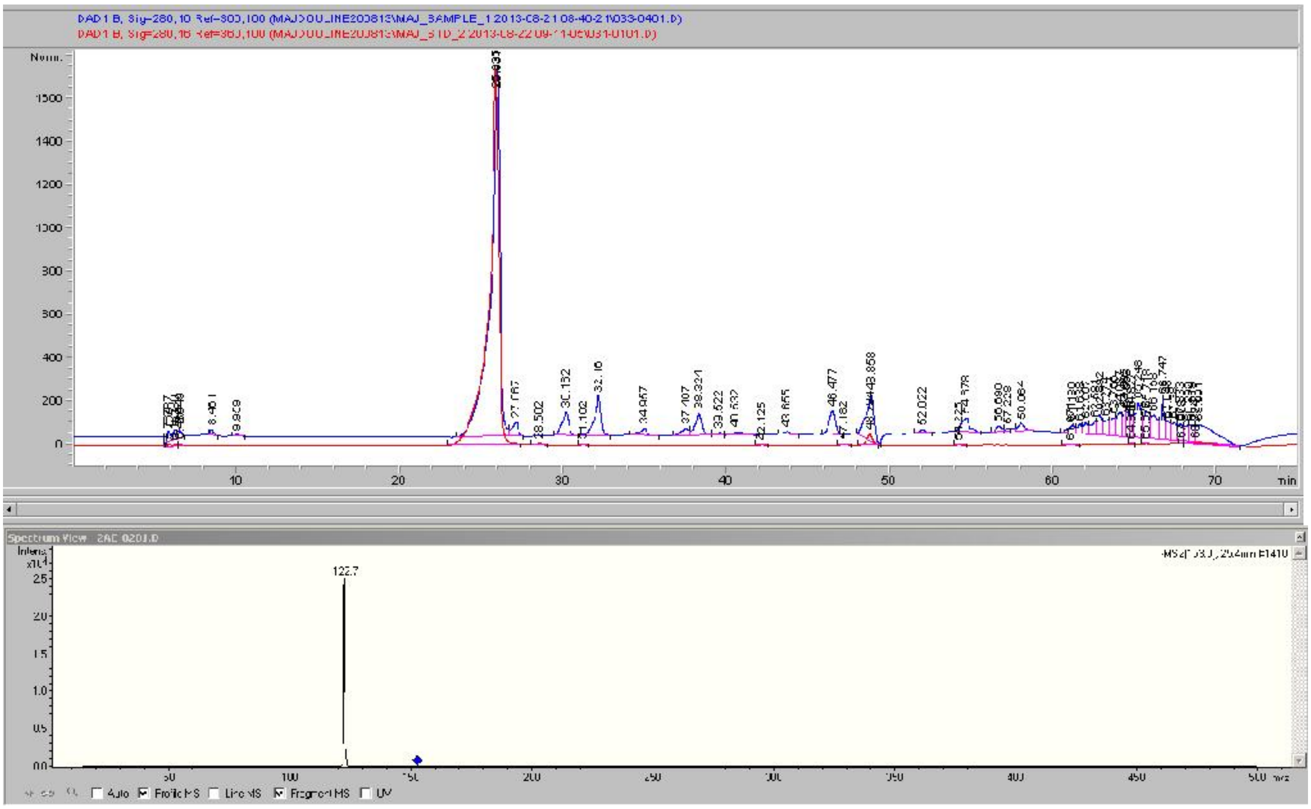

S3_Tyrosol (area=1194.17)

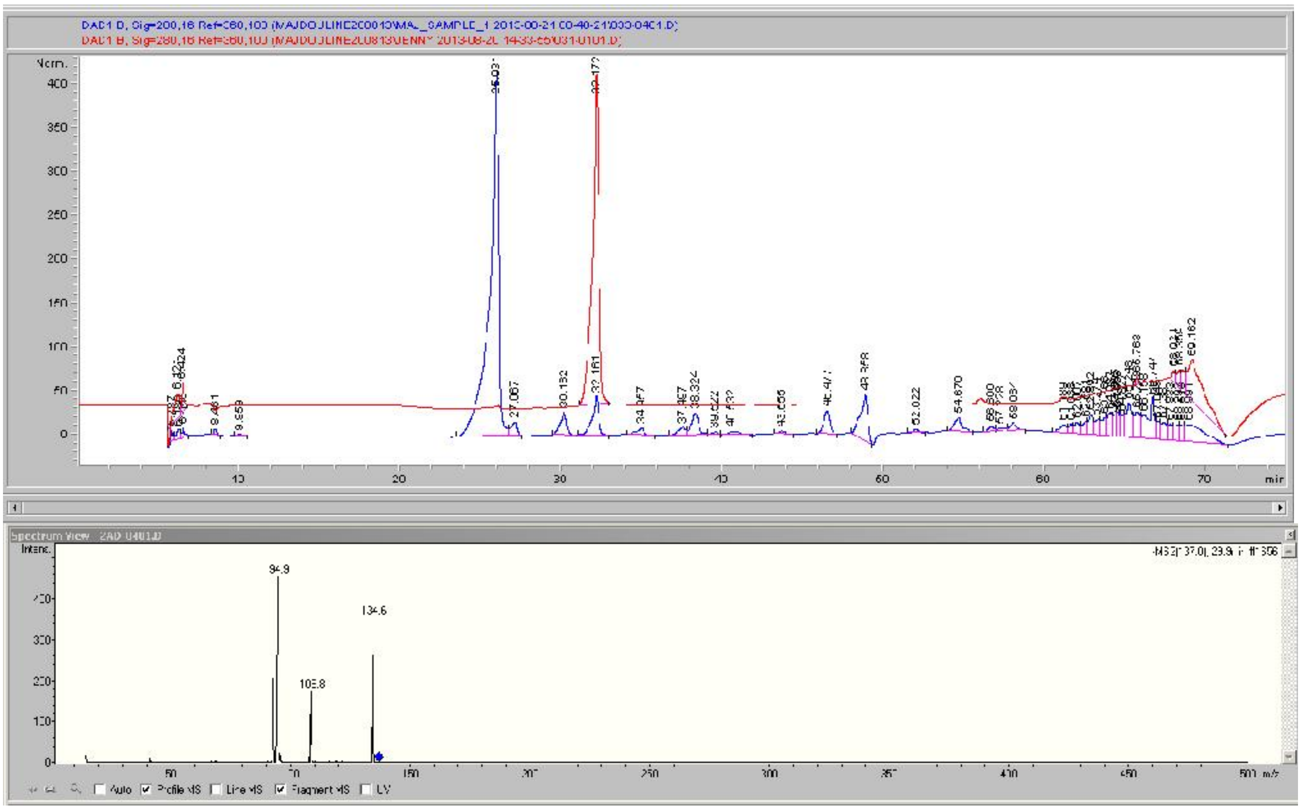

S3_Ferrulic acid (area=1855.89, 131.663)

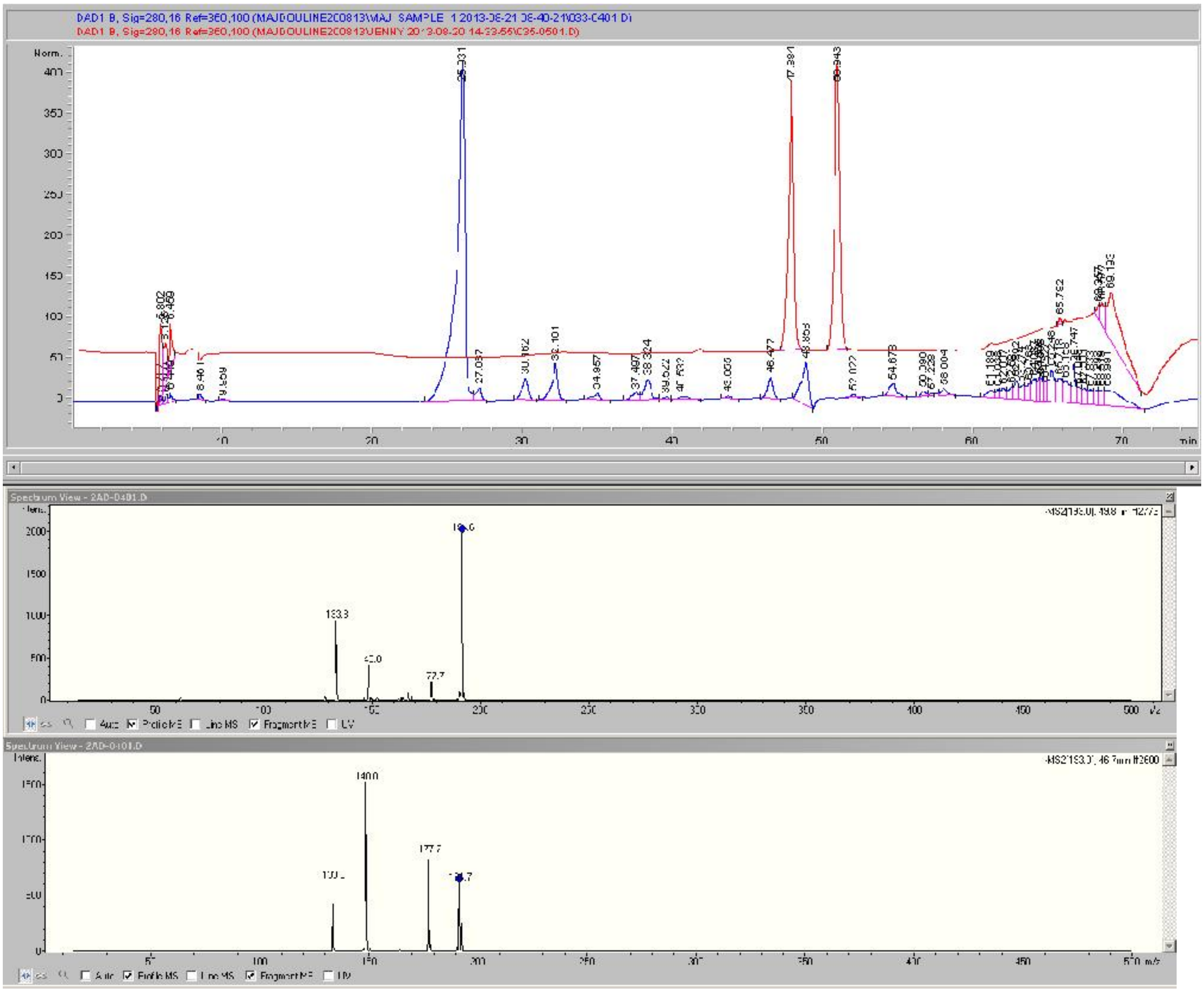

S3_Oleuropein (area=132.767)

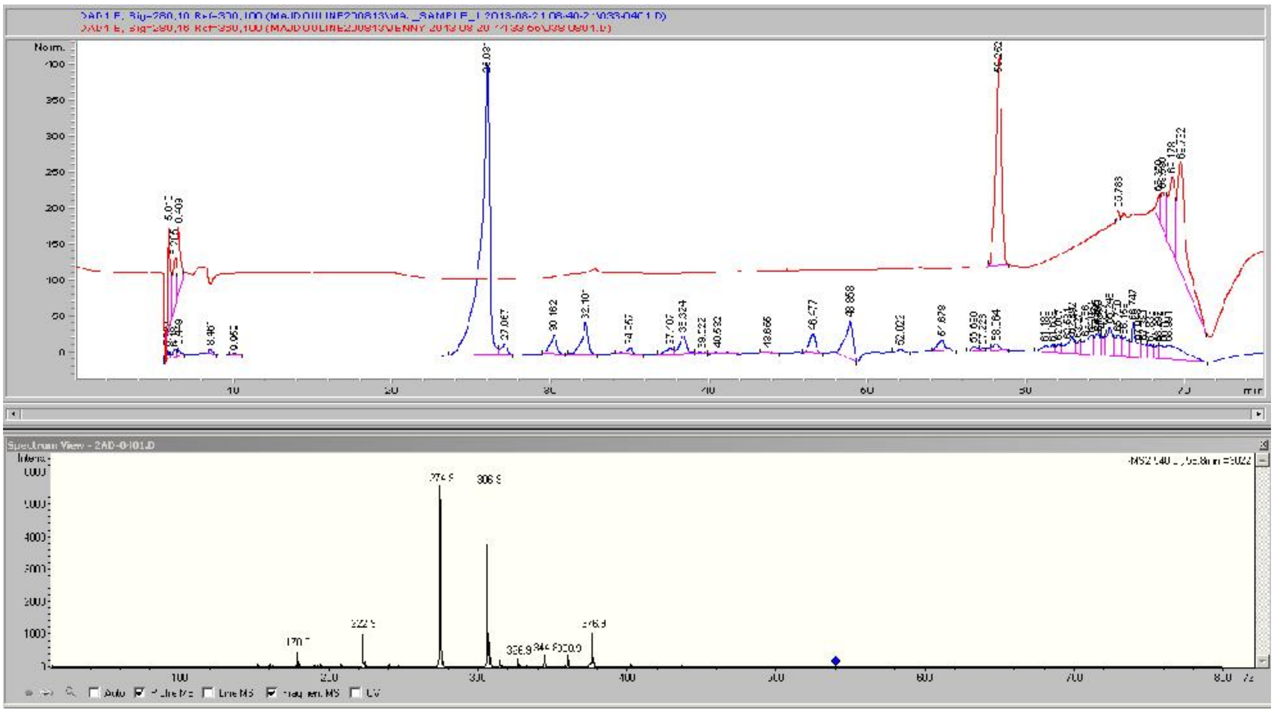

S3_P-coumaric acid (area =613.024)

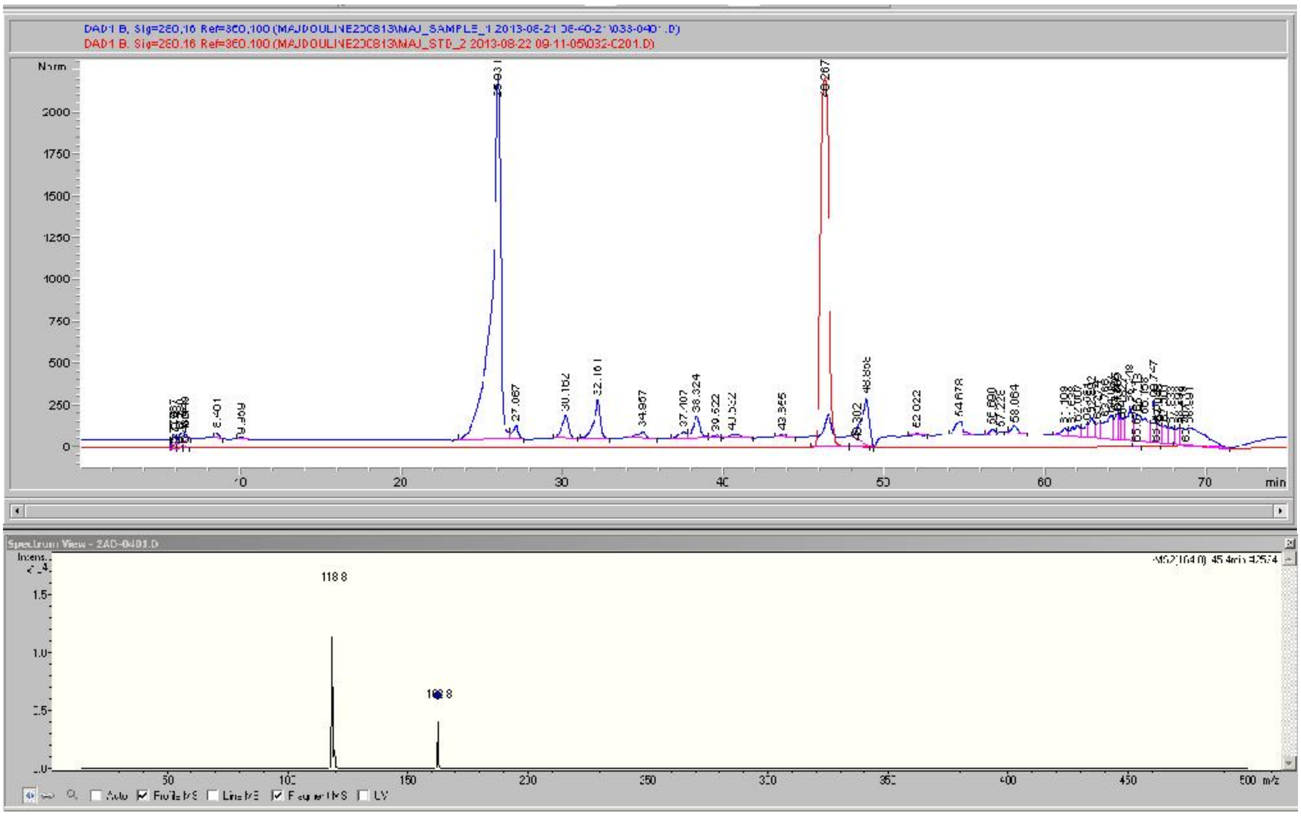

S4_Hydroxytyrosol (area=4471.02)

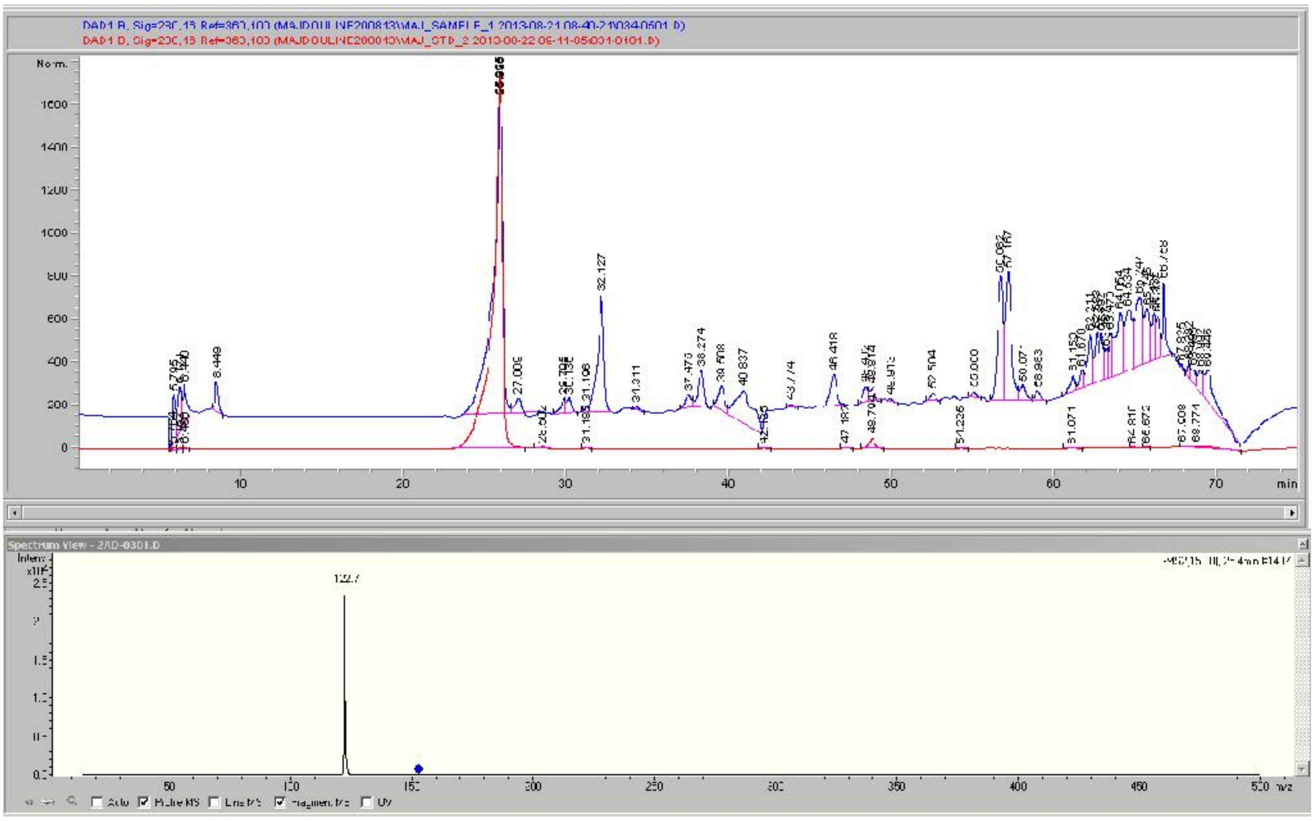

S4_Tyrosol (area=1069.74)

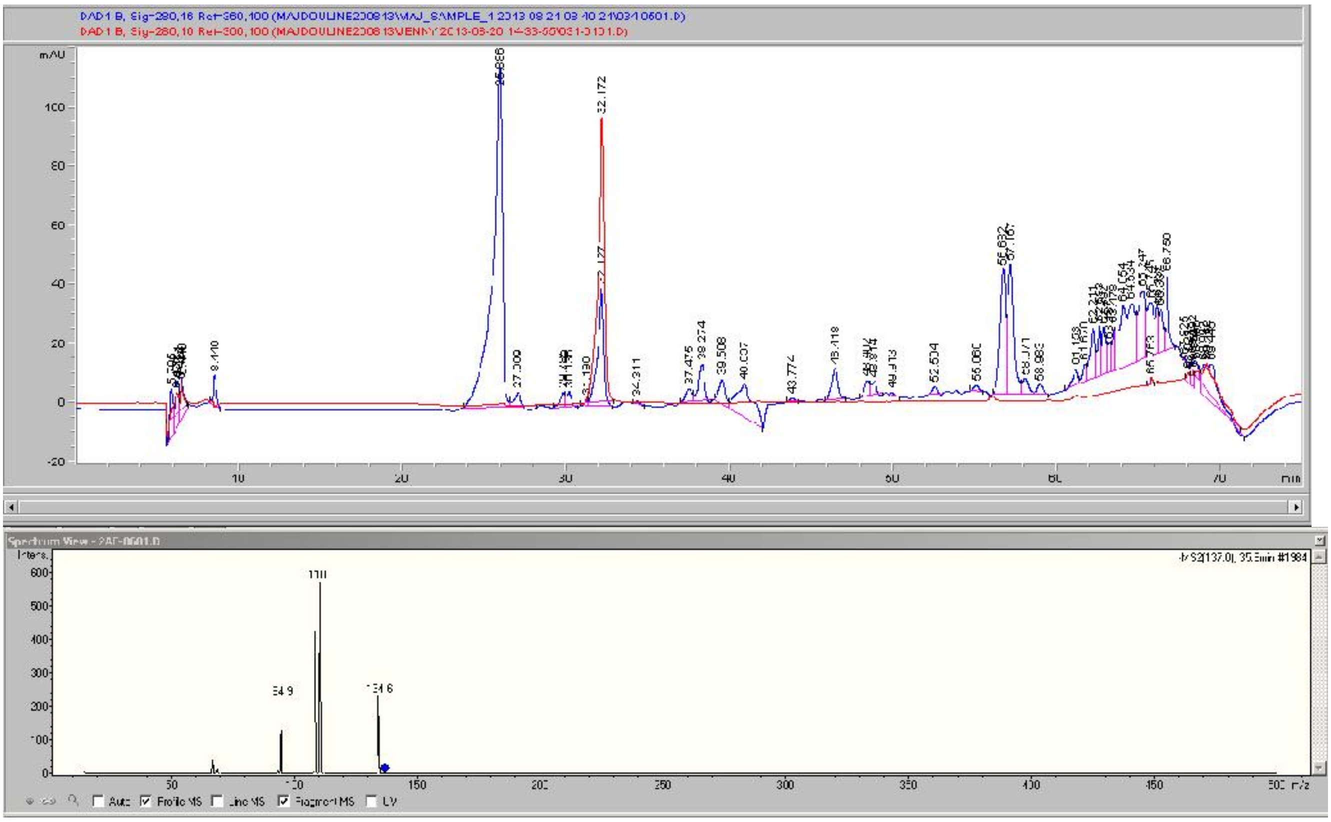

S4_P-coumaric acid (area=251.832)

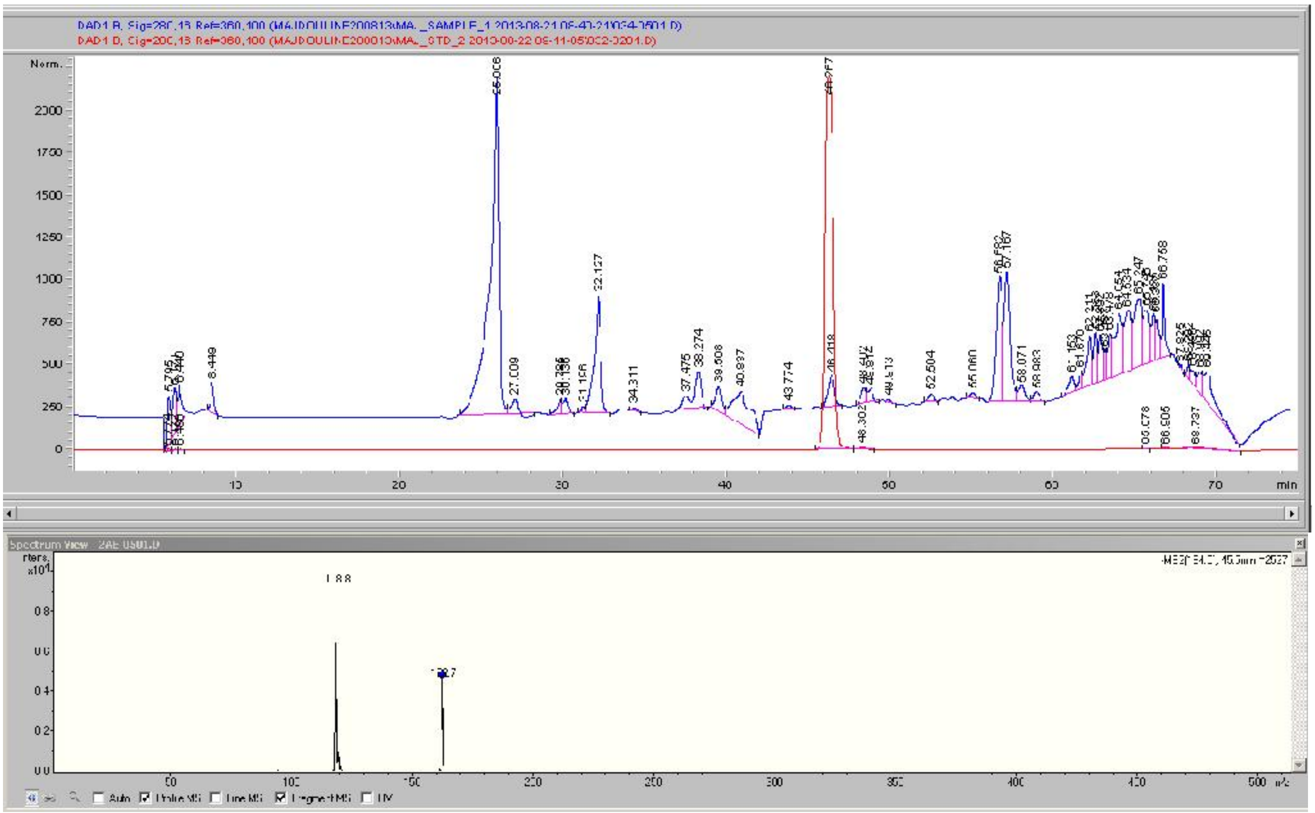

S4_Ferrulic acid (area=257.220, 36.74)

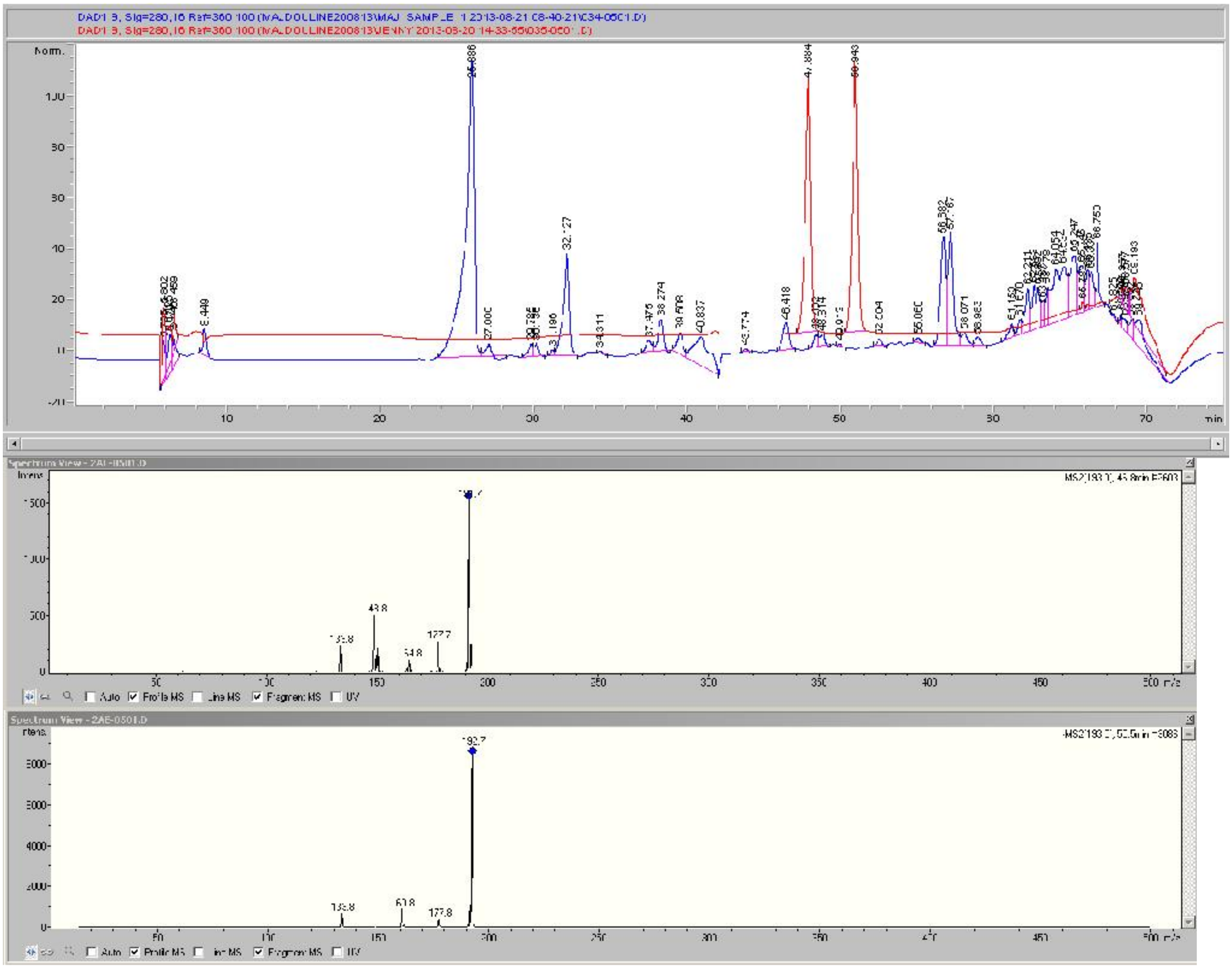

S4_Oleuropein (area=51.1121)

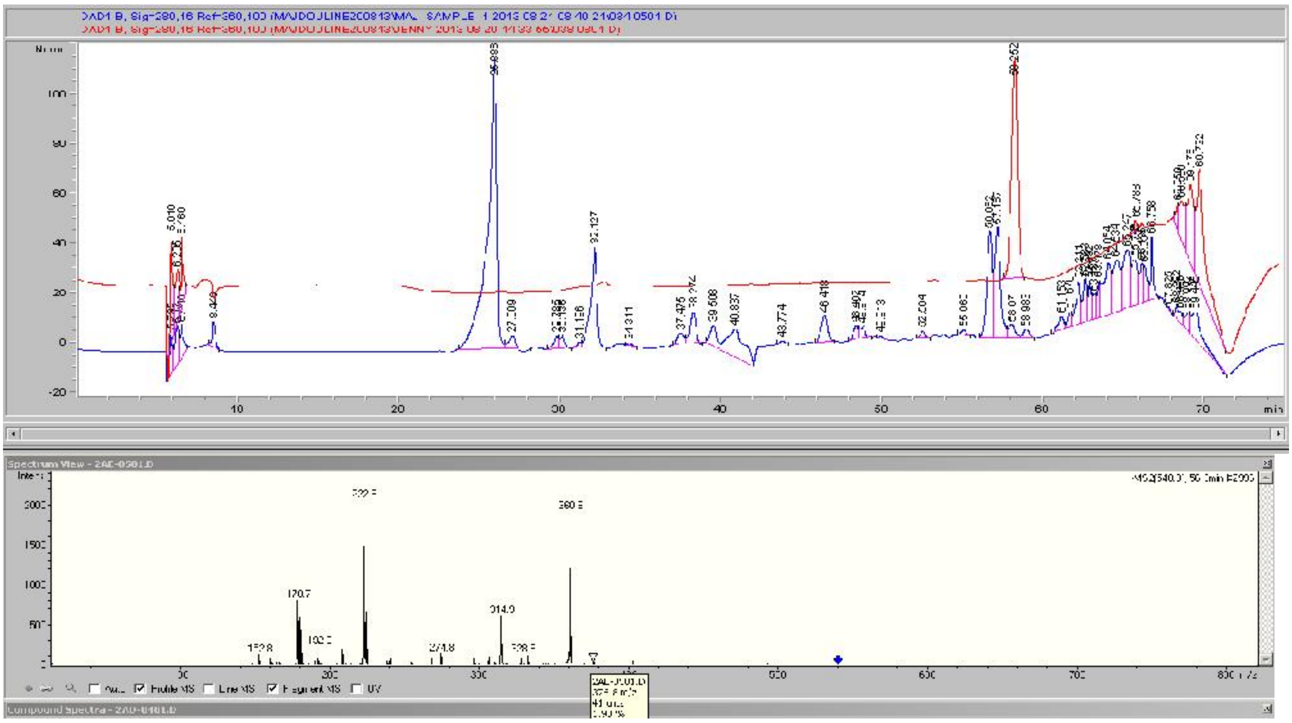

V_hydroxytyrosol (area=74211.3)

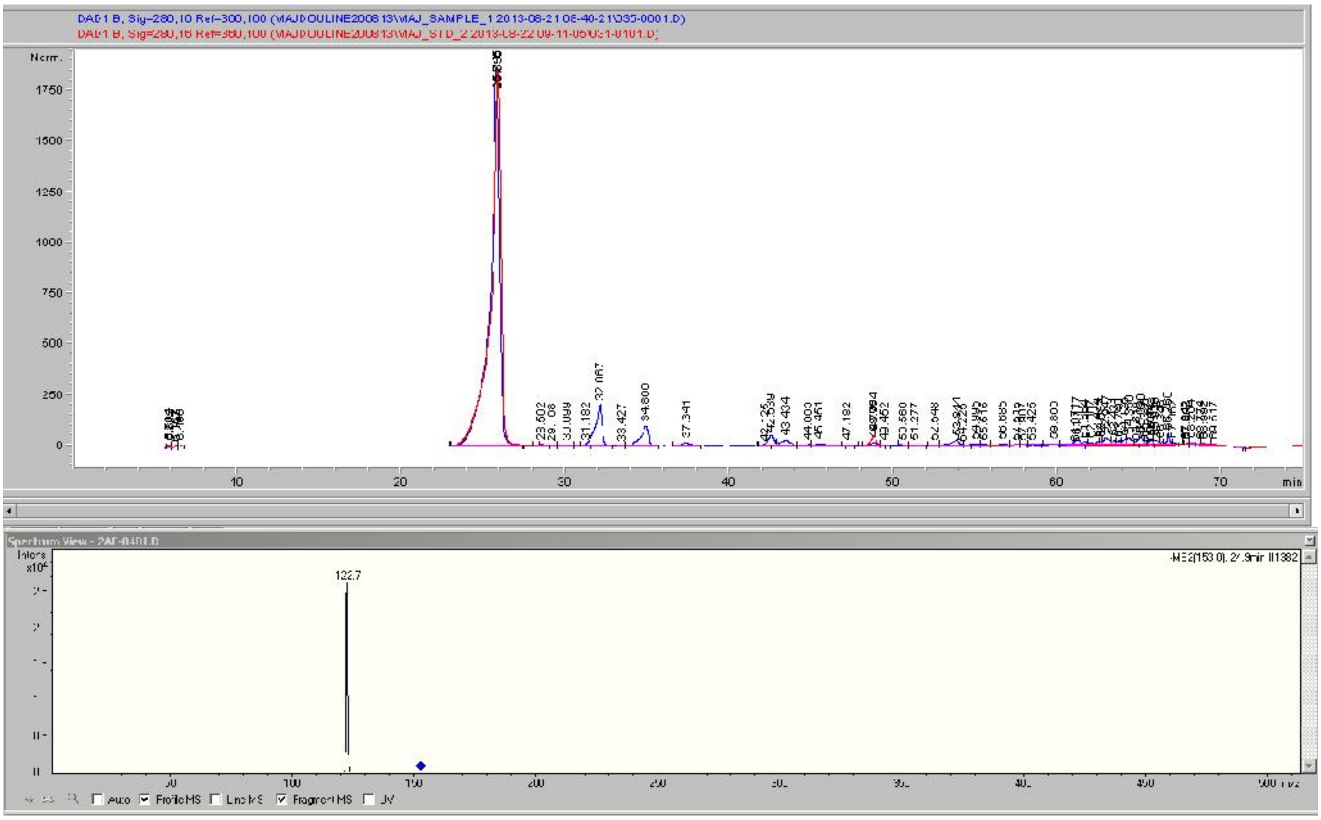

V_Tyrosol (area=5939.05)

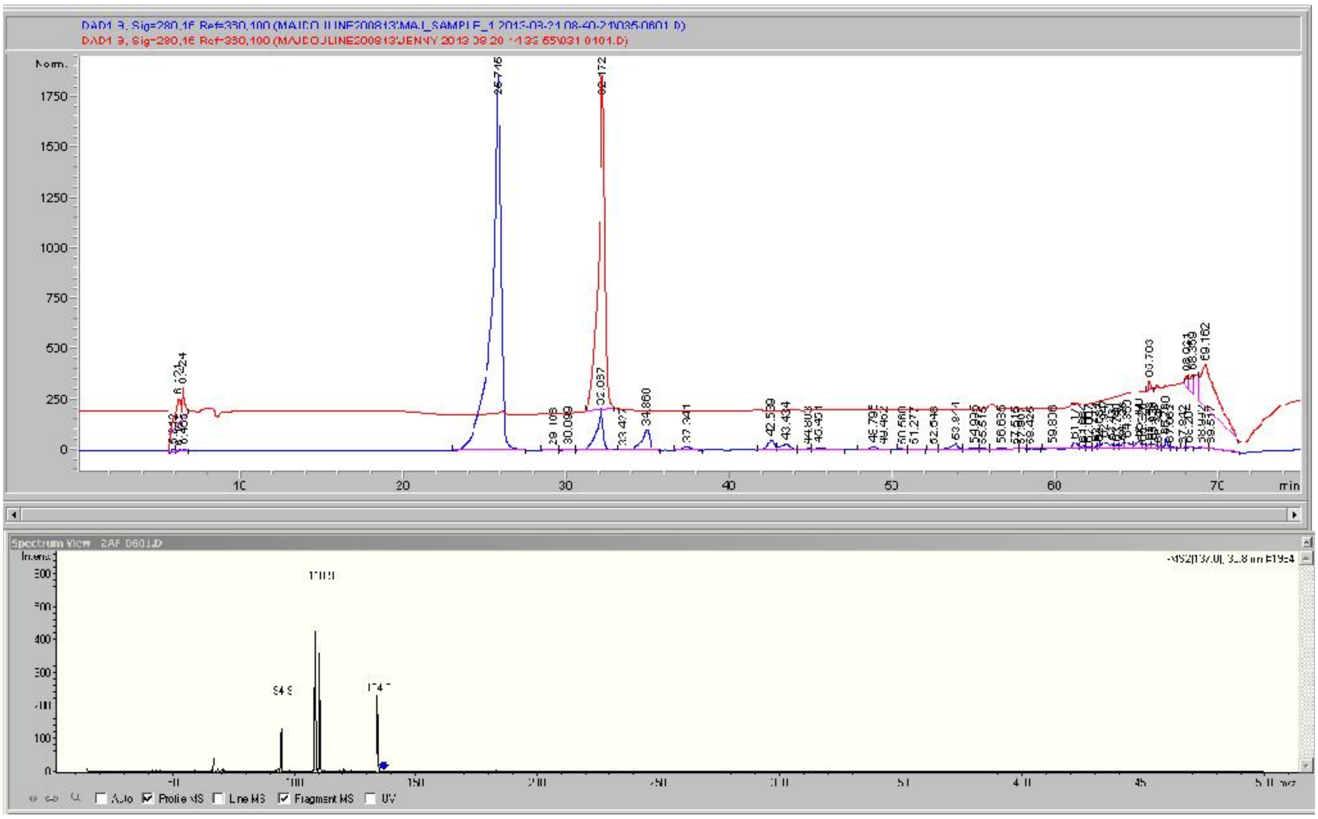

V_oleuropein (area=23.75)

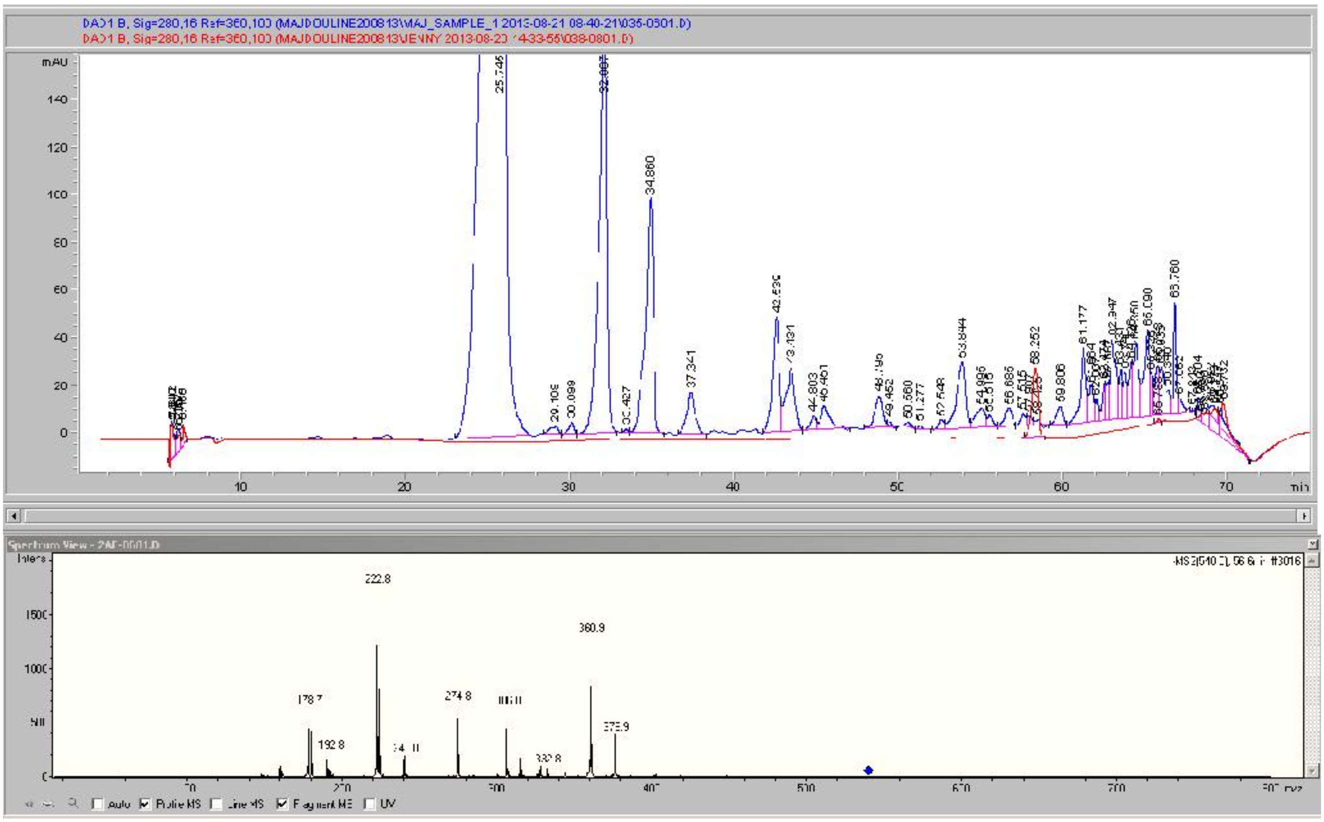

T_Hydroxytyrosol (area=43125.4)

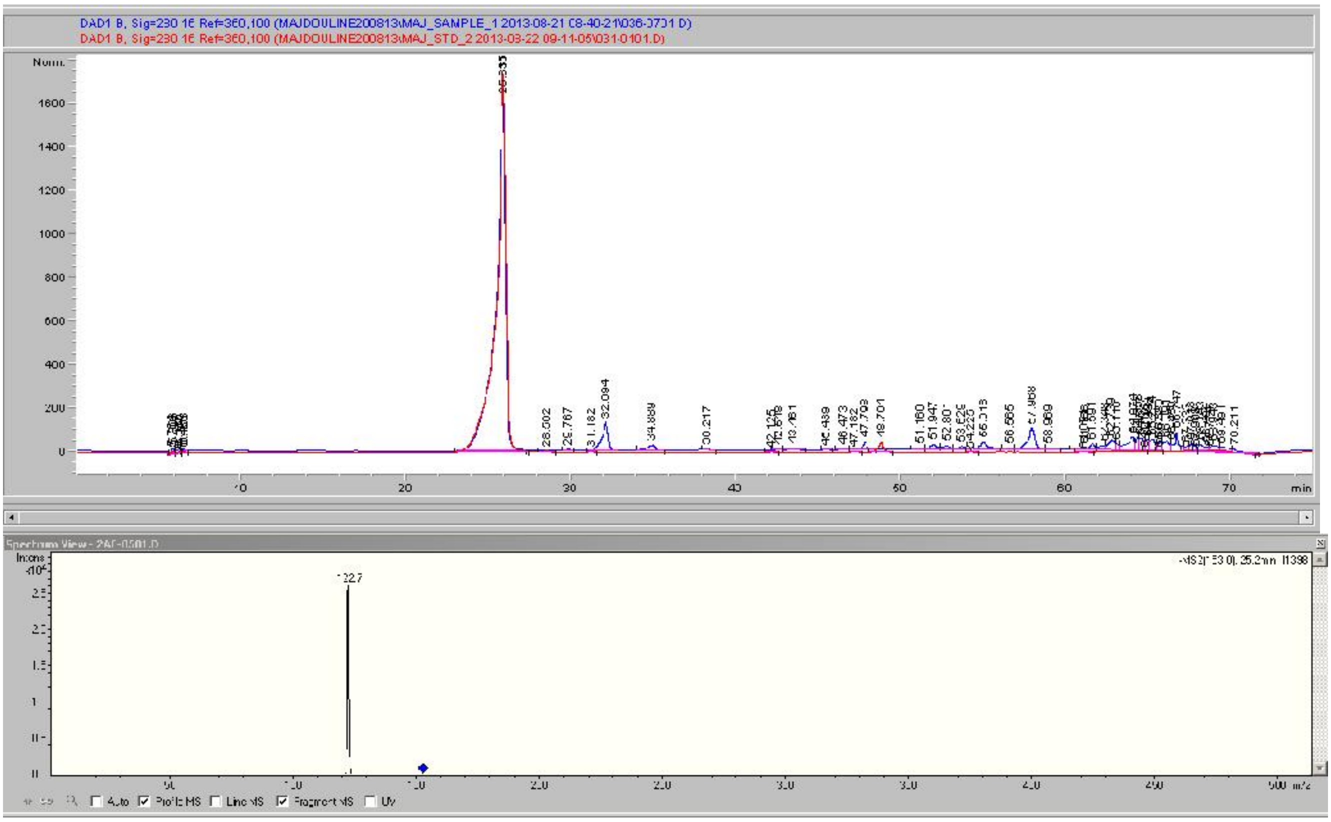

T_Tyrosol (area=2208.98)

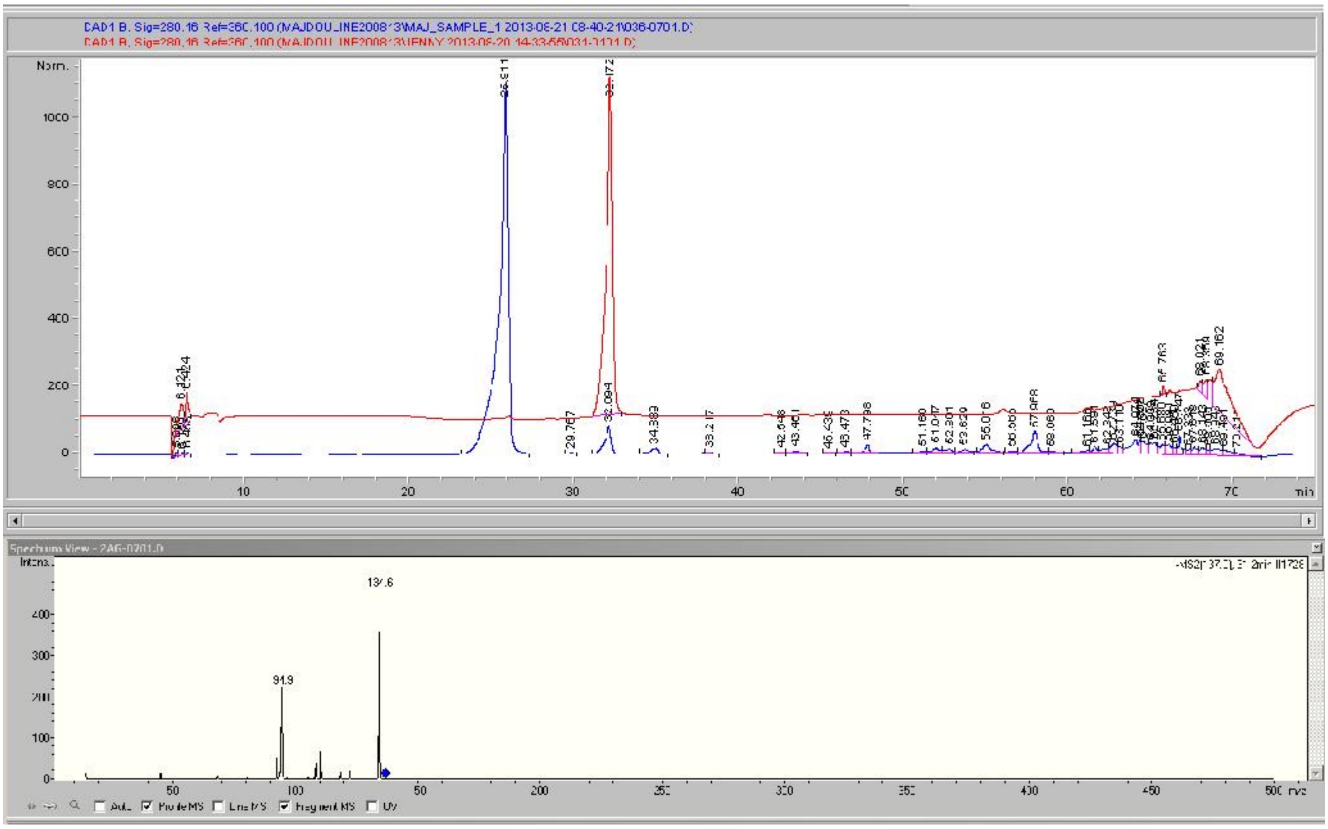

T_Oleuropein (area=1750.8)

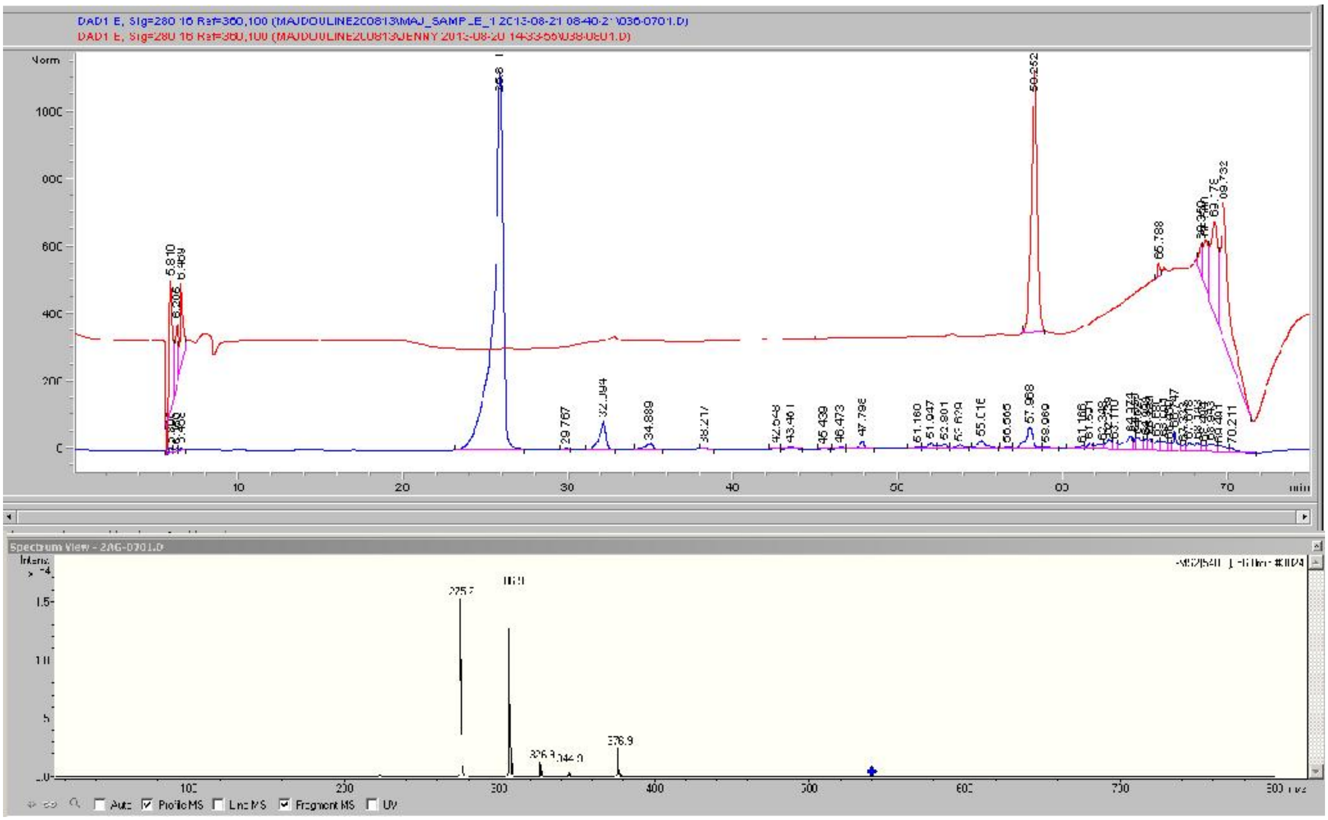

T_P-coumaric acid (area=282.313)

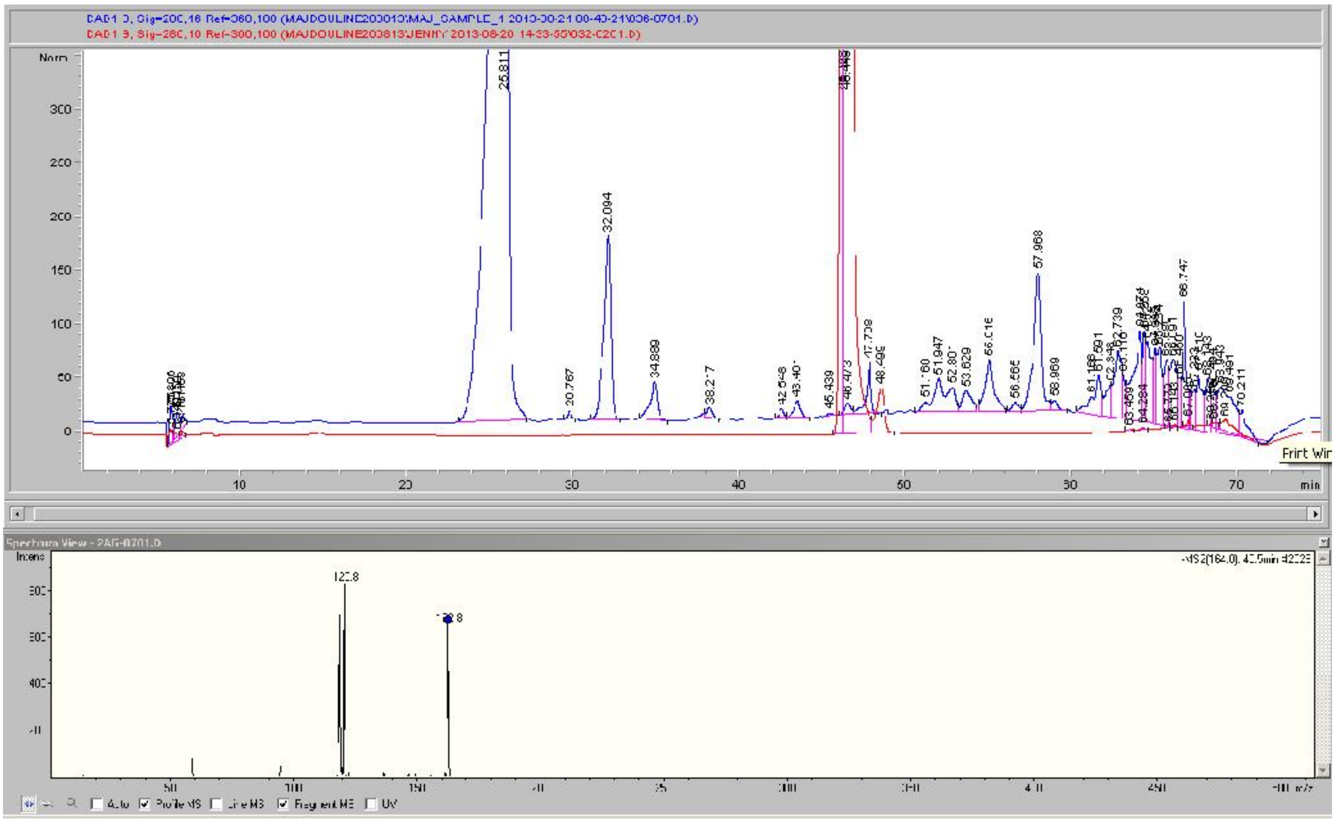

N_Hydroxytyrosol (area=9663.47)

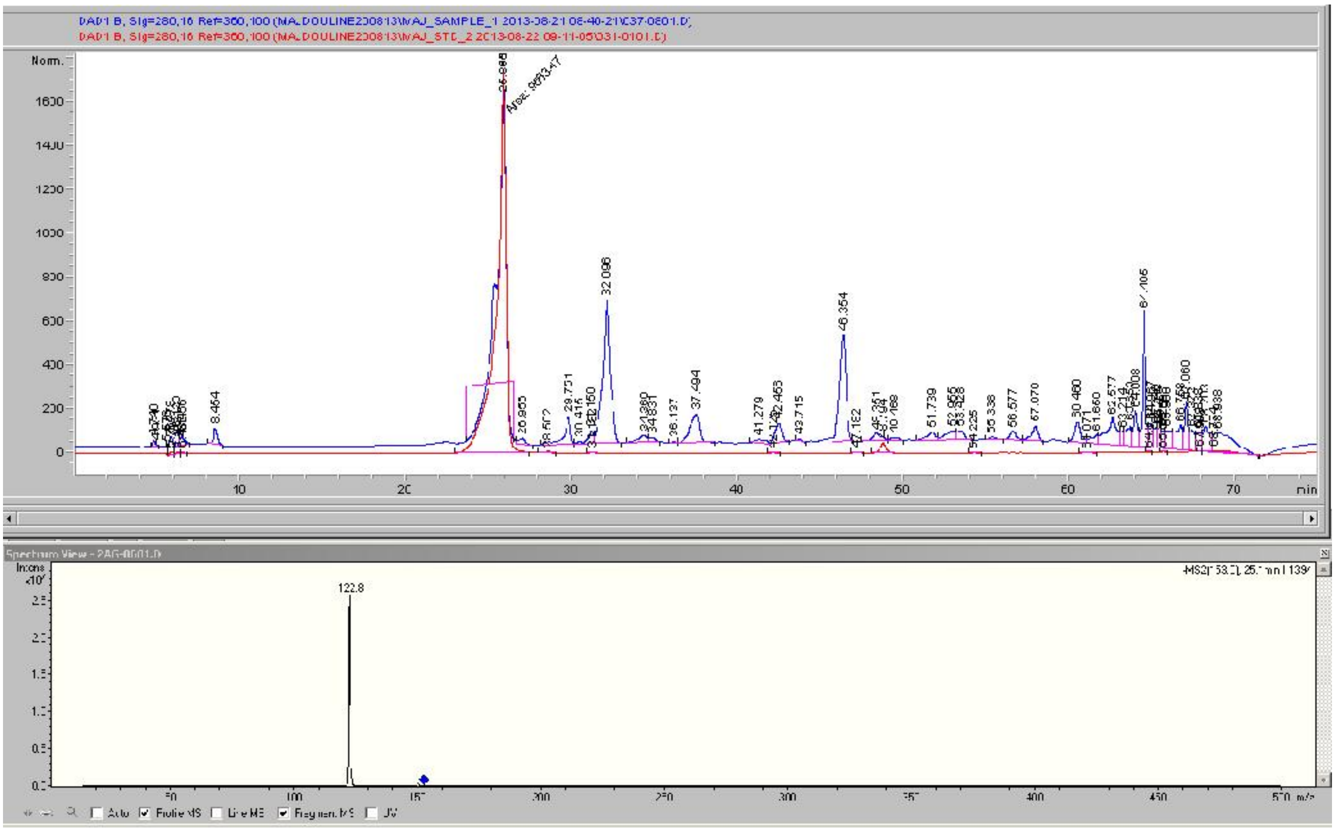

N_Tyrosol (area=4410.57)

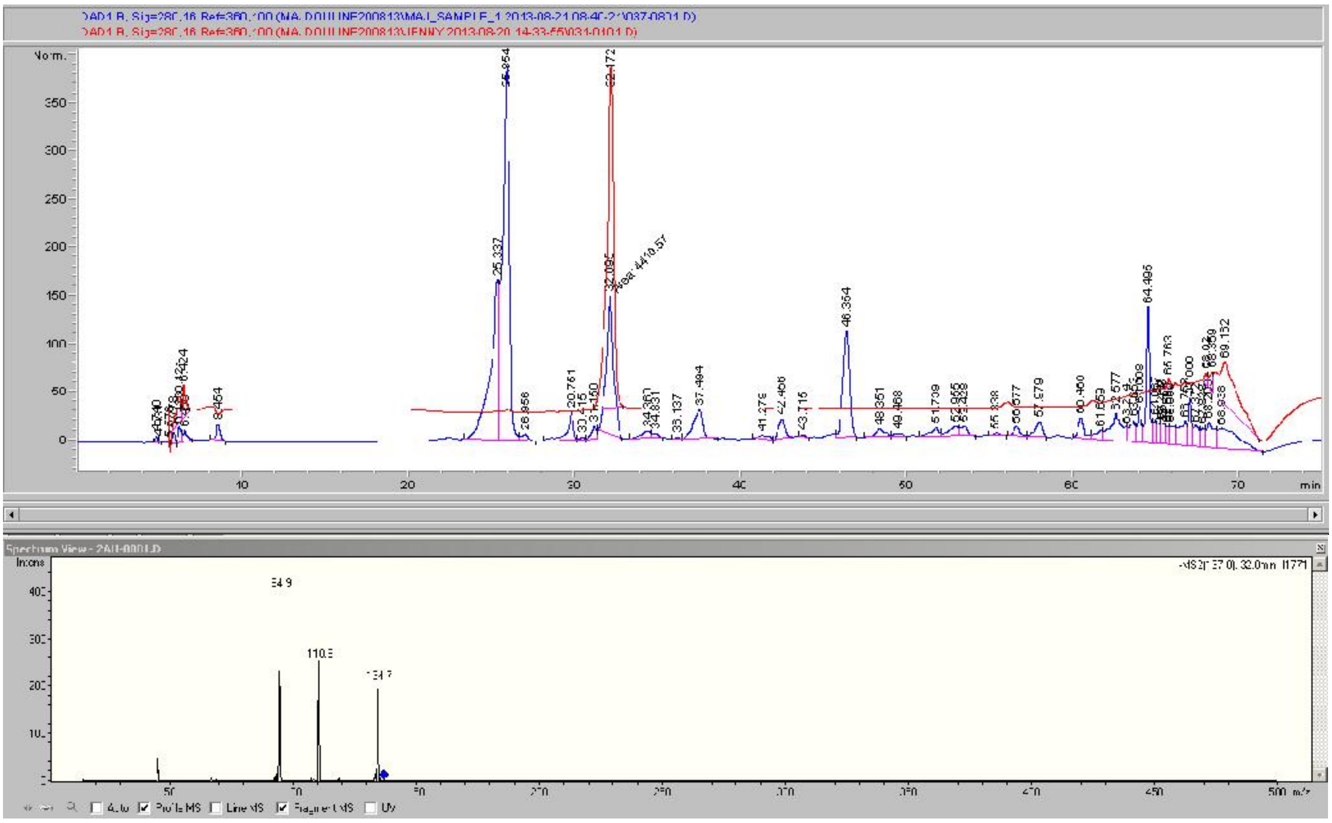

N_Oleuropein (area=388.036)

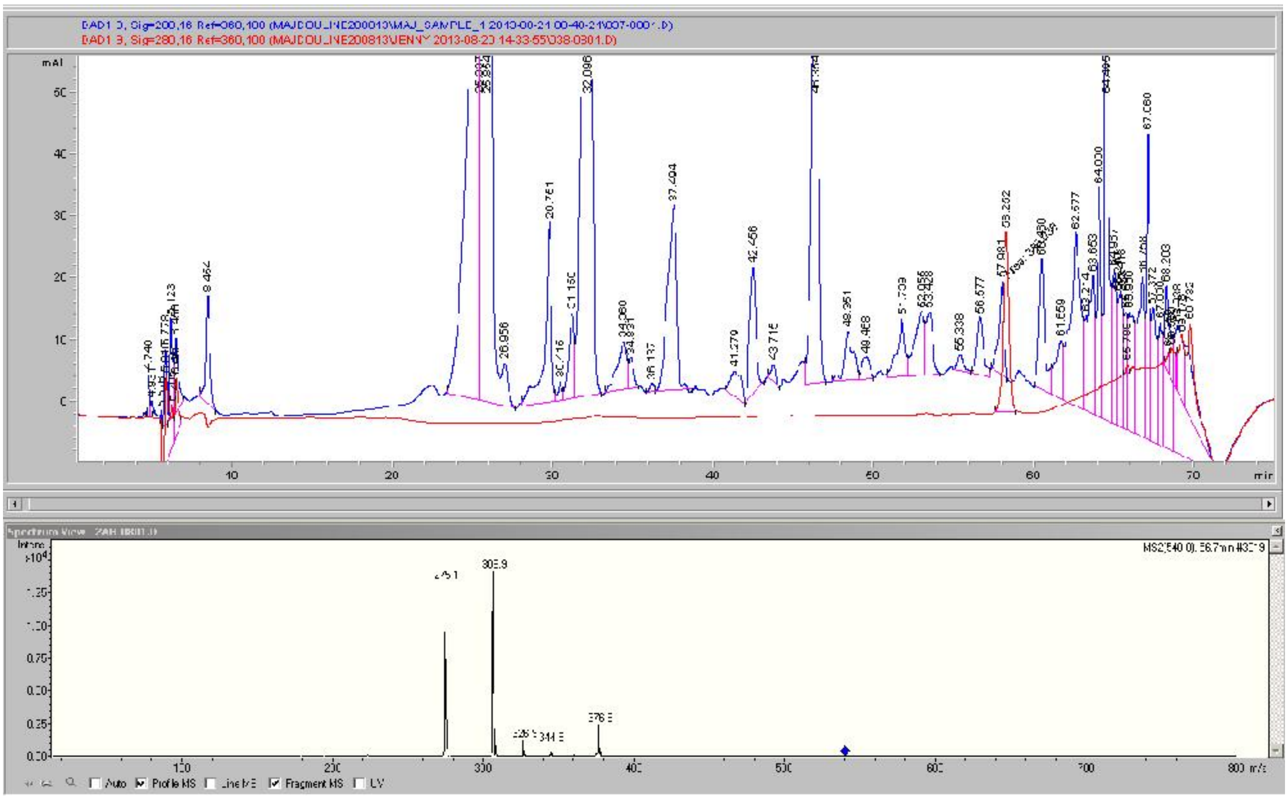

N_ferrulic acid (area=14.04, 35.28)

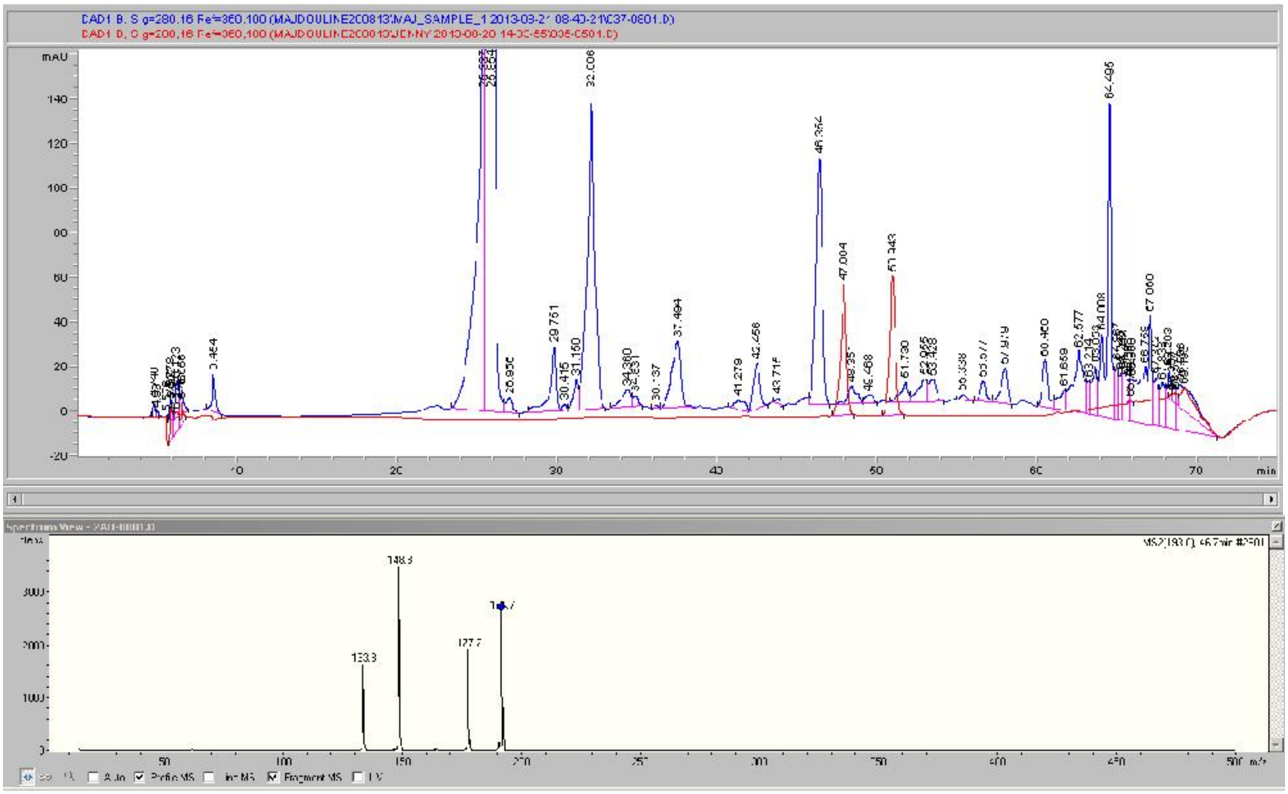

N_P-coumaric acid (area=222.965)

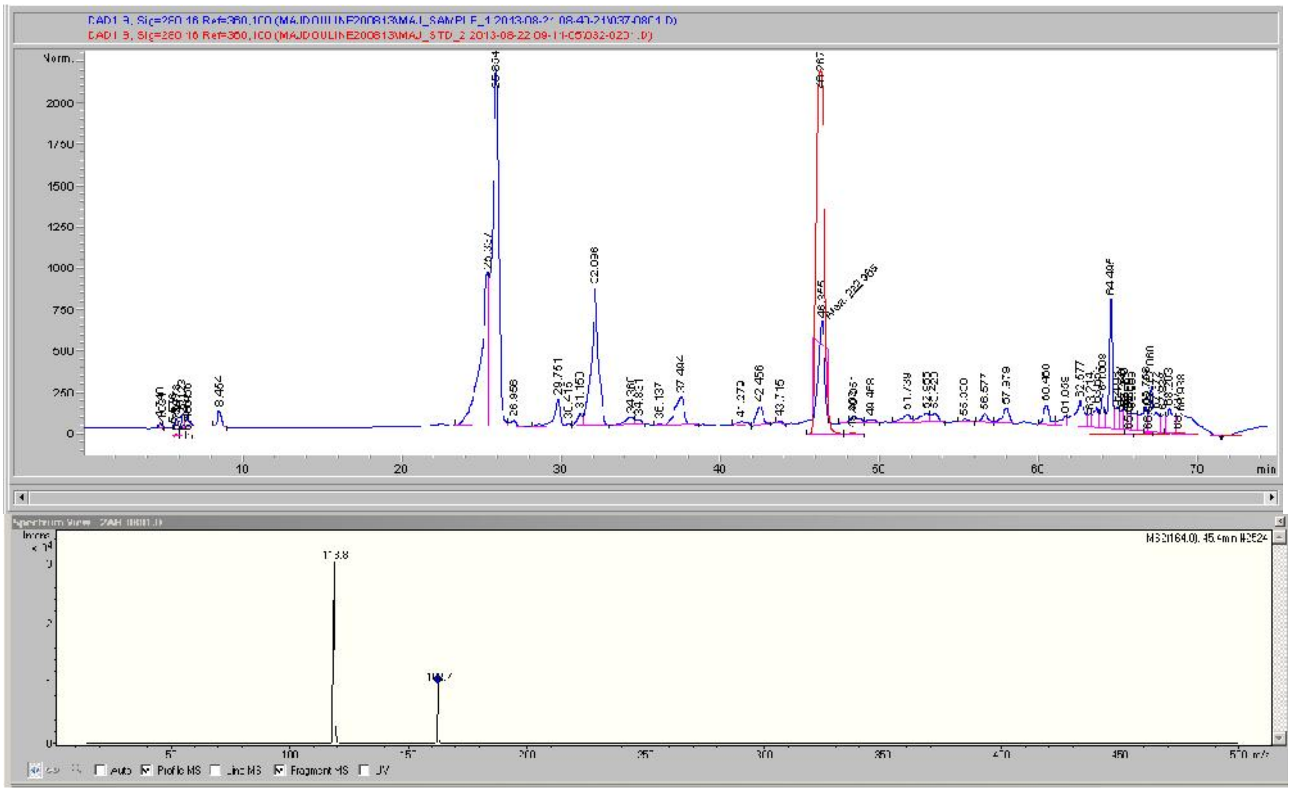

S1F1_Tyrosol (area=30.0784)

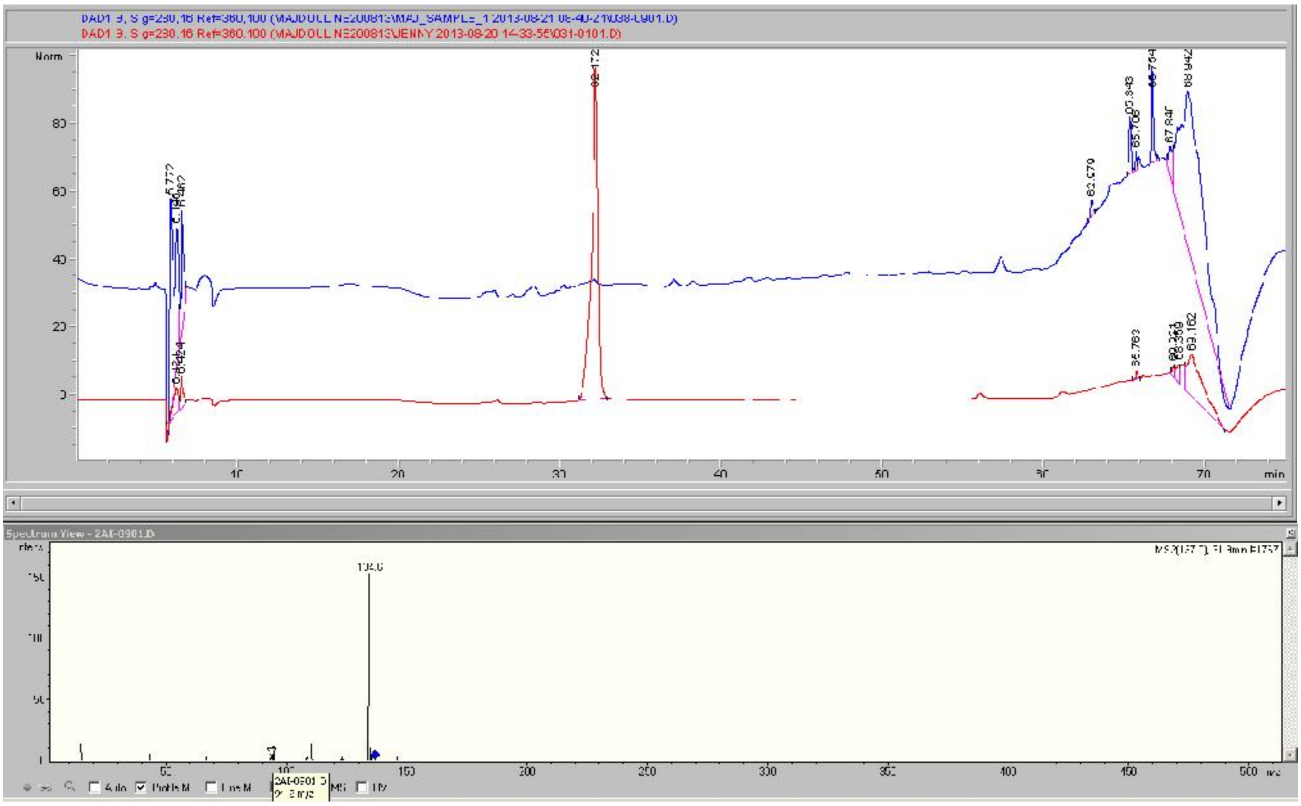

S1F6_Hydroxytyrosol (area=1075.7)

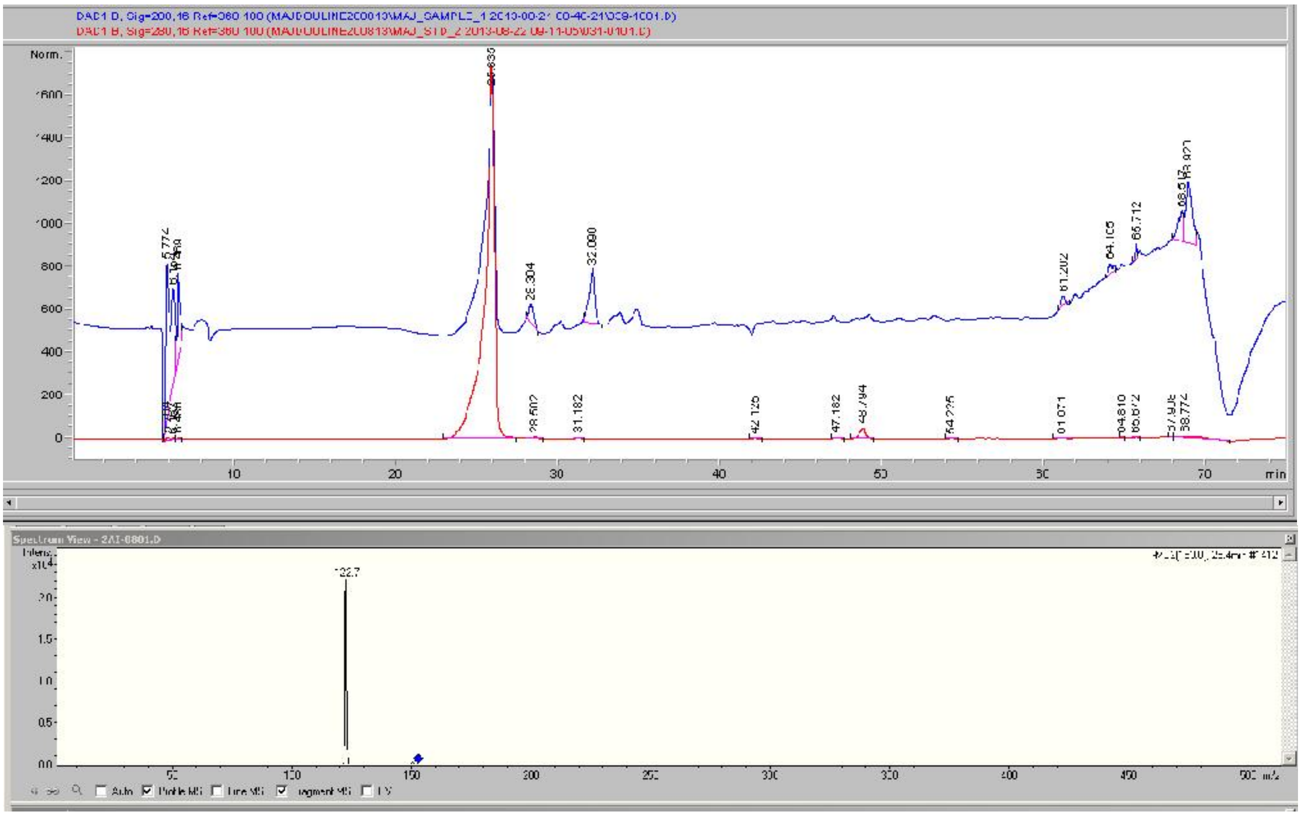

S1F6_Oleuropein (area=13.74)

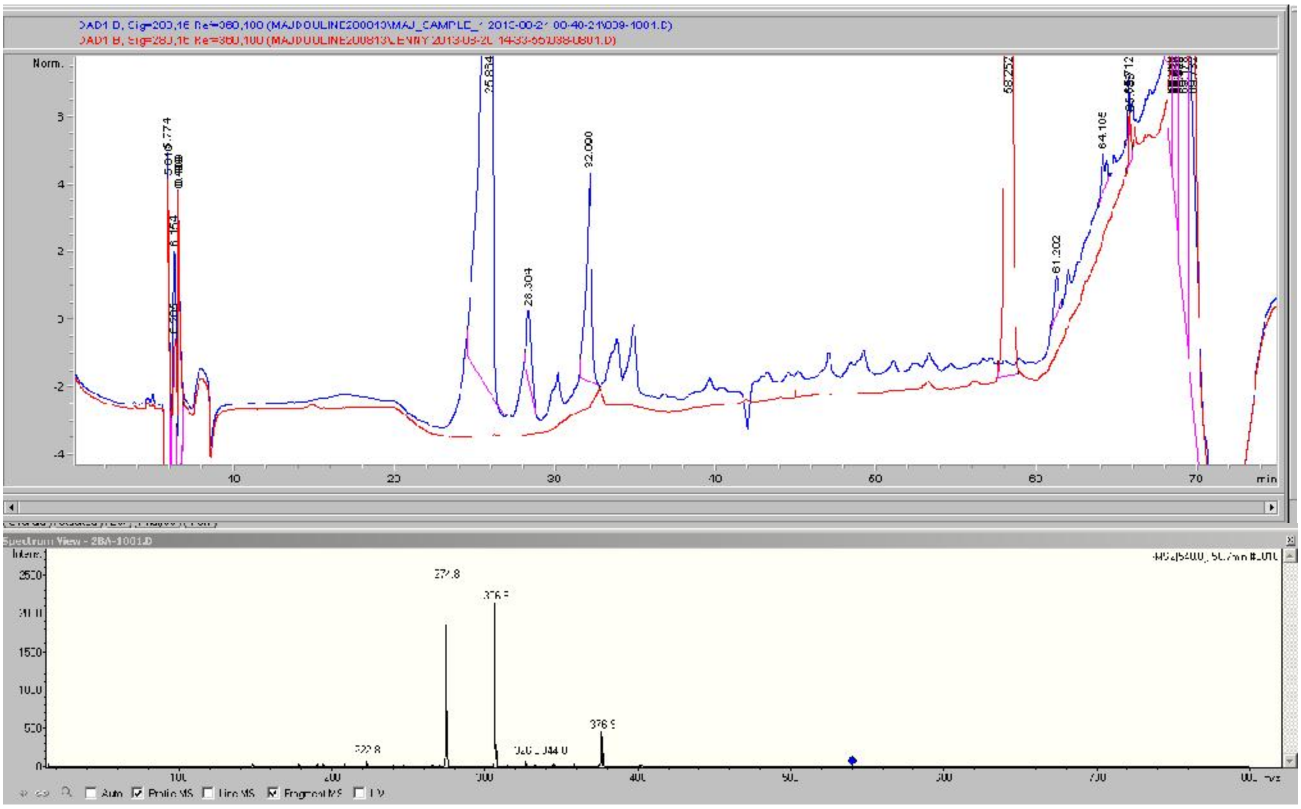

S1F7_Hydroxytyrosol (area=2085.74)

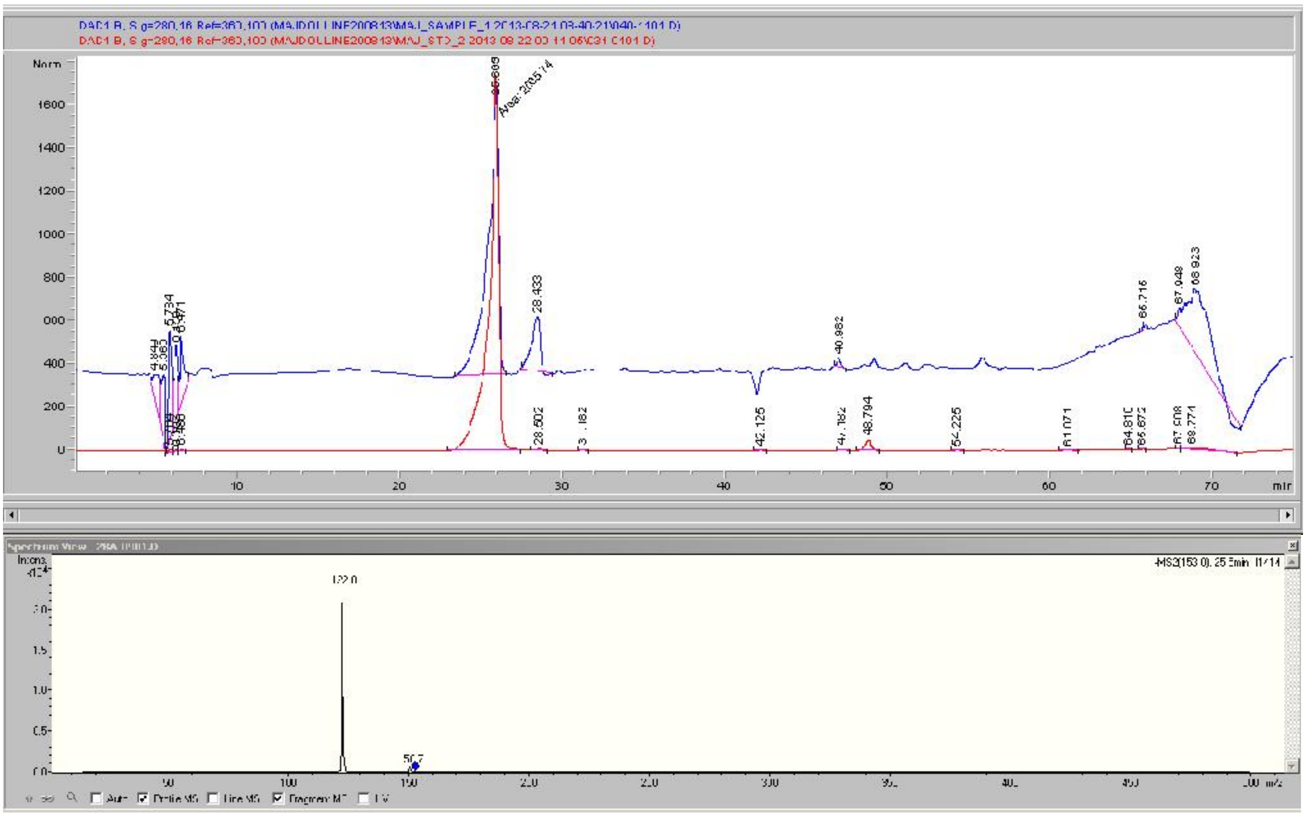

S1F8 = no confirmed compound

S1F9_oleuropein (area=54.1245)

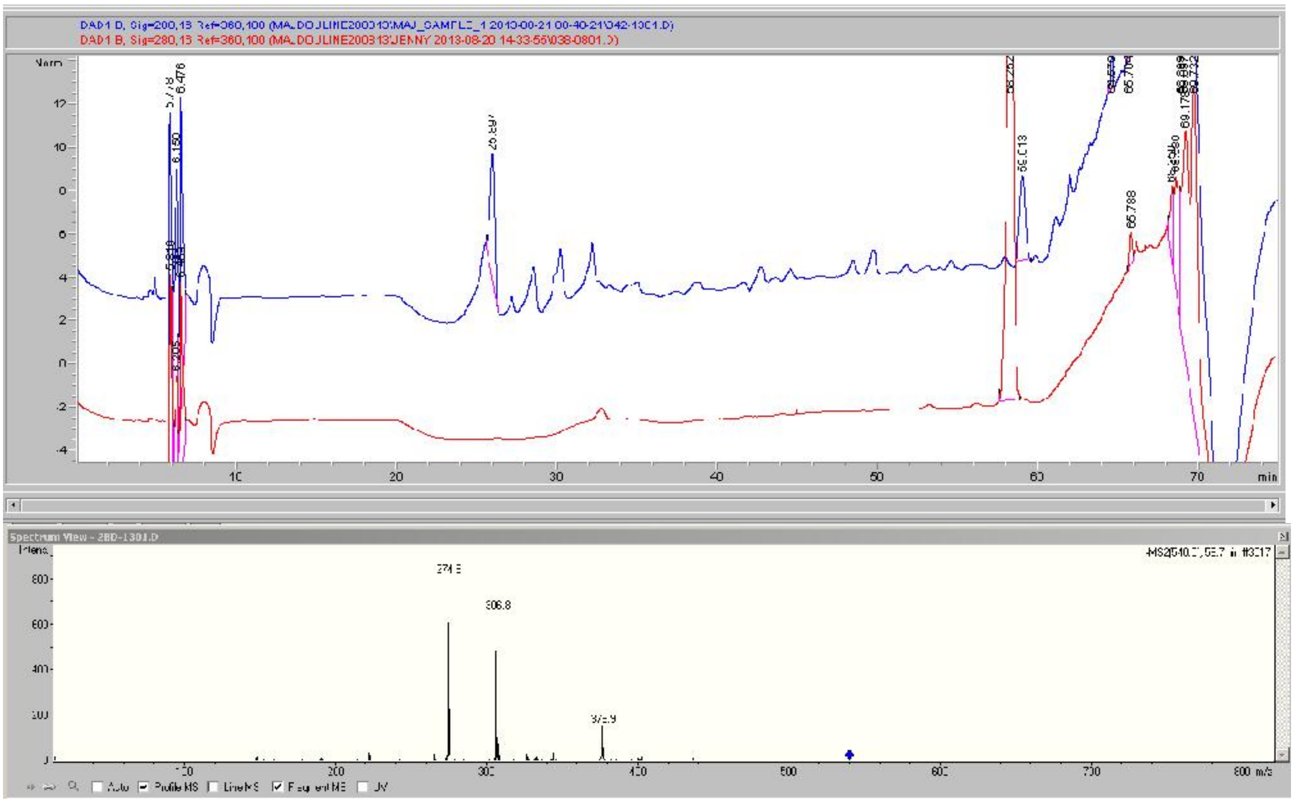

S2F2_Oleuropein (area=67.5804)

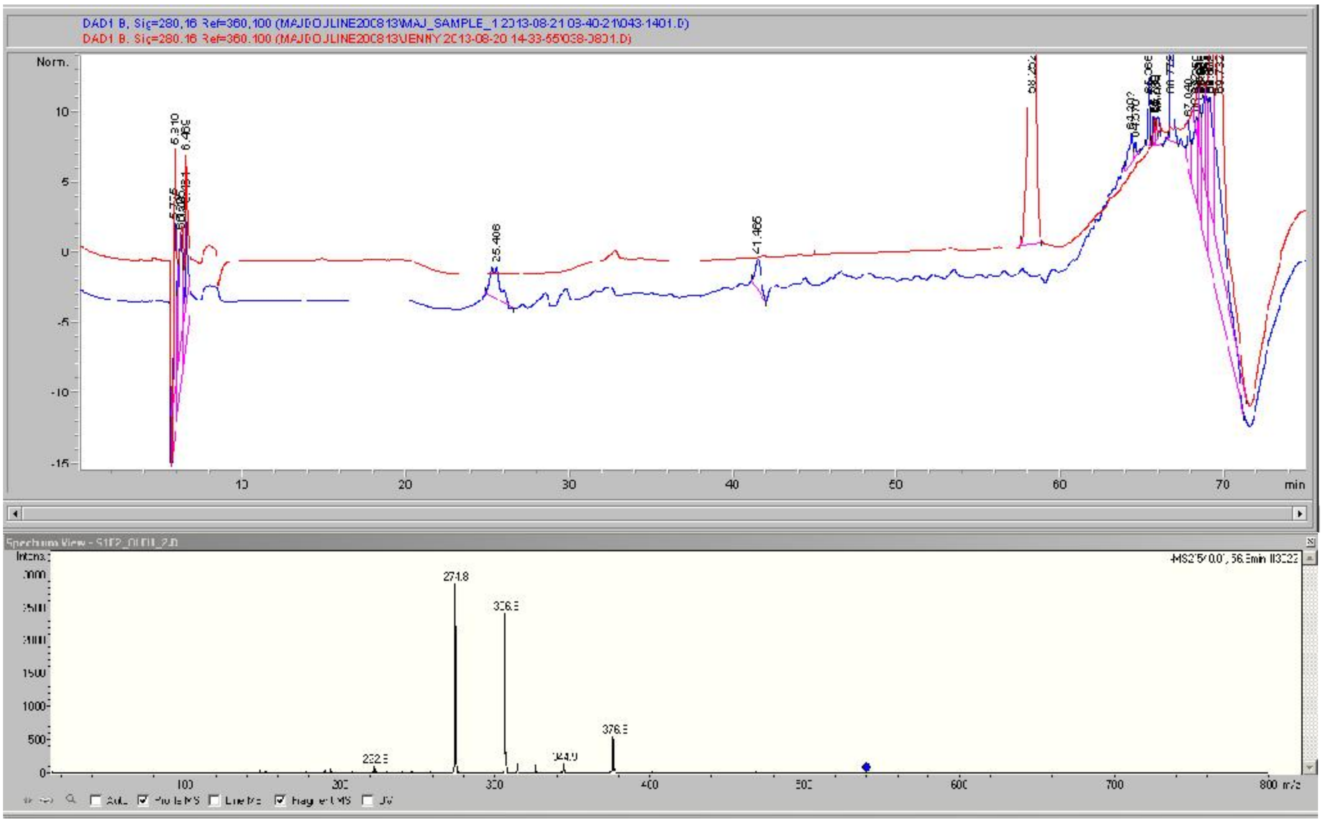

S2F4_tyrosol (area=39.4247)

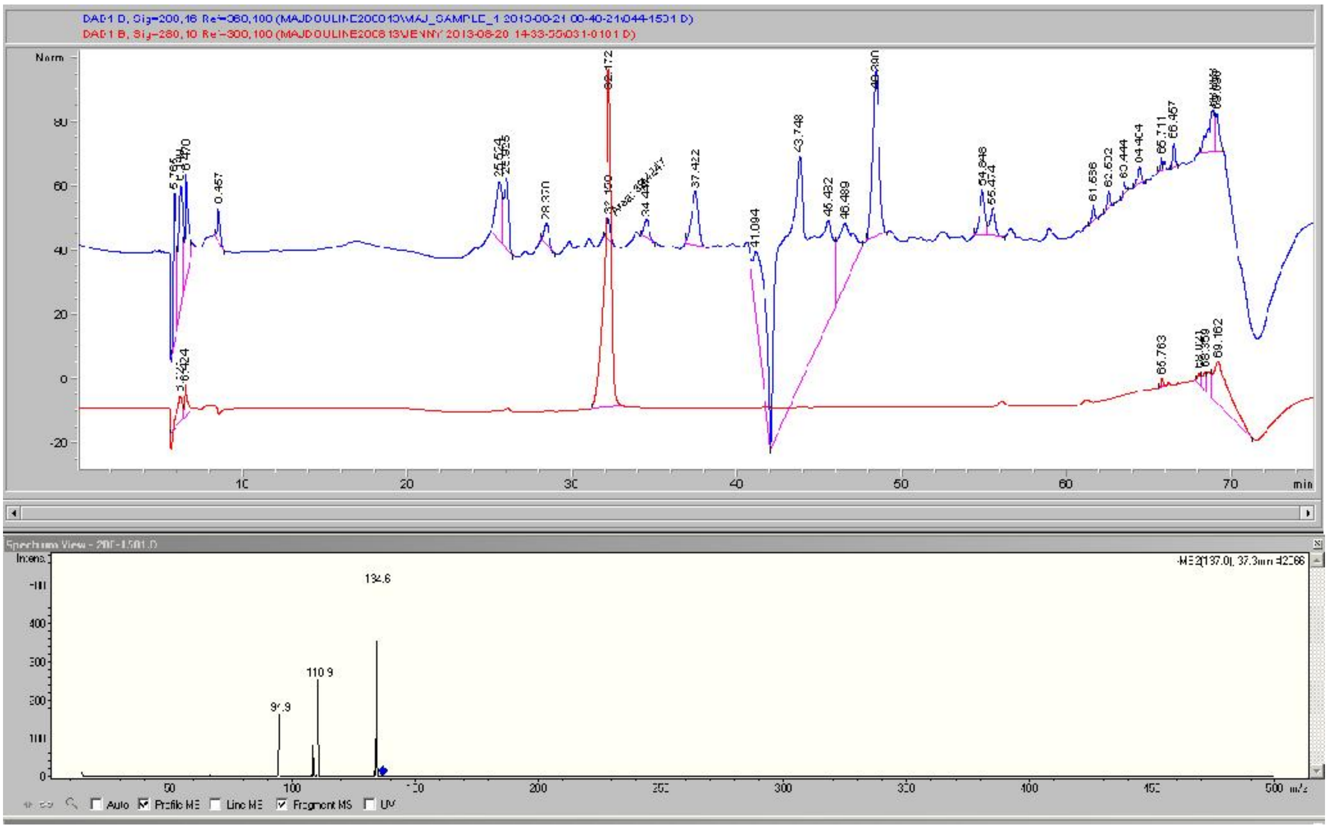

S2F4_Oleuropein (area=34.78)

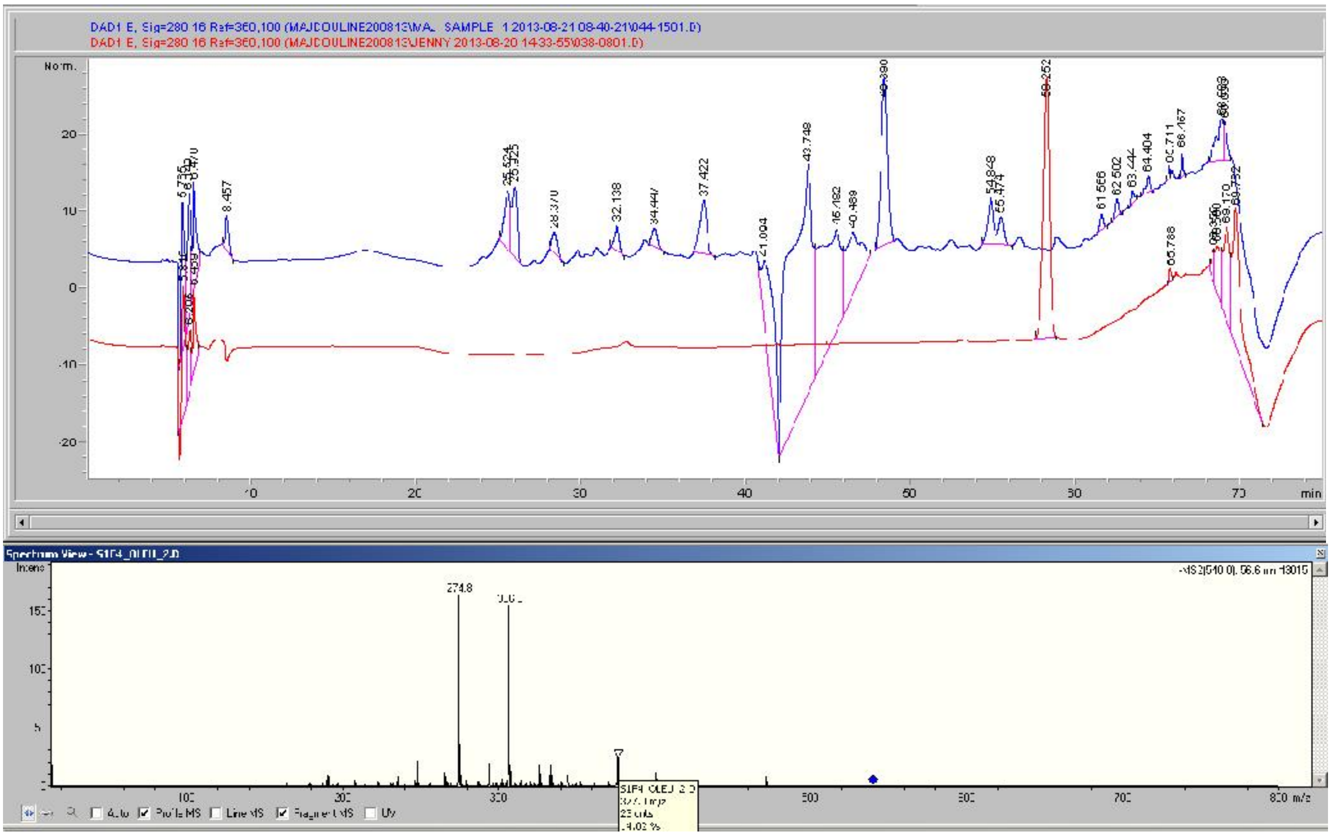

S2F4_P-coumaric acid (area=45.24)

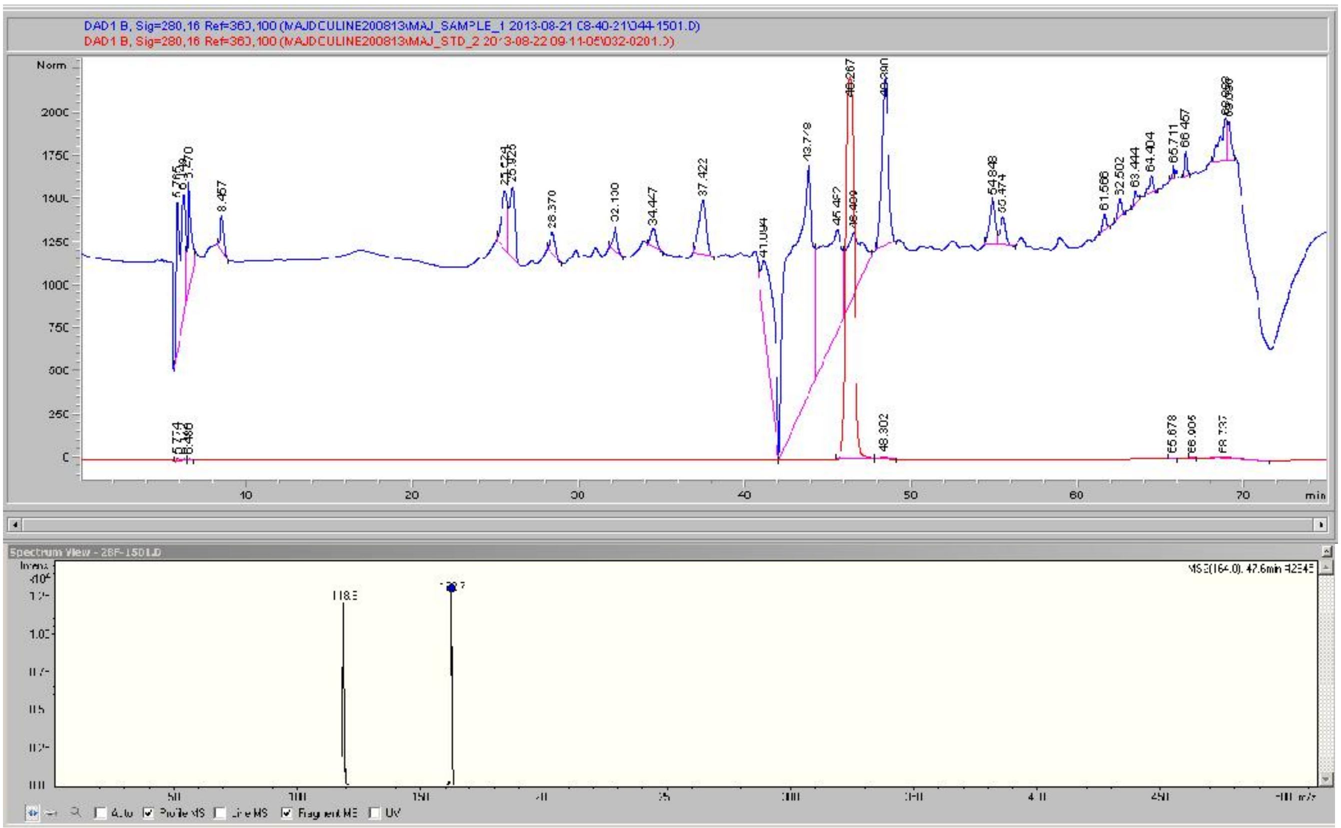

S2F5_hydroxytyrosol (area=1805.63)

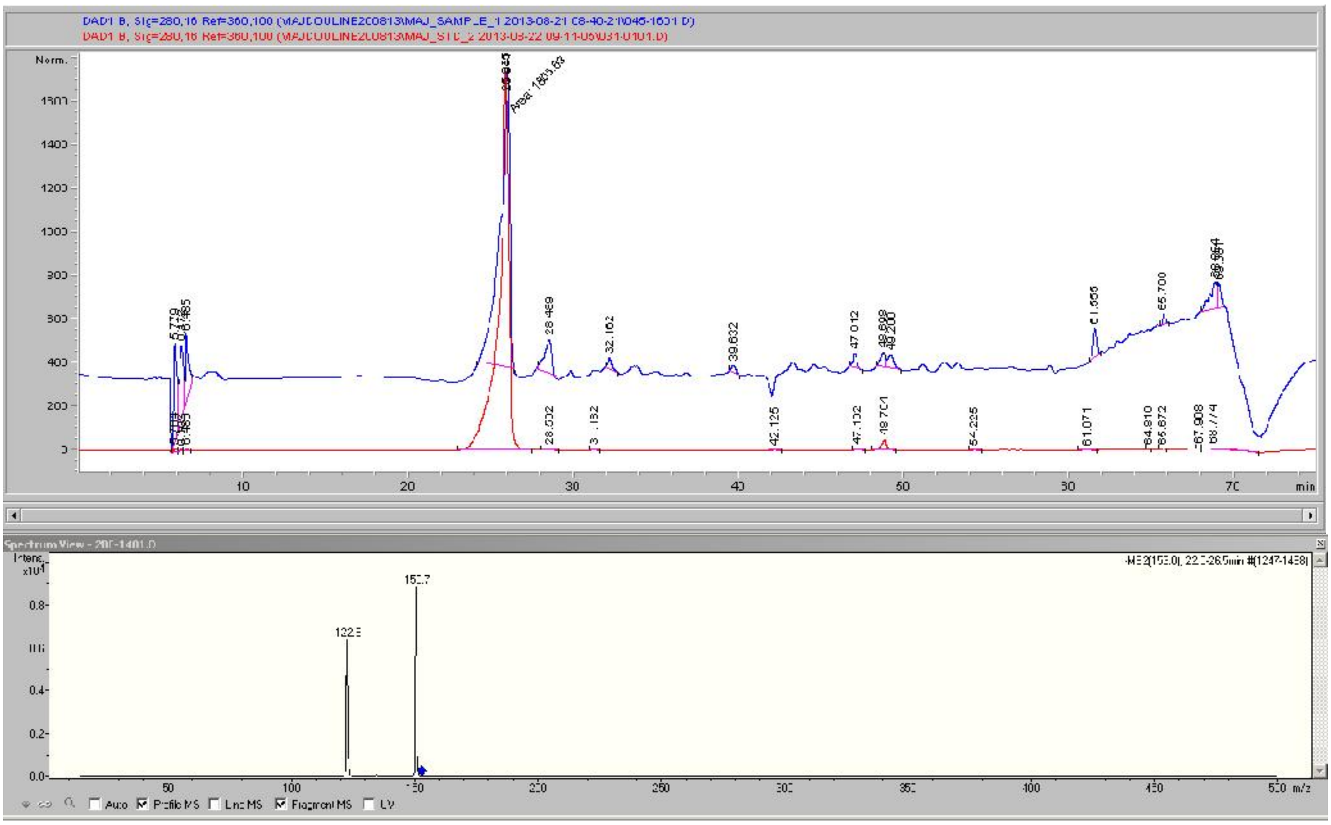

S2F5_oleuropein (area=53.03)

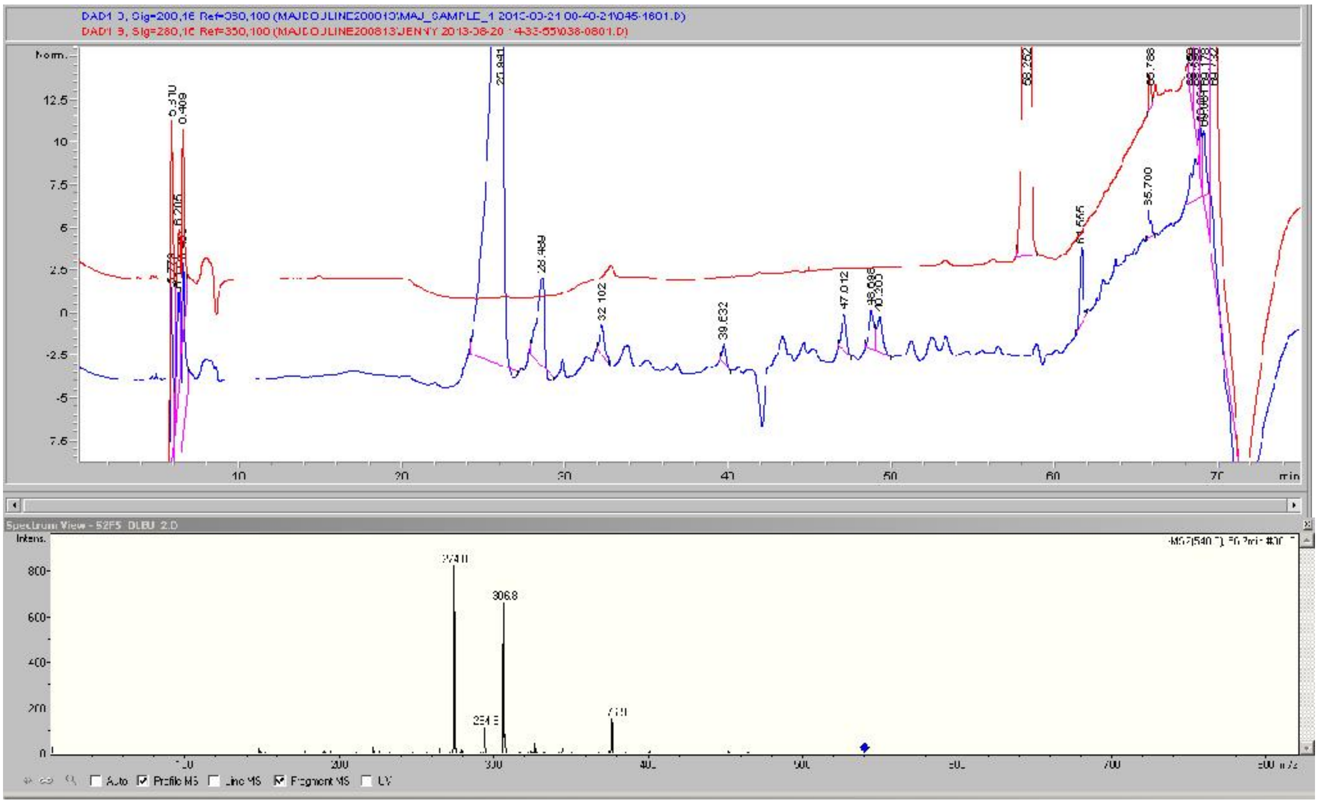

S2F6_hydroxytyrosol (area=325.776)

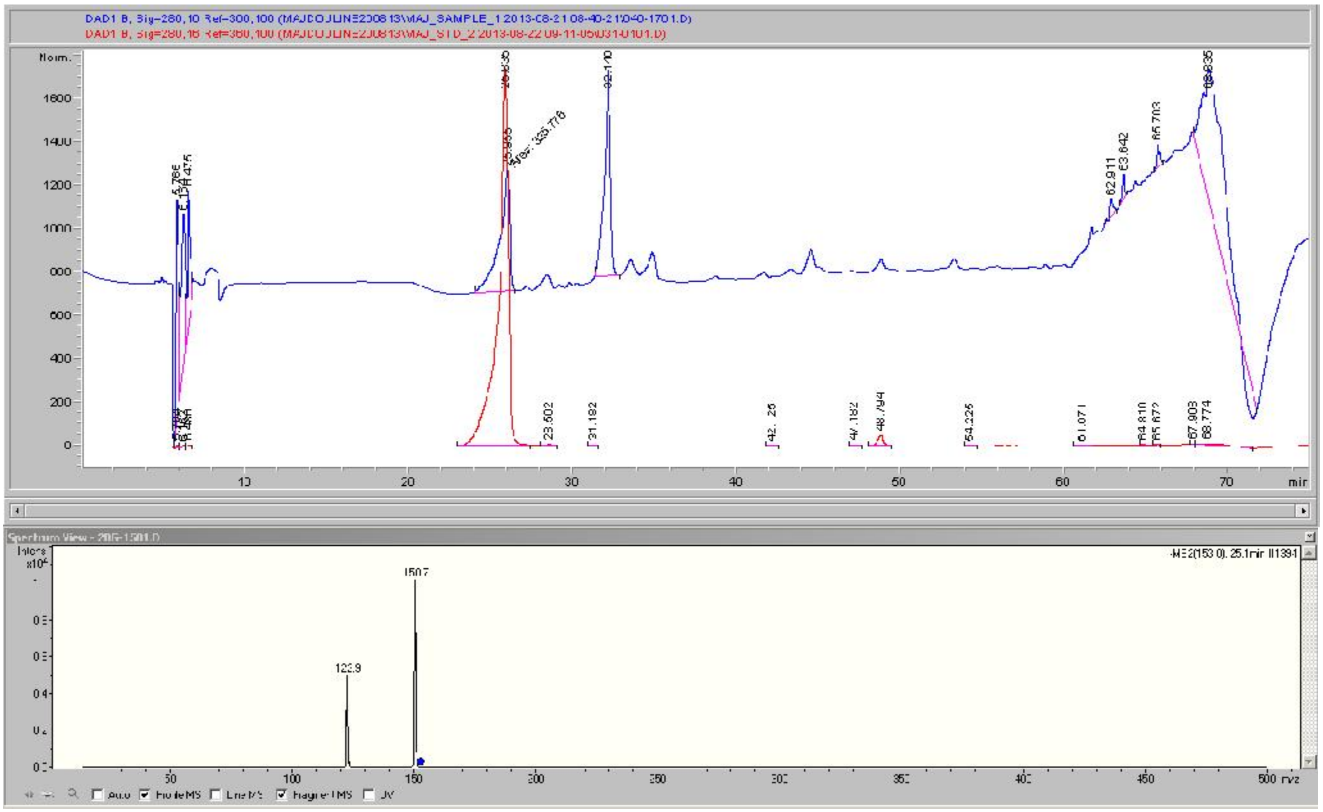

S2F6_Tyrosol (area=378.356)

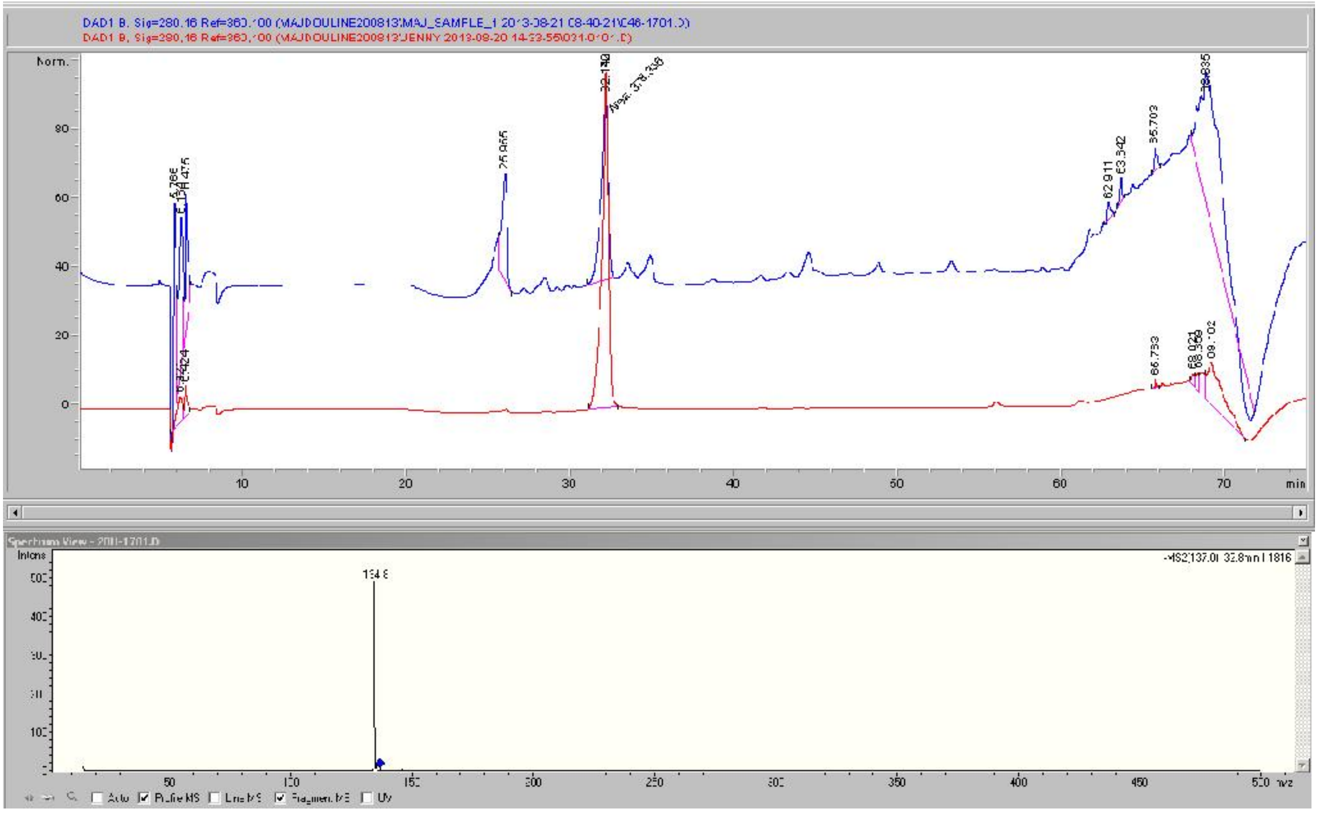

S2F7_tyrosol (area=1859.39)

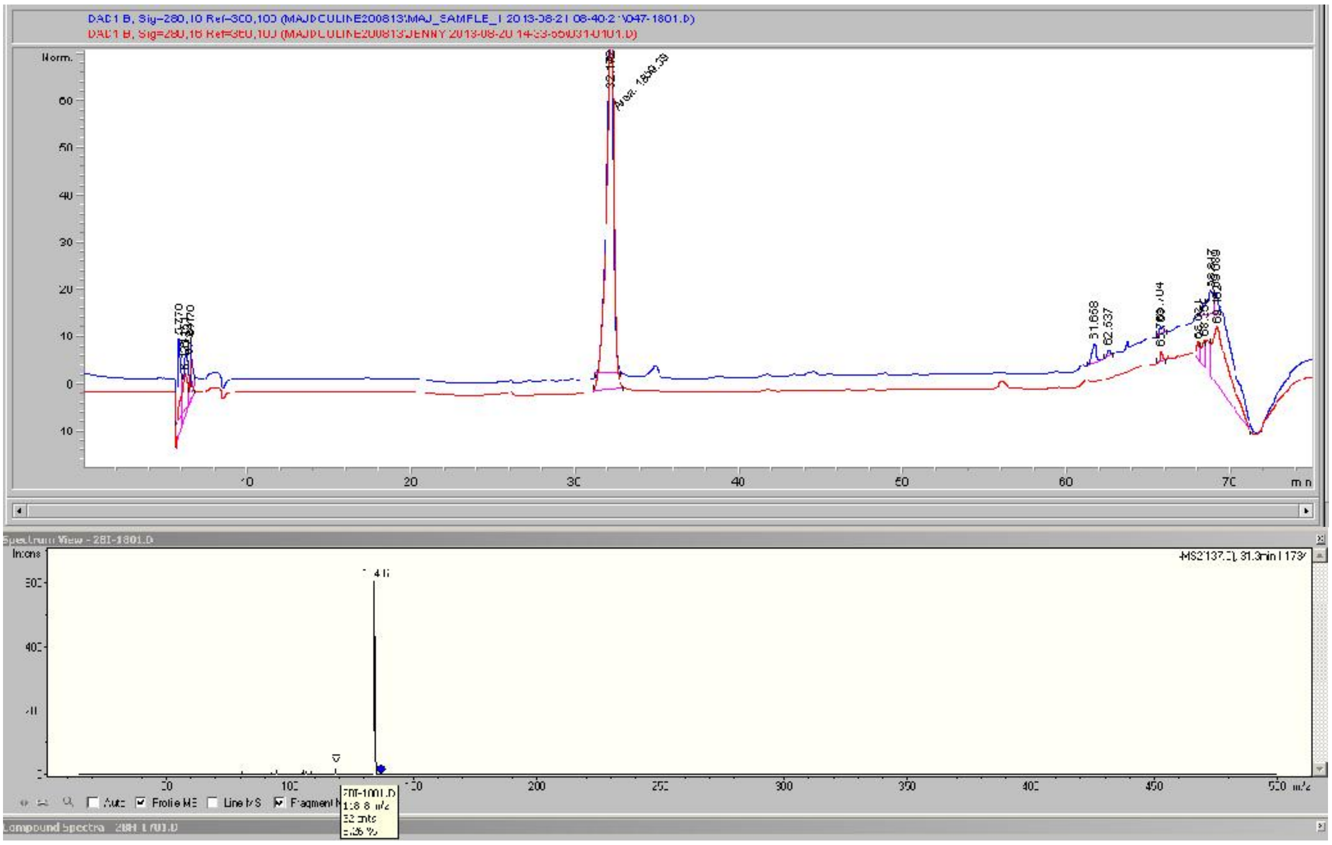

Supplement: S3 File — (PDF) [file pone.0182622.s004.pdf]
