# Supplementary material for: Assessment of the antioxidant and antibacterial activities of different olive processing wastewaters
Source: PLoS One. 2017 Sep 5;12(9):e0182622. doi: 10.1371/journal.pone.0182622 (PMC5584791; doi:10.1371/journal.pone.0182622)
Supplement: S1 Table — Diameter of zone of inhibition (mm) including diameter of 6 mm disc; Results quoted as the average of a minimum of six measurements ± standard deviation; “-”indicates no visible zone of inhibition. PC: positive control; NC: negative control; OMW1: phenolic extract of OMW from semi-modern three-phase process; OMW2: phenolic extract of OMW from modern three-phase process. (PDF) [file pone.0182622.s005.pdf]

S1 Table. Antibacterial activity of OMW phenolic extracts (1mg/disc)

Organism	Inhibition Zone (mm)*			
	Tested substances			
	PC	NC	OMW1	OMW2
Staphylococcus aureus	15 ± 0.5	-	10.66 ± 0.57	8 ± 0.5
Bacilus subtilis	16.66 ± 0.57	-	8 ± 0.5	7 ± 0.5
Echerichia coli	15.66 ± 1.15	-	7.5 ± 0.7	-
Salmonela thyphimurium	16 ± 0.5	-	7.5 ± 0.7	-
Lactobacilus lactis	17 ± 0.57	-	7 ± 0.05	-
Listiria innocua	18 ± 0.5	-	-	-
Lactobacilus balgaricus	23.33 ± 2.5	-	11 ± 1.73	9 ± 0.5

Diameter of zone of inhibition (mm) including diameter of 6 mm disc; Results quoted as the average of a minimum of six measurements ± standard deviation; "-" indicates no visible zone of inhibition. PC: positive control; NC: negative control; OMW1: phenolic extract of OMW from semi-modern three-phase process; OMW2: phenolic extract of OMW from modern three-phase process.
