# Supplementary material for: Assessment of the antioxidant and antibacterial activities of different olive processing wastewaters
Source: PLoS One. 2017 Sep 5;12(9):e0182622. doi: 10.1371/journal.pone.0182622 (PMC5584791; doi:10.1371/journal.pone.0182622)
Supplement: S2 Table — (PDF) [file pone.0182622.s006.pdf]

S2 Table. TLC data of OMW1 phenolic extract using different solvent ratios

Solvent System	Ratio	Concentration (mg/mL)	Number of bands	Rf Values
Hexane/Acetone	4:6	10	2	2.4;5.2
		25	2	3.3; 5
Chloroform/Methanol	10:1	10	2	2.2; 4.4
		25	3	3.2; 4.4; 5.7
Chloroform/Methanol	9:1	10	3	2.2;4;5.4
		25	3	2.8;3.4; 5.4
Chloroform/Methanol	4:1	10	2	4.1; 5.2
		25	2	4.5; 5.4
Chloroform/Methanol	8:2	10	2	4.5; 5.4
		25	1	5.5
Chloroform/Ethyl Acetate	1:1	10	2	1; 4
		25	2	2; 4
Hexane/Chloroform/Acetone	4:2:4	10	1	1.7
		25	2	3; 4.8
Chloroform/Dichlorobenzene/Acetone	5:4.5:0.5	10	1	0.9
		25	1	1.2
Hexane/Dichlorobenzene/Acetone/Methanol	4:4:1.5:0.5	10	2	1.3; 2.6
		25	2	1.3; 3
