# Supplementary material for: Assessment of the antioxidant and antibacterial activities of different olive processing wastewaters
Source: PLoS One. 2017 Sep 5;12(9):e0182622. doi: 10.1371/journal.pone.0182622 (PMC5584791; doi:10.1371/journal.pone.0182622)
Supplement: S3 Table — (PDF) [file pone.0182622.s007.pdf]

S3 Table. Summary results of the possible polyphenols from different olive processing wastewaters extracts as identified by HPLC analysis.

Sample	Retention time	Suspected compound	Area from HPLC	Concentration (µg/mg of extract)
OMW1	25.335	Hydroxytyrosol	23514.1	52.8
	32.187	Tyrosol	2351.8	6.88
	45.267	*p*-coumaric acid	72.4	0.16
	58.189	Oleuropein	562.3	31.36
OMW2	25.835	Hydroxytyrosol	15222.8	34.32
	32.161	Tyrosol	1194.2	3.48
	48.858	Ferrulic acid derivative	1855.9	5.72
	52.022	Ferrulic acid	131.7	0.48
	58.064	Oleuropein	132.8	7.4
	46.267	*p*-coumaric acid	613.0	2.72
GTOW	25.895	Hydroxytyrosol	74211.3	83.6
	32.172	Tyrosol	5939.1	8.7
	58.425	Oleuropein	23.8	0.66
PTOW	25.835	Hydroxytyrosol	43125.4	48.6
	32.094	Tyrosol	2209.0	3.24
	46.473	*p*-coumaric acid	282.3	0.32
	57.968	Oleuropein	1750.8	48.8
BTOW	25.868	Hydroxytyrosol	9663.5	10.9
	32.093	Tyrosol	4410.6	6.46
	57.981	Oleuropein	388.0	10.82
	47.884	Ferrulic acid derivative	14.0	0.02
	51.739	Ferrulic acid	35.3	0.06
	46.355	*p*- coumaric acid	223.0	0.24
